# Interpretable network-guided epistasis detection

**DOI:** 10.1093/gigascience/giab093

**Published:** 2022-02-04

**Authors:** Diane Duroux, Héctor Climente-González, Chloé-Agathe Azencott, Kristel Van Steen

**Affiliations:** BIO3 - Systems Genetics, GIGA-R Medical Genomics, University of Liège, 4000 Liège, Belgium, 11 Liège 4000, Belgium; Institut Curie, PSL Research University, F-75005 Paris, France; INSERM, U900, F-75005 Paris, France; CBIO-Centre for Computational Biology, Mines ParisTech, PSL Research University, 75006 Paris, France; High-Dimensional Statistical Modeling Team, RIKEN Center for Advanced Intelligence Project, Chuo-ku, Tokyo 103-0027, Japan; Institut Curie, PSL Research University, F-75005 Paris, France; INSERM, U900, F-75005 Paris, France; CBIO-Centre for Computational Biology, Mines ParisTech, PSL Research University, 75006 Paris, France; BIO3 - Systems Genetics, GIGA-R Medical Genomics, University of Liège, 4000 Liège, Belgium, 11 Liège 4000, Belgium; BIO3 - Systems Medicine, Department of Human Genetics, KU Leuven, 3000 Leuven, Belgium, 49 3000 Leuven, Belgium

**Keywords:** gene-gene interaction, inflammatory bowel disease, systems biology, biology-informed analysis, epistasis network

## Abstract

**Background:**

Detecting epistatic interactions at the gene level is essential to understanding the biological mechanisms of complex diseases. Unfortunately, genome-wide interaction association studies involve many statistical challenges that make such detection hard. We propose a multi-step protocol for epistasis detection along the edges of a gene-gene co-function network. Such an approach reduces the number of tests performed and provides interpretable interactions while keeping type I error controlled. Yet, mapping gene interactions into testable single-nucleotide polymorphism (SNP)-interaction hypotheses, as well as computing gene pair association scores from SNP pair ones, is not trivial.

**Results:**

Here we compare 3 SNP-gene mappings (positional overlap, expression quantitative trait loci, and proximity in 3D structure) and use the adaptive truncated product method to compute gene pair scores. This method is non-parametric, does not require a known null distribution, and is fast to compute. We apply multiple variants of this protocol to a genome-wide association study dataset on inflammatory bowel disease. Different configurations produced different results, highlighting that various mechanisms are implicated in inflammatory bowel disease, while at the same time, results overlapped with known disease characteristics. Importantly, the proposed pipeline also differs from a conventional approach where no network is used, showing the potential for additional discoveries when prior biological knowledge is incorporated into epistasis detection.

## Background

Genome-wide association studies (GWAS) have identified >70,000 genetic variants associated with complex traits [1]. Often these variants altogether do not explain the whole variance of a trait. A representative example is inflammatory bowel disease (IBD), like Crohn disease and ulcerative colitis. Pooled twin studies estimate their heritabilities at 0.75 and 0.67, respectively [2]. Yet, despite large GWAS that identified >200 IBD-associated loci [3], a low proportion of their variance has been explained [4]. Possible explanations include a large number of common variants with small effects, rare variants with large effects not covered in GWAS, unaccounted for gene-environment interactions, and genetic interactions [5]. In this article we explore the latter, called epistasis, which has been linked to IBD in the past [6–11]. Often, 2 types of epistasis are described: biological and statistical epistasis [12]. Broadly described, biological epistasis refers to a physical interaction between 2 biomolecules that has an effect on the phenotype. Statistical epistasis refers to departures from population-level linear models describing relationships between predictive factors such as alleles at different genetic loci.

Genome-wide association interaction studies (GWAIS) focus on the detection of statistical epistasis. To date, these studies have produced few replicable, functional conclusions, and specific gene-gene interactions have rarely been identified. This may be due to the small effect sizes of the interactions, the low statistical power, or the absence of a widely accepted GWAIS protocol. Even in the absence of statistical challenges, GWAIS are usually conducted on single-nucleotide polymorphisms (SNPs), and SNP-interactions often lack a straightforward functional interpretation. Moving from SNP- to gene-level tests, which jointly consider all the SNPs mapped to the same gene, might address both shortcomings. First, aggregating SNP pair statistics into gene pair statistics is likely to increase the statistical power when dealing with complex diseases [13]. Second, converting statistical findings into biological hypotheses [14] may facilitate their functional interpretability [15].

To both reduce the number of tests and improve the interpretablity of significant SNP-interactions, some authors propose examining only pairs of SNPs likely to be functionally related [16]. Such approaches use prior biological knowledge, e.g., of SNPs involved in genes that establish a protein-protein interaction [17]. Yet, limiting studies to 1 particular kind of gene-gene interaction might be reductive. To tackle that issue, Pendergrass et al. [18] developed Biofilter, a gene-gene co-function network, which aggregates multiple databases. Additionally, such approaches often require as well a proper mapping of SNPs to genes.

In this article, we propose guiding the search for statistical epistasis using plausible biological epistasis. Taking exclusively interactions reported from ≥2 different sources in Biofilter, we compile a subset of gene-gene interactions that are biologically plausible. Then, we exclusively search for those interactions in a GWAIS dataset, reducing the multiple test burden and improving the interpretability. We investigate different ways of mapping SNPs to genes and use the adaptive truncated product method [19] to estimate the association of gene pairs. Network and pathway analyses are used to further assist in the interpretation of epistasis findings. The proposed pipeline is applied to GWAS data from the International IBD Genetic Consortium [3].

## Data Description

We investigated the IIBDGC dataset, produced by the International Inflammatory Bowel Disease Genetics Consortium (IIBDGC). This dataset was genotyped on the Immunochip SNP array [20]. We performed quality control as in Ellinghaus et al. [21], thereby reducing the number of SNPs from 196,524 to 130,071. The final dataset contains 66,280 samples, of which 32,622 are cases (individuals with IBD) and 33,658 are controls. The large sample size of this dataset helps in overcoming the issue of reduced statistical power that is common in GWAIS.

The IIBDGC dataset aggregates different cohorts and contains potentially confounding population structure. As in Ellinghaus et al. [21], we used the first 7 principal components to model population stratification. Because several epistasis detection methods, such as those implemented in PLINK [22], cannot include covariates in their logistic regression models, we instead adjusted the phenotypes by regressing out those principal components. In other words, we derived adjusted phenotypes from the logistic regression model by subtracting model-fitted values from observed phenotype values, i.e., response residuals (see Supplementary Fig. S1).

## Analysis

### SNP to gene mapping: *Chromatin* contacts map more SNPs per gene than other mappings

**Figure 1:**
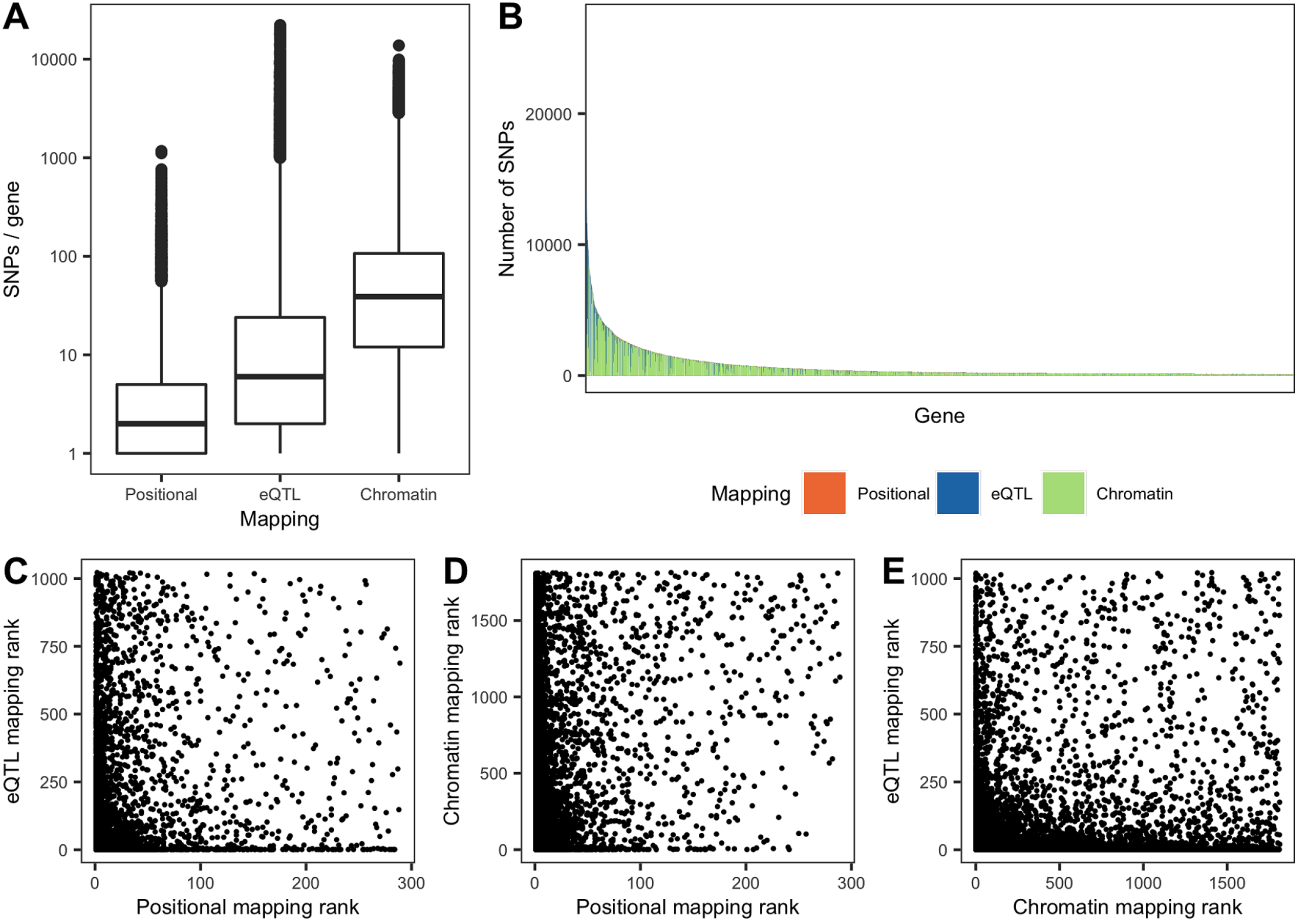
: (A) Number of SNPs per gene for each of the 3 mappings described in section “*From gene models to SNP models*.” Each box shows the median number of SNPs mapped to the same gene (the bold line in the middle), with the second and the third quartiles below and above it, respectively; the whiskers represent the first and fourth quartiles; the outliers are indicated separately. (B) Ranking of genes with most SNPs mapped using any of the mappings, colored by mapping. Only genes with >100 SNPs mapped to it are displayed. (C–E) Comparison between the rank of each gene according to the number of SNPs mapped to it using each mapping.

In this article, we present a pipeline to detect gene epistasis across the edges of a network. We extract interacting pairs of genes from the gene-gene Biofilter network to obtain candidate gene epistatic pairs (“*gene models*”). We considered 3 ways to match genes to SNPs and obtain “*SNP models*” from them: “*Positional*,” “*eQTL*,” and “*Chromatin*” (detailed in section “*From gene models to SNP models*”). *Chromatin* produced the largest number of unique SNP-gene mappings (2,394,590), 1 order of magnitude more than *eQTL* (411,120) and *Positional* (174,879) (Table 4). The *Chromatin* mapping had on average the largest number of SNPs mapped on to a gene, followed by *eQTL* and *Positional* (Fig. 1A). Nonetheless, the number of SNPs mapped to a gene varied considerably across genes (Fig. 1B). In addition, the number of SNPs mapped to a same gene varied considerably across mapping methods (Fig. 1 C–E): in general, the genes with most SNPs mapped using the *eQTL* mapping had relatively few SNPs mapped in the *Chromatin* mapping, and vice versa.

### The *Positional* analysis does not recover any SNP interaction

The aforementioned SNP-gene mappings, and combinations of them (cross-mappings), yielded 7 sets of SNP models. Running our pipeline on them resulted in 7 epistatic SNP-SNP networks described in Table 1 (for visualization, see Supplementary Fig. S2). We also conducted what we called a “*Standard*” analysis, which reflects a conventional epistasis detection procedure. In this one, we exhaustively searched for epistatic interactions between all the SNPs that passed quality control. Then, we used positional mapping to assign gene interactions to the significant SNP-interactions. Strikingly, while the *Standard* analysis generated the largest SNP-interaction network (55 nodes/SNPs and 57 edges/interactions), the *Positional + eQTL* one was the largest by number of interactions (76). The *Positional* analysis produced no significant interactions at all.

**Table 1:** Properties of the SNP networks obtained from different datasets

Analysis	SNPs	Edges	Components	Mean degree
*Standard*	55	57	12	2.07
*Positional*	0	0		
*eQTL*	46	64	6	2.78
*Chromatin*	20	19	5	1.9
*eQTL + Chromatin*	44	48	8	2.2
*Positional + eQTL*	43	76	6	2.7
*Positional + Chromatin*	21	39	5	1.9
*Positional + eQTL + Chromatin*	39	45	6	2.3

Nodes are SNPs, which are linked when the SNP model is significant.

### Gene epistasis: “functional” mappings boost discovery and interpretability

**Figure 2:**
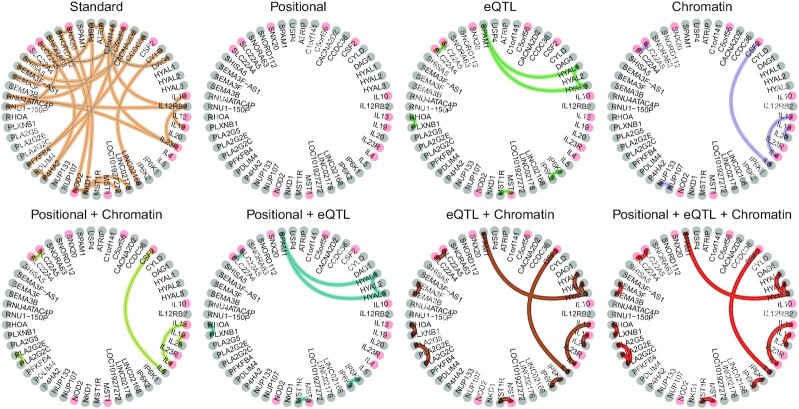
: Epistasis networks built from derived significant gene models for the different analysis strategies. Genes associated with IBD in DisGeNET [23] are indicated in pink. An alternative layout of the networks is available in Supplementary Fig. S3.

Findings of a GWAIS are often presented as a network, with nodes indicating SNPs and edges between nodes being present when the analysis protocol identifies the corresponding SNP pair as significantly interacting with the trait of interest. We converted SNP model networks into gene model epistasis networks (Fig. 2), adding an edge between 2 genes whenever the corresponding gene model significance was ascertained through an adaptive truncated product method (ATPM) approach. The largest network was obtained under the *Standard* analysis (26 edges, Table 2). The *Positional +eQTL+ Chromatin* combinations performed second best (13 edges). Because no significant SNP pairs were detected under Positional, no significant gene pairs were produced either. Similarly, the 2 analyses that included Positional information on top of another source barely added any new information in comparison to *Positional +eQTL+ Chromatin*: *Positional +eQTL* included a new gene pair (*SPAM1-HYAL1*, already detected in the *eQTL* analysis), while *Positional + Chromatin* did not produce any additional pair. On the same line, *eQTL+ Chromatin* included only 1 gene model absent from *Positional +eQTL+ Chromatin*: *PLA2G2E-PLA2G2C*. Hence, we removed those 3 from further analyses because *Positional +eQTL+ Chromatin* captured more biological signal than those separately.

**Table 2:** Properties of the gene networks obtained from different datasets

Mapping	Genes	Edges	Components	Mean degree
*Standard*	29	26	8	1.79
*Positional*	0	0		
*eQTL*	11	7	5	1.27
*Chromatin*	10	5	5	1
*eQTL + Chromatin*	22	12	10	1.1
*Positional + eQTL*	11	7	5	1.3
*Positional + Chromatin*	10	5	5	1
*Positional + eQTL + Chromatin*	23	13	10	1.1

Nodes are genes, which are linked when the corresponding gene model is significant.

**Figure 3:**
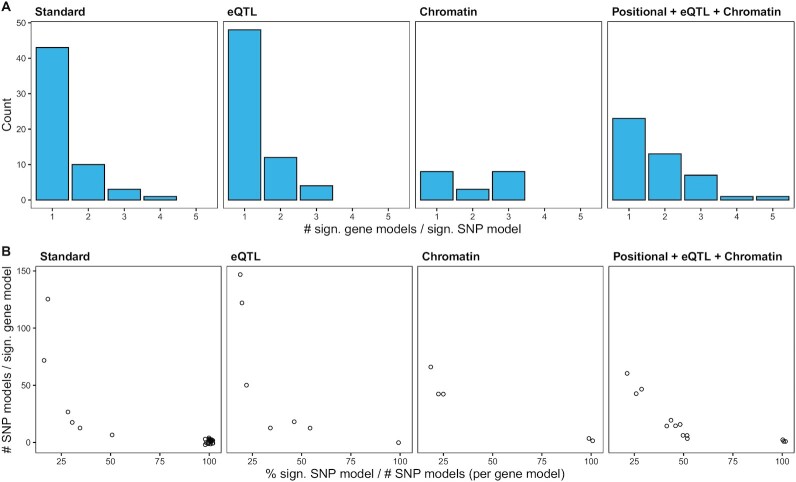
: Relationship between the number of significant SNP models and the number of significant gene models. (A) Histogram of the number of significant gene models mapped to the same significant SNP model. (B) Relationship between the total number of SNP models mapped to the same significant gene model (*y*-axis), and the percentage of all the SNP models mapped to the same significant gene model that are significant themselves (*x*-axis). Because multiple points can stack, we introduced a little Gaussian noise on each of them to improve visualization.

For both *eQTL* and *Standard* most of the significant SNP models mapped to exclusively 1 gene model, removing possible sources of ambivalence (Fig. 3A). That was less the case under the *Chromatin* analysis, where it was more common for the same SNP model to map to different gene models. We also investigated the relationship between significant gene models and the number of significant SNP models that mapped to them (Fig. 3B). Most significant gene interactions were supported by relatively small numbers of SNPs: either few in number, or few with respect to the total number of SNP models for that significant gene model.

### Significant SNP pairs are near each other and near loci with main effects

**Figure 4:**
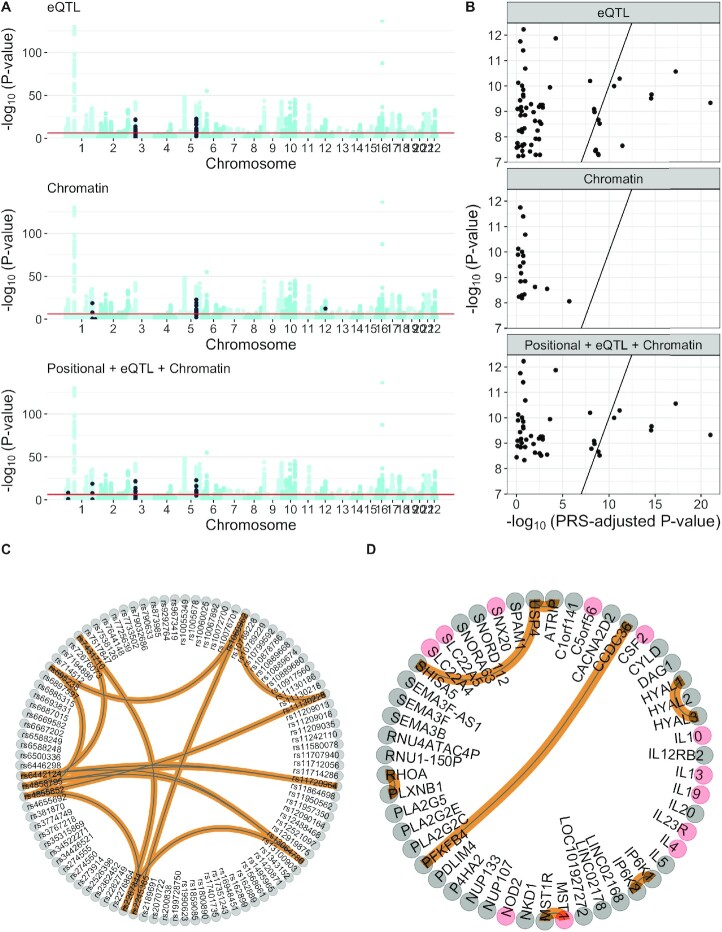
: (A) Manhattan plot of the main effects, computed using logistic regression. In each subpanel, the SNPs selected via a significant SNP model, by each analysis, are indicated in black. For reference, the Bonferroni threshold of main effects significance is displayed with a red horizontal line. (B) Comparison between the *P*-values of the significant SNP-interactions, adjusted and unadjusted by main effects (*x*- and *y*-axis, respectively). *P*-values were not adjusted for multiple testing. To help interpretation, we added a *y* = *x* line. (C) Network containing all the SNP models significant in any of the analyses whose *P*-values after adjusting for PRS were lower than the original *P*-values. (D) Network containing all the gene models significant in any of the analyses that were mapped to 1 of the significant SNP models from panel C in its corresponding analysis. Genes associated with IBD in DisGeNET [23] are indicated in pink.

Notably, the SNPs in significant SNP-interactions are located near each other in the genome (the median distance between the pair of SNPs in *Chromatin, eQTL*, and *Positional +eQTL+ Chromatin* was 161 kb). Moreover, they tend to overlap with GWAS main effects loci (Fig. 4A). To investigate whether main effects could be driving some of the signals, even when in imperfect linkage disequilibrium (LD) with epistatic SNP pairs (a phenomenon sometimes referred to as “phantom epistasis” [24]), we conducted a linear regression-based test, including a vector of polygenic risk scores as covariate. The observed effect of many significant SNP models notably decreased when we conditioned on single SNPs in this way (Fig. 4B), but not for all. The latter suggests a masking effect opposite to phantom epistasis. However, it is unclear how to adequately correct for multiple hypotheses testing after this adjustment in our setting, and in what follows we still use the unadjusted *P*-values, with the understanding that some of them may be inflated by weak correlations with main effects.

### The type I error is controlled

To evaluate the statistical relevance of the detected gene interactions, we studied whether the proposed protocol controlled the type I error. For that purpose, we performed a permutation analysis based on 1,000 permutations for each of the datasets, permuting the phenotypes and running the entire protocol to detect significant gene interactions. This permutation procedure is independent of the one used in the proposed protocol to compute significance thresholds. When ≥1 significant gene interactions was observed in a permutation, that permutation was considered a false positive (FP) result. This allowed us to compute the type I error rate as #FP/1,000. Type I error was under control in all tested experimental settings, with estimates ≤6.6% (Table 3).

**Table 3:** Type I error of the protocol presented in section “*Gene interaction detection procedure*,” estimated over 1,000 random permutations

Analysis	Mean No. of significant interactions under *H*_0_	Type I error (%)
*Standard*	0.05	3.6
*Positional*	0.04	3.7
*eQTL*	0.09	4.2
*Chromatin*	0.07	6.6
*Positional + eQTL + Chromatin*	0.04	3.6

### Biofilter boosts discovery of interpretable hypotheses

**Figure 5:**
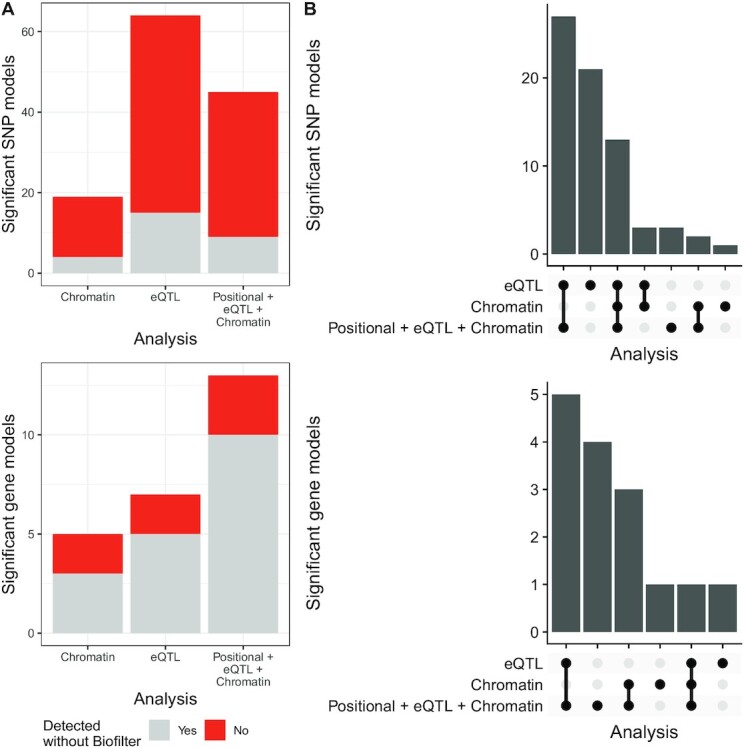
: Comparison of the proposed analysis with the relaxation of filters at different stages. (A) Effect of focusing on interactions mappable to Biofilter interactions. Proportion of the significant interactions that were detected using only the SNPs mappable to a Biofilter interaction, and using all of them. SNP interactions on top; gene interactions at the bottom. (B) Effect of focusing on 1 SNP-gene mapping at a time, or multiple mappings at once. Overlap between the significant interactions detected in the different analyses. SNP interactions on top; gene interactions at the bottom.

Searching for epistatic interactions exclusively across edges of the Biofilter network greatly reduces the number of tests. Yet, this gain in statistical power might not lead to greater discoveries because it potentially disregards new interactions absent from databases. Hence, we tested whether we would obtain similar results by exhaustively searching for epistasis on the datasets not reduced for Biofilter models but using each mapping. At the SNP level (Fig. 5A, upper panel), only a small proportion of the significant interactions were still detected when the network was not used. Strikingly, that difference got smaller at the gene level (Fig. 5A, lower panel). This suggests that the significant SNP models, even if fewer in number, are strong enough to lead to the detection of the gene models.

In a similar vein, we studied the overlap between the significant models detected in the different analyses. Including more SNP-gene mappings in the analysis was mostly beneficial with respect to considering 1 mapping at a time because both at the gene and at the SNP level, the significant interactions in *Positional +eQTL+ Chromatin* highly overlapped with the other analyses (Fig. 5B). Nonetheless, a few interactions were also missed in this joint analysis, in particular 20 significant SNP models detected in the *eQTL* analysis.

### *Positional +eQTL+ Chromatin* and *Standard* analyses partially replicate previous studies on IBD

In the past, several genetic studies have investigated epistasis on IBD [6,7,9–11,25]. We compared them to our results at the gene level, the minimal functional unit at which we expect genetic studies to converge. Several epistatic alterations have been reported involving interleukins [6,10,11]. Also our *Standard* analyses resulted in interactions involving 3 interleukins (*IL-19, IL-10*, and *IL-23*), although interacting with different genes than in the aforementioned studies. *Positional +eQTL+ Chromatin* recovered 5 interleukins (*IL-4, IL-5, IL-13, IL-19, IL-20*). In addition, Lin et al. [25] detected interactions involving *NOD2*, with both *IL-23R* and other genes. Our *Standard* analysis highlighted 2 potentially new epistasic interactions involving *NOD2*.

Discoveries in the proposed protocol are guided by plausible biological interactions. Hence, every significant gene model can be traced back to a biological database, therefore producing biological hypotheses. For instance, the gene model *MST1*-*MST1R* is significant in multiple pipelines. Both genes have been linked to IBD, both by themselves [26,27] and in interaction with other genes [28]. MST1R is a surface receptor of MST1, and, through physical interaction, they play a role in the regulation of inflammation.

### Pathway analyses highlight the involvement of the extracellular matrix in IBD

Pathway enrichment analyses of each interaction’s neighborhood allowed us to identify broader biological mechanisms in which the significant interaction pairs might be involved. The *eQTL* analysis produced multiple significant pathways (see Supplementary Table S1), involving the triangle of interactions formed by 2 genes located in 3p21.31 (*HYAL1, HYAL3*) and 1 in 7q31.32 (*SPAM1*) (Fig. 2). The affected pathways were related to the extracellular matrix, and specifically to glycosaminoglycan degradation. Links between the turnover of the extracellular matrix and IBD-related inflammation have been reported [29]. More specifically, glycosaminoglycan [30] and hyaluronon [31] degradation products lead to inflammatory response. When attention is restricted to pathways of minimum gene size 10 and maximum gene size 500 to avoid imbalances and non-normality, 4 pathways are removed: cellular response to UV B, hyalurononglucosaminidase activity, hexosaminidase activity, and CS/DS degradation. The *Chromatin* mapping and the *Standard* pipeline did not produce significant pathways. The *Positional +eQTL+ Chromatin* analysis produced 71 significant pathways (Supplementary Table S2), involving the neighborhoods *HYAL3, HYAL1, HYAL2* and *PLA2G2E, PLA2G5, PLA2G2C*.

### The proposed pipeline increases robustness

We studied whether our proposed pipeline led to more robust results. For that purpose, we ran the whole protocol again on a random subset of the data containing 80% of the samples. We repeated this experiment 10 times for each SNP-gene mapping. In each subset, 49% of the individuals were cases, respecting the initial proportion of cases and controls of the entire dataset. Conservatively, we used the same SNP and gene significance thresholds as for the corresponding entire dataset.

The *Standard* pipeline, which does not include Biofilter network-information, produced on average 11.4 significant gene models (SE 1.1). With the *eQTL* (respectively *Chromatin*) analysis, we detected on average 5.8 gene pairs (respectively 3.2) with SE 0.1 (respectively 0.4). With the *Positional +eQTL+ Chromatin* mappings process, we detected a mean of 8.6 gene pairs (SE 1.3).

**Figure 6:**
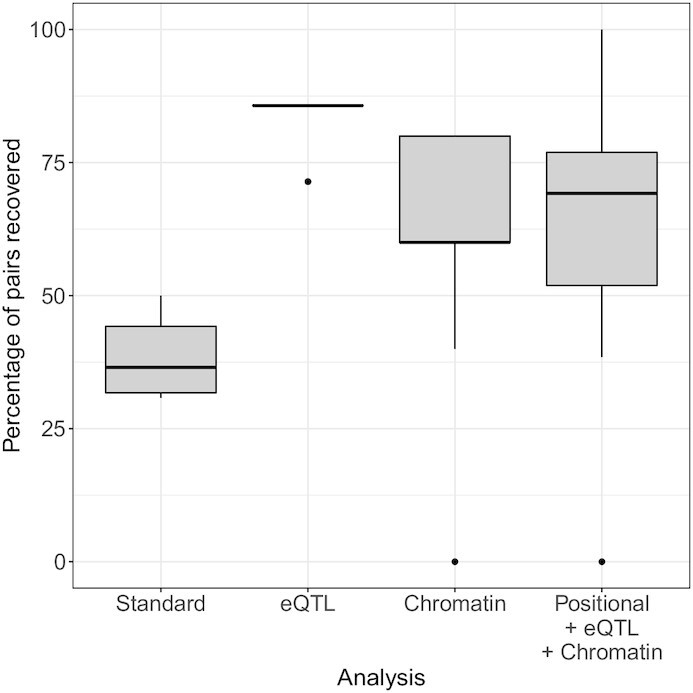
: Gene pairs produced within each mapping across 10 repetitions using 80% of the data, i.e., percentage of gene pairs detected with the entire dataset that are recovered in the 10 subsets with 80% of the individuals. Each box shows the median percentage of gene pairs (the bold line in the middle), with the second and the third quartiles below and above it, respectively; the whiskers represent the first and fourth quartiles; the outliers are indicated separately.

Figure 6 shows that pipelines including biological knowledge recover >60% of the gene pairs detected with the entire cohort, on average (83% for *eQTL*, 60% for *Chromatin* mapping, and 64% for *Positional +eQTL+ Chromatin*), whereas without this knowledge (*Standard*), we recover <40% of the pairs. Hence, the *Standard* analysis seems to be the less robust in terms of conservation of gene pairs. This shows that filtering does increase robustness at the gene level.

### Tissue-specific mappings do not recover many new interactions

To analyze the effect of tissue-specific mappings, we ran 3 analyses using exclusively *eQTL* and *Chromatin* mappings obtained from relevant tissue types and a combination of these *eQTL* and *Chromatin* mappings with the Positional one. Specifically, we used mappings obtained from organs and tissues from the nervous and digestive systems (Supplementary Table S3). While the tissue-specific *Chromatin* analysis did not produce any significant gene pair, the tissue-specific *eQTL* and *Positional +eQTL+ Chromatin* analyses produced, respectively, 4 and 3 significant gene pairs (Supplementary Fig. S4). Nonetheless, only 1 is novel with regards to the organism-wide analyses: *IL18RAP-IL18R1*.

## Discussion

In this article we proposed a new protocol for epistasis detection, based on a variety of functional filtering strategies, and studied its application to GWAS data for IBD. The protocol included several components to control for type I error, thereby strengthening our belief in the discovered genetic interactions.

A common theme in the interpretation of epistasis results consists in linking the associated variants to an altered gene function. In this article, we considered 3 different such SNP-gene mappings. Notably, the number of SNP-gene correspondences provided by each mapping differed by orders of magnitude. Moreover, the different mappings unevenly described genes; e.g., genes that had the most SNPs mapped by using a *Chromatin* contact map had comparatively few *eQTL* SNPs. To combine these different perspectives of the epistasis process we combined multiple mappings into 1 analysis (*Positional +eQTL+ Chromatin*). This joint analysis allows biologically interesting interactions to be detected, such as an SNP in a distal enhancer of a gene (captured by the *Chromatin* mapping) interacting with another gene’s *eQTL*. For the most part, this complementary approach improved the analysis, by recovering most of the interactions significant in the analyses that used 1 mapping at a time. Importantly, our results display the benefits of going beyond 1 single SNP-gene mapping (often, genomic position) to interpret epistasis results. To our surprise, tissue-specific analyses using exclusively *eQTL* and *Chromatin* mappings from tissues related to IBD resulted in less significant gene pairs. Despite this setback, we believe that more targeted analyses (e.g., using only interactions from open *Chromatin* in relevant cell types) might lead to novel discoveries.

Restricting the tested interactions to functionally plausible pairs of genes and SNPs joins 2 faces of epistasis: searching for statistical epistasis, yet exclusively on plausible candidates for biological epistasis. This has several advantages. First, a more targeted input dataset reduces the number of tests and, in consequence, the multiple testing burden. In contrast, the high dimensionality of GWAIS data requires a much more stringent multiple testing correction and limits the detection of epistasis with low effect sizes. Adopting one of the proposed analyses may reduce the number of SNP interactions to test by more than half (Fig. 1). Yet, the *Standard* analysis, which does not use Biofilter, produced the most significant gene models. Second, the proposed protocol addresses the robustness issues widespread in GWAIS by producing results that are consistent at the gene and pathway levels (Fig. 5). Indeed, we observed an increased analytic robustness when using Biofilter gene models, in line with previous reports [32]. In particular, the *eQTL* and *Chromatin* mappings increased said robustness. Third, restricting the search for epistasis to biologically plausible interactions yields results that are biologically interpretable and strikingly different from the ones obtained without using functional filtering (Fig. 2). Not surprisingly, different mappings also provided very different interaction signals and give resolution of information on different genes. In particular, we corroborated that the significant gene models from different functional filters were relevant to the biology of IBD. This was especially true for the *Chromatin* analysis (but also the *eQTL* analysis), giving rise to interactions with seemingly meaningful biological underpinnings, and stressing the relevance of regulatory variants in susceptibility to IBD. In contrast, the *Standard* analysis detected multiple interactions that were hard to interpret. For instance, several interactions involved RNA genes of unknown function (e.g., *LOC101927272* or *LINC02178*).

Remarkably, while the *Standard* analysis produced rich results, the Positional analysis did not lead to any significant SNP model. They both use genomic position to map SNPs to genes, but Positional is restricted to gene models in Biofilter. We note that the Positional analysis does not coincide with how Biofilter is typically used on GWAS data for epistasis detection. The latter involves pooling all SNPs that are mapped to genes that occur in Biofilter proposed gene interaction models, and subsequently exhaustively screening those SNPs for pairwise interactions. These pairs may also involve gene pairs that were not highlighted by Biofilter, in contrast to our Positional analysis. We evaluated the effect of Biofilter on the final results. No significant SNP interactions were detected in the Positional analysis. In the analysis without biofiltering (dataset reduced to mappable SNPs using genomic proximity, but not reduced to Biofilter gene pairs), 62 pairs were significant. Also, of the 86 SNP interactions that passed the experimental threshold in the *Standard* analysis (dataset not reduced to mappable using genomic proximity, nor Biofilter gene pairs), only 57 are mappable to gene interactions using genomic proximity. Hence, 66% of significant SNP pairs are mappable via genomic proximity in the *Standard* analysis.

An important component of our protocol is the conversion of SNP-based tests to gene-based tests. The most popular approach consists in aggregating SNP-level *P*-values into gene-level statistics, which can be done in different ways (see Ma et al. [33] for some early examples, and Vsevolozhskaya et al. [34] for recent developments). Here, we developed a generic approach that exploits a permutation strategy to define a *P*-value cut-off for SNP interactions, at a family-wise error rate (FWER) of 5%, and then we followed the original implementation of the ATPM to accommodate several truncation thresholds at once [19] while taking permutations instead of bootstrap as in Yu et al. [35]. The 2 algorithms are similar, but we favored the TPM over the rank truncated product method of Yu et al. [35] that employs the product of the *L* most significant *P*-values, because the TPM only requires *P*-values smaller than a specified threshold, which is in line with the output of PLINK epistasis detection and saves storage space. Following both protocols and the recommendation of Becker and Knapp [36] we included measures derived from observed data in computing statistics under the null.

Remarkably, our proposed procedure keeps type I error under control, without additional corrections for multiple testing at the gene model. We hypothesize that this stems from 2 reasons. First, we apply a stringent correction for multiple testing at the SNP level. Second, when moving from SNP model significance to gene model significance, the ATPM only considers gene models that map to ≥1 significant SNP model. However, alternative strategies could have been considered, e.g., not restricting ourselves to significant SNP models, hence conducting ATPM on all gene models. This could have led to increased discovery, in cases where the SNP models mapped to a gene tend to be low, albeit non-significant. However, it may also lead to an increased type I error. Accounting for that would require a multiple-test correction at the gene level. In turn, such correction would be difficult because the dependency between the tests is unknown. Additionally, in common multiple-test correction procedures this would require a much higher number of permutations to obtain the necessary numerical precision.

How best to perform a pathway analysis of epistasis results is understudied. Often, all genes belonging to any significant gene pair are simply pooled together into a joint enrichment analysis. This approach discards the gene-gene interaction information that was, indeed, the object of analysis. Hence, in our procedure we adapted the “Network neighborhood search” protocol from Yip et al. [37], which considers the topology of the network using the shortest paths between the studied genes. It should be noted that we only used the topology to derive a neighborhood for each significant gene pair; then, we discarded the edge information. Yet, there are several directions for improvement. One is to exploit the topology of the epistasis network beyond the creation of a neighborhood. Another one is to take into account the gene size (or the number of SNPs per gene), e.g., by performing a weighted version of the statistical test. Jia et al. [38] suggested a method for gene set enrichment analysis of GWAS data, adjusting the gene length bias or the number of SNPs per gene. In our data, we did observe a link between the significance of the gene models and the number of SNPs mapped to the gene. For instance, in the *eQTL* analysis, the only one producing significant pathways, the median number of SNPs per gene is 385 among genes in significant pairs, versus 3 SNPs per gene genome-wide.

Several protocol changes may affect final results. As reported elsewhere [32], these changes or choices include the modeling framework (parametric, non-parametric, semi-parametric) and encoding of the genetic markers, as well as LD handling. With regards to the first one, we used a linear regression. Because the IIBDGC dataset is case-control, a more natural choice would have been logistic regression, including the 7 main PCs to account for population structure. However the tool of choice (PLINK) did not allow the inclusion of covariates. To work around this, we took the binary phenotype as a continuous variable, regressed out the 7 largest PCs, and fitted a linear regression to this adjusted phenotype. Although this approach works well in practice, it is suboptimal, and more flexible frameworks might account for the population structure more accurately. With regards to the encoding, we used an additive encoding scheme (0, 1, 2 indicating the number of copies of the minor SNP allele), a popular choice in part because of its computational efficiency. However, this encoding scheme has been reported to tend to increase false-positive results (e.g., [39]). This observation is based on type I error studies with data generated under the null hypothesis of no pairwise genetic interactions but in the presence of main effects (see, e.g., [40]). Here, we investigated the type I error control of our protocols under a general null hypothesis of no genetic associations with the trait (no interactions and no main effects) and established adequate control. As a consequence, this does not guarantee that our generated SNP interaction results were not overly optimistic. To this end, we adjusted SNP-level epistasis *P*-values for main effects as comprised in a polygenic risk score. Not only does such a post-analysis adjustment via conditional regression reduce over-optimism due to inadequate control for lower-order effects, thus addressing phantom epistasis [24], but it may also occasionally highlight the masking of SNP interactions (as was shown in Fig. 4B—*eQTL*). More work is needed to investigate the effect on gene-level interaction results, derived accordingly. For convenience, we used the regression framework to identify SNP interactions and relied on earlier recommendations regarding LD handling [41].

Our protocols are built on output from Biofilter, which can be presented as a co-functional gene network. One of the motivations was its proven ability to highlight meaningful interactions in a narrower alternative hypothesis space, at the expense of leaving parts of the interaction search space unexplored. The database that Biofilter built contained 37,266 interactions. This is notably smaller than other gene interaction databases, such as HINT [42] (173,797 interactions) or STRING [43] (11,759,455 interactions). Testing gene interactions with other (combinations of) biological interaction networks was beyond the scope of this article. Furthermore, Biofilter analysis or exhaustive screening may lead to non-overlapping results. An example within a regression context is given in [32].

## Potential Implications

In this study we presented a protocol to enhance the interpretation of epistasis screening from GWAS. It includes gene-level epistasis discoveries with type I error under control, as well as a network-guided pathway analysis. Moreover, it improves the robustness of the results. While SNP pairs from a GWAIS study rarely replicate in other cohorts and arrays, results at the gene and pathway level are more likely to be reproducible. This can be achieved directly by applying the proposed protocol, or by testing SNP models in a cohort obtained from the gene pairs and pathways significant in other studies, via the SNP-gene mapping of interest. Aggregating SNP-level results into gene-level epistasis is challenging but allows the inclusion of information from biological interaction databases. On the basis of that, we conducted multiple analyses that use different sources of prior biological knowledge about SNP-to-gene relationships and gene interaction models, as well as rigorous statistical approaches to assess significance. Each of them offers a different, albeit complementary view of the disease, which leads to additional insights.

Their application to GWAS data for IBS highlighted the potential of our strategy, including network-guided pathway analysis, as it recovered known aspects of IBD while capturing relevant and previously unreported features of its genetic architecture. These strategies will contribute to identify gene-level interactions from SNP data for complex diseases, and to enhance our belief in these findings.

## Methods

### Gene interaction detection procedure

**Figure 7:**
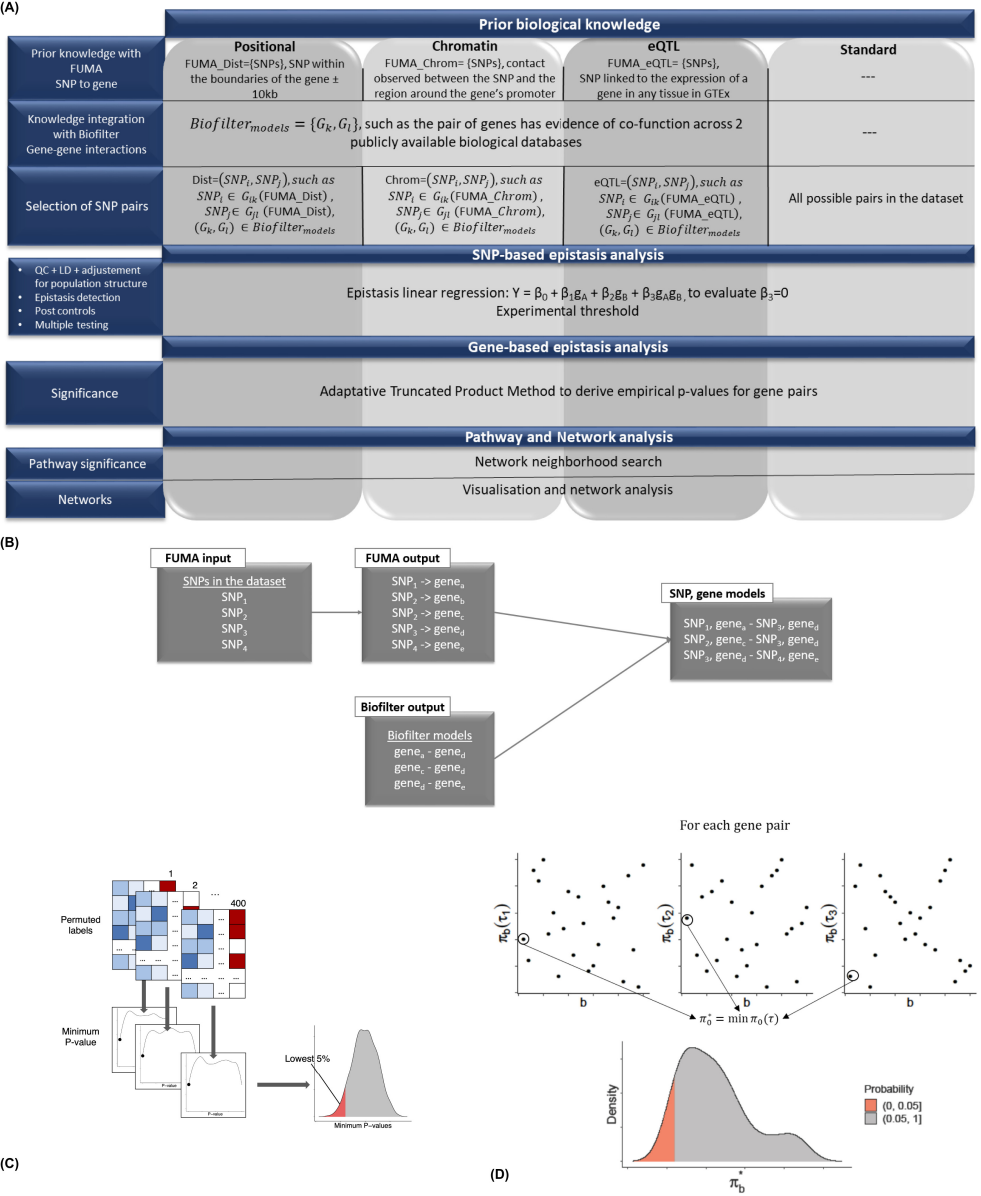
: (A) Overview of the investigated gene-gene interaction detection protocols, described in section “*Gene interaction detection procedure*.” (B) Summary of the procedure to obtain SNP and gene models using FUMA and Biofilter, described in section “*Co-function gene and SNP networks*.” (C) Permutation procedure to obtain the SNP model *P*-value threshold, described in section “*SNP-level epistasis detection and multiple testing correction*.” (D) Overview of the adaptive truncated product methodology, described in section “*From SNP-level to gene-level epistasis*.”

As we describe in more detail below, we applied different functional filters to the available data. These filters use plausible interactions between genes, and 3 different ways of mapping SNPs to those genes, and hence, to these interactions. These 3 mappings exploit different degrees of biological knowledge to map SNPs to genes, referred to as Positional, *eQTL*, and *Chromatin*. For each of the 3 SNP-to-gene mappings, we only analyzed the pairs of SNPs corresponding to a gene pair with prior evidence for interaction. Across this article, we compared our findings in these scenarios to a *Standard* scenario. In this case, we exhaustively search for epistasis between all 38,225 SNPs that passed quality control (Table 4). We mapped the resulting significant SNP interactions to potential gene interactions using the positional mapping. An overview of the entire pipeline is presented in Fig. 7.

**Table 4:** Properties of the different SNP-gene mappings and the filtered datasets. We show the empirical threshold of significance obtained through permutation, and the corresponding Bonferroni threshold for comparison

Analysis	Mappings	SNP models (SNPs)	Empirical	Bonferroni^1^
*Standard*		7.3 × 10^8^ (38,225)	1.1 × 10^−10^	6.9 × 10^−11^
*Positional*	1.7 × 10^5^	3.0 × 10^5^ (16,417)	1.6 × 10^−7^	1.7 × 10^−7^
*Chromatin*	2.4 × 10^6^	6.0 × 10^6^ (30,146)	1.0 × 10^−8^	8.3 × 10^−9^
*eQTL*	4.1 × 10^5^	1.2 × 10^6^ (16,652)	6.2 × 10^−8^	4.0 × 10^−8^
*eQTL + Chromatin*	2.7 × 10^6^	9.0 × 10^6^ (33,419)	6.5 × 10^−9^	5.6 × 10^−9^
*Positional + eQTL*	5.3 × 10^5^	1.7 × 10^6^ (23,642)	3.9 × 10^−8^	2.9 × 10^−8^
*Positional + Chromatin*	2.5 × 10^6^	7 × 10^6^ (33,195)	8.2 × 10^−9^	7.1 × 10^−9^
*Positional + eQTL + Chromatin*	2.8 × 10^6^	9.6 × 10^6^ (34,548)	5.0 × 10^−9^	5.2 × 10^−9^

^1^ Bonferroni threshold based on the number of possible SNP pairs in the analysis.

#### From gene models to SNP models

Although the unit of analysis in GWAIS is the SNP, biological interactions are often characterized at the gene level. Hence, we mapped all SNPs in the dataset to genes using FUMA [44], a post-GWAS annotation tool. We created an artificial input where every SNP is significant in order to perform such mapping on all the SNPs. We performed 3 SNP-gene mappings using FUMA’s SNP2GENE: positional, *eQTL*, and 3D *Chromatin* interaction (Table 4). In the Positional mapping, we mapped an SNP to a gene when the genomic coordinates of the former were within the boundaries of the latter ±10 kb. The *eQTL* mapping uses *eQTL*s obtained from GTEx [45]. We mapped an *eQTL* SNP to its target gene when the association *P*-value was significant in any tissue (FDR < 0.05). Last, in the *Chromatin* mapping, we mapped an SNP to a gene when a contact had been observed between the former and the region around the latter’s promoter in the 3D structure of the genome (250 bp upstream and 500 bp downstream from the transcription start site) in any of the Hi-C datasets included in FUMA (FDR < 10^−6^). This mapping might contain new, undiscovered, regulatory variants that, as for SNPs obtained through *eQTL* mapping, regulate the expression of a gene.

#### Co-function gene and SNP networks

We used Biofilter 2.4 [18] to obtain candidate gene pairs to investigate for epistasis evidence. Biofilter generates pairs of genes susceptible to interact (“gene models”) with evidence of co-function across multiple publicly available biological databases. It includes genomic locations of SNPs and genes, as well as known relationships among genes and proteins such as interaction pairs, pathways, and ontological categories, but does not use trait information. As per Biofilter’s default, we used gene models supported by evidence in ≥2 databases. In addition, we removed self-interactions because detection of within-gene epistasis requires special considerations and is beyond the scope of this article.

Given this set of gene models, and 3 different ways of obtaining SNP models from it, we removed all the SNPs that did not participate in any SNP model. Subsequently, we created 8 datasets. In 1 dataset no filter was applied (*Standard* analysis), i.e., neither Biofiltering nor any SNP-to-gene mapping. Hence, the original SNP set was used. We also created 1 dataset exclusively for each SNP-to-gene mapping (Positional, *eQTL*, and *Chromatin*). Last, we created 4 datasets using joint mappings: 1 with all the mappings (*Positional +eQTL+ Chromatin*) and 3 with only 2 of them (*eQTL+ Chromatin, Positional +eQTL*, and *Positional + Chromatin*).

We discarded SNP models involving rare variants (minor allele frequency <5%) or in Hardy–Weinberg equilibrium (*P*-value < 0.001). Regardless, all risk SNPs described in Liu et al. [46] were included, even when the aforementioned epistasis quality control criteria did not hold up. Then, when the 2 SNPs of an SNP model were located in the HLA region, we discarded the pair because it is difficult to differentiate between main and non-additive effects in this region [47]. Last, we discarded models where the SNPs were in linkage equilibrium (*r*^2^  > 0.75), as motivated in Gusareva and Van Steen [41].

#### SNP-level epistasis detection and multiple testing correction

We used PLINK 1.9 to detect epistasis through a linear regression on the population structure adjusted phenotypes with the option −epistasis:

(1) \begin{eqnarray*}
Y = \beta _0 + \beta _1 g_A + \beta _2 g_B + \beta _3 g_A g_B, \end{eqnarray*}

where *g_A_* and *g_B_* are the genotypes under additive encoding for SNPs A and B, respectively, *Y* is the adjusted phenotype, and β_0_, β_1_, β_2_, and β_3_ are the regression coefficients. PLINK performs a statistical test to evaluate whether β_3_ ≠ 0. It only returns SNP pairs with a *P*-value lower than a specified threshold. We used the default 0.0001. Only SNP models were considered, apart from the *Standard* approach.

To correctly account for multiple testing, the *P*-value threshold of significance had to be dataset-dependent because the number of tested SNP pairs changed from dataset to dataset. We obtained these thresholds through permutations as in Hemani et al. [48] (Fig. 7). More specifically, for each dataset, we permuted the phenotypes 400 times and fitted the aforementioned regression-based association model. This produced a null distribution of the extreme *P*-values for this number of tests given the LD structure in the data. For each dataset, we took the most extreme *P*-value from each of the 400 permutations and set the threshold for 5% FWER to be the 5% percentile of these most extreme *P*-values. Posterior experiments showed that using a higher number of permutations, 1,000, barely changed the empirical threshold (data not shown). Hence, 400 was enough permutations to obtain an adequate threshold.

#### From SNP-level to gene-level epistasis

Our next step was to use significant SNP interactions to identify significant gene interactions, which requires combining the *P*-values of all SNP pairs mapped to the same gene pair. Suppose that SNP interaction tests have been conducted for *N* individual hypotheses *H*_0*i*_, *i* = 1, 2, …, *N*, e.g., *N* SNP models mapped to the same gene model. We tested the joint null hypothesis $H_0=\bigcap ^n_{i=1}H_{0i}$ at significance level α versus the combined alternative hypothesis *H*_1_: ≥1 of *H*_0*i*_ is false. To do so, we considered all SNP pairs mapped to the same gene pair as a set of tests with the same global null hypothesis, and applied the ATPM [19] (Fig. 7).

ATPM is an adaptive variant of the truncated product method (TPM) of Zaykin et al. [49], which uses as a statistic the product of the *P*-values smaller than some pre-specified threshold (here, significant SNP interactions) tests. More specifically, given a truncation point τ and a number *N* of significant SNP interactions, this test statistic is given as $W(\tau )=\prod _{i=1}^{N} p_i^{I(p_{i}\le \tau )}$, where *I*( · ) is the indicator function. TPM is interesting in our context because it does not require *P*-values for all SNP pairs but only for the most strongly associated ones.

The distribution of *W*(τ) under the null hypothesis is unknown when the individual tests are not independent, which is clearly the case here, but an empirical *P*-value $\hat{\pi }(\tau )$ can be estimated through permutations. Because the choice of τ is arbitrary, the adaptive version of TPM (ATPM) explores several values of τ and selects $\hat{\pi }^{*} = \min _\tau \hat{\pi }(\tau )$. The distribution of $\hat{\pi }^{*}$ under the null hypothesis can again be determined through permutations [50].

In our procedure, which is detailed below for a given gene pair, we used *B* = 999 permutations and τ ∈ {0.001, 0.01, 0.05}. Remarkably, and following the suggestion of Becker and Knapp [36], the null distribution includes both the statistic from the observed dataset, and from the 999 permutations.

1. For each SNP model *i* = 1, …, *N* mapped to the gene pair, compute its *P*-values *p_i, b_* in the original dataset (*b* = 0) and for each of the *B* = 999 permutations (*b* = 1, …, *B*).
2. For each value of τ and *b*, compute the test statistic *W*(τ).
3. For each value of τ and *b*, estimate the *P*-value: ${\pi }_{b}(\tau )=\left[\sum _{l=0}^B I(W_b(\tau ) \ge W_l(\tau ))\right]/(B+1)$.
4. For each value of *b*, compute ${\pi }^{*}_{b} =\mathrm{ min}_{\tau }{\pi }_{b}(\tau )$.
5. Estimate the *P*-value of the gene model as $P_0 = \left[\sum _{l=0}^B I(\pi ^{*}_0 \ge \pi ^{*}_l)\right]/(B+1)$.
6. Reject the global null hypothesis if *P*_0_ ≤ α = 0.05.

### Studying the impact of confounding main effects

The SNPs from some detected interactions were near SNPs with main effects. To assess the impact on the results, we studied the difference between β_3_ in Eq. 1 and in the following model:

$$Y = \beta _0 + \beta _1 g_A + \beta _2 g_B + \beta _3 g_A g_B + \beta _4 \text{PRS}.$$

PRS is the polygenic risk score (PRS) computed for the sample. We expect the PRS to capture the variance explained by all main effects.

We computed the PRS with PRSice-2 [51], using the default options. Because it requires GWAS summary statistics, we used PLINK −assoc to compute the association of each SNP in the original dataset (130,071 SNPs and 66,280 individuals, with the trait adjusted for PCs). Because the adjusted phenotype is quantitative, PLINK computes the linear regression coefficients and assesses their significance using the Wald test. PRSice performs clumping to remove SNPs that are in LD with each other. The *r*^2^ values computed by PRSice are based on maximum likelihood haplotype frequency estimates. From the 130,071 initial variants, 28,389 variants remained after clumping (−clump−kb 250kb, −clump−p 1, −clump−r2 0.1). We used the average effect size method to calculate the PRS, with high-resolution scoring.

### Pathway analysis

A pathway enrichment analysis on the neighborhood of a significant gene model can inform about the broader framework in which gene epistasis occurs. To define such neighborhoods, we adapted the network neighborhood search protocol from Yip et al. [37]. We computed the neighborhood of 2 genes as the list of all genes that (1) participate in any of the shortest paths between the 2 studied genes in the Biofilter network, once the direct link between them is removed; and (2) are also involved in a significant interaction with ≥1 other gene on these paths. We restricted our attention to neighborhoods containing ≥3 genes, including the 2 from the gene model under consideration. For each of these, we conducted a gene set enrichment analysis in all human gene sets from the Molecular Signature Database (MSigDB version 7) [52,53]. We performed the enrichment analysis using a hypergeometric test, which compares the obtained overlap between 2 sets to the expected overlap from taking equally sized random sets from the universe of genes. We favored the hypergeometric test over the χ^2^ test used in Yip et al. [37] because the sample sizes of the neighborhoods were small and because χ^2^ is an approximation whereas the hypergeometric test is an exact test. The universe set was analysis dependent. For the *Standard* analysis, it contained all the genes in an annotated pathway that can be mapped via genomic proximity to an SNP of the dataset. For the other analyses, it contained all the genes in an annotated pathway that are present in at least one Biofilter gene model, and that can be mapped via the corresponding SNP to gene mapping to an SNP in the dataset. Finally, pathways were said to be significant when the corresponding test *P*-value was lower than the Bonferroni threshold ($0.05 / (\text{# pathways} \times \text{# tested gene neighborhoods})$), with “pathways” corresponding to pathways containing ≥1 gene of the neighborhood.

## Availability of Source Code and Requirements

- Project name: network_epistasis.nf
- Project home page: https://github.com/hclimente/gwas-tools
- Operating system(s): Platform independent
- Programming language: nextflow
- Other requirements: Bash, nextflow, PLINK 1.9 and R 4.0 or higher, with the following packages: clusterProfiler, data.table, igraph, msigdbr, snpStats, tidyverse
- License: GNU GPL v3.0

We also made this pipeline available in bio.tools (id: network_epistasis) and SciCruch (network_epistasis, RRID: SCR_021794) databases.

Additionally, the code necessary to reproduce this article’s results and analyses is available on GitHub at https://github.com/DianeDuroux/BiologicalEpistasis.

## Data Availability

The dataset underlying this article is available upon request from the International Inflammatory Bowel Disease Genetics Consortium (https://www.ibdgenetics.org/). GWAS summary statistics are publicly available in this article's accompanying repository at https://github.com/DianeDuroux/BiologicalEpistasis. Snapshots of the code are available in the *GigaScience* GigaDB repository [54].

## Additional Files

**Supplementary Figure S1**: To choose the best way of computing residuals to obtain the phenotype adjusted for population structure, we randomly extracted 5 SNPs in the dataset (rs12488468, rs1005678, rs11714286, rs2267844, rs11720964) and compared the associated outputs of epistasis detection. First, we computed the different residuals: we ran a logistic regression model with binary phenotypes as the response variable and 7 PCs as independent variables. We derived 3 vectors of adjusted phenotypes from the response, working, and Pearson residuals. Then, we looked for statistical epistasis: we computed linear models using the different residuals as the response variable and 2 SNPs and their interactions as independent variables. Finally, we performed logistic regressions with the binary phenotype as the dependent variable, and 2 SNPs and their interaction as explanatory variables, in addition to 7 PCs as covariates. We aimed at identifying the residuals leading to *P*-values as close as possible to the *P*-values from the logistic regression. *P*-values obtained with response residuals as phenotypes are the closest to the ones obtained with the logistic regression and are therefore selected as adjusted phenotypes in our analysis.

**Supplementary Figure S2**: Epistasis networks built from the significant SNP models of the different analysis.

**Supplementary Figure S3**: Alternative visualization of the gene-networks presented in Fig. 2. Genes associated with IBD in DisGeNET have a diamond shape.

**Supplementary Figure S4**: Epistasis networks built from the significant SNP models and gene models of the tissue-specific *eQTL* and *Positional +eQTL+ Chromatin* analyses. Genes associated with IBD in DisGeNET are indicated in pink.

**SupplementaryTable S1**: The 16 pathways enriched in the *eQTL* analysis. They are obtained from 1 gene neighborhood (*HYAL1, HYAL3, SPAM1*).

**Supplementary Table S2**: The 71 pathways enriched in the *Positional +eQTL+ Chromatin* analysis. They are obtained from 2 gene neighborhoods (*HYAL3, HYAL1, HYAL2* and *PLA2G2E, PLA2G5, PLA2G2C*).

**Supplementary Table S3**: Tissue-specific *eQTL* and *Chromatin* analyses. For *eQTL*, we took associations from the following tissues: colon sigmoid, colon transverse, esophagus gastroesophageal junction, esophagus mucosa, esophagus muscularis, pancreas, small intestine terminal ileum, stomach, whole blood, aveALL (average gene expression among 10 brain regions), brain anterior cingulate cortex BA24, brain caudate basal ganglia, brain cerebellar hemisphere, brain cerebellum, brain cortex, brain frontal cortex BA9, brain hippocampus, brain hypothalamus, brain nucleus accumbens basal ganglia, brain putamen basal ganglia, and brain amygdala. For *Chromatin*, we took contacts measured in pancreas, small bowel, dorsolateral prefrontal cortex, and hippocampus.

giab093_GIGA-D-21-00039_Original_Submission

giab093_GIGA-D-21-00039_Revision_1

giab093_GIGA-D-21-00039_Revision_2

giab093_GIGA-D-21-00039_Revision_3

giab093_Response_to_Reviewer_Comments_Revision_1

giab093_Reviewer_1_Report_Original_Submission Shing Wan Choi -- 3/24/2021 Reviewed

giab093_Reviewer_2_Report_Original_Submission Elisabetta Manduchi -- 7/13/2021 Reviewed

giab093_Reviewer_2_Report_Revision_1 Elisabetta Manduchi -- 10/24/2021 Reviewed

giab093_Supplemental_File

## Abbreviations

ATPM: adaptive truncated product method; bp: base pairs; *eQTL*: expression quantitative trait loci; FDR: false discovery rate; FWER: family-wise error rate; GWAS: genome-wide association study; GWAIS: genome-wide association interaction study; IBD: inflammatory bowel disease; kb: kilobase pairs; LD: linkage disequilibrium; SNP: single-nucleotide polymorphism; PC: principal component; TPM: truncated product method.

## Competing Interests

The authors declare that they have no competing interests.

## Funding

This project has received funding from the European Union’s Horizon 2020 research and innovation programme under the Marie Sklodowska-Curie grant agreements No. 666003 and 813533. H.C.G. acknowledges funding from the RIKEN Special Postdoctoral Researcher Program. C-A.A. acknowledges funding from Agence Nationale de la Recherche (ANR-18-CE45-0021-01). Computational resources have been provided by the Consortium des Équipements de Calcul Intensif (CÉCI), funded by the Fonds de la Recherche Scientifique de Belgique (F.R.S.-FNRS) under Grant No. 2.5020.11 and by the Walloon Region. K.V.S. acknowledges opportunities and funding provided by WELBIO (Walloon Excellence in Life sciences and BIOtechnology).

## Authors’ Contributions

Conceptualization: All authors; data curation: D.D., H.C.G.; formal analysis: D.D., H.C.G.; funding acquisition: C-A.A., K.V.S.; investigation: D.D., H.C.G.; methodology: D.D., K.V.S.; project administration: C-A.A., K.V.S.; resources: C-A.A., K.V.S.; software: D.D., H.C.G.; supervision: C-A.A., K.V.S.; validation: D.D., H.C.G.; visualization: D.D., H.C.G.; writing—original draft: D.D., H.C.G.; writing—review & editing: all authors.
