# Supplementary material for: Interpretable network-guided epistasis detection
Source: Gigascience. 2022 Feb 4;11:giab093. doi: 10.1093/gigascience/giab093 (PMC8848319; doi:10.1093/gigascience/giab093)
Supplement: giab093_GIGA-D-21-00039_Original_Submission [file giab093_giga-d-21-00039_original_submission.pdf]

GigaScience

Interpretable network-guided epistasis detection

--Manuscript Draft--

**Manuscript Number:**	GIGA-D-21-00039
**Full Title:**	Interpretable network-guided epistasis detection
**Article Type:**	Research
**Funding Information:**	H2020 Marie Skłodowska-Curie Actions () (666003) — Dr Chloé-Agathe Azencott<br>H2020 Marie Skłodowska-Curie Actions (813533) — Dr Kristel Van Steen<br>Agence Nationale de la Recherche (FR) (ANR-18-CE45-0021-01) — Dr Chloé-Agathe Azencott<br>WELBIO — Dr Kristel Van Steen
**Abstract:**	Detecting epistatic interactions at the gene level is essential to understanding the biological mechanisms of complex diseases. Unfortunately, genome-wide interaction association studies (GWAIS) involve many statistical challenges that make such detection hard. We propose a multi-step protocol for epistasis detection along the edges of a gene-gene co-function network. Such an approach reduces the number of tests performed and provides interpretable interactions, while keeping type I error controlled. Yet, mapping gene-interactions into testable SNP-interaction hypotheses, as well as computing gene pair association scores from SNP pair ones, is not trivial. Here we compare three SNP-gene mappings (positional overlap, eQTL and proximity in 3D structure) and used the adaptive truncated product method to compute gene pair scores. This method is non-parametric, does not require a known null distribution, and is fast to compute. We apply multiple variants of this protocol to a GWAS inflammatory bowel disease (IBD) dataset. Different configurations produced different results, highlighting that various mechanisms are implicated in IBD, while at the same time, results overlapped with known disease biology. Importantly, the proposed pipeline also differs from a conventional approach were no network is used, showing the potential for additional discoveries when prior biological knowledge is incorporated into epistasis detection.
**Corresponding Author:**	Héctor Climente-González<br>RIKEN<br>Tokyo, JAPAN
**Corresponding Author Secondary Information:**	
**Corresponding Author's Institution:**	RIKEN
**Corresponding Author's Secondary Institution:**	
**First Author:**	Diane Duroux
**First Author Secondary Information:**	
**Order of Authors:**	Diane Duroux<br>Héctor Climente-González<br>Chloé-Agathe Azencott<br>Kristel Van Steen
**Order of Authors Secondary Information:**	
**Additional Information:**	
**Question**	**Response**
Are you submitting this manuscript to a	No

Powered by Editorial Manager® and ProduXion Manager® from Aries Systems Corporation

special series or article collection?	
**Experimental design and statistics**<br><br>Full details of the experimental design and statistical methods used should be given in the Methods section, as detailed in our Minimum Standards Reporting Checklist. Information essential to interpreting the data presented should be made available in the figure legends.<br><br>Have you included all the information requested in your manuscript?	Yes
**Resources**<br><br>A description of all resources used, including antibodies, cell lines, animals and software tools, with enough information to allow them to be uniquely identified, should be included in the Methods section. Authors are strongly encouraged to cite Research Resource Identifiers (RRIDs) for antibodies, model organisms and tools, where possible.<br><br>Have you included the information requested as detailed in our Minimum Standards Reporting Checklist?	Yes
**Availability of data and materials**<br><br>All datasets and code on which the conclusions of the paper rely must be either included in your submission or deposited in publicly available repositories (where available and ethically appropriate), referencing such data using a unique identifier in the references and in the "Availability of Data and Materials" section of your manuscript.<br><br>Have you have met the above requirement as detailed in our Minimum Standards Reporting Checklist?	Yes

Powered by Editorial Manager® and ProduXion Manager® from Aries Systems Corporation

Manuscript

Click here to access/download;Manuscript;Interpretable_network_guided_ep

Interpretable network-guided epistasis detection

Diane Duroux[1¶*], Héctor Climente-González[2,3,4,5,¶], Chloé-Agathe Azencott[4,2,3], Kristel Van Steen[1,6]

[¶]Equally contributing authors;

[*]Corresponding author. Email: diane.duroux@uliege.be (DD);

[1]BIO3 - GIGA-R Medical Genomics, University of Liege, Liege, Belgium;

[2]Institut Curie, PSL Research University, F-75005 Paris, France;

[3]INSERM, U900, F-75005 Paris, France;

[4]MINES ParisTech, PSL Research University, CBIO-Centre for Computational Biology, F-75006 Paris, France;

[5]RIKEN Center for Advanced Intelligence Project (AIP), Tokyo, Japan;

[6]BIO3 - Department of Human Genetics, KU Leuven, Herestraat 49, B-3000 Leuven, Belgium.

Abstract

Detecting epistatic interactions at the gene level is essential to understanding the biological mechanisms of complex diseases. Unfortunately, genome-wide interaction association studies (GWAIS) involve many statistical challenges that make such detection hard. We propose a multi-step protocol for epistasis detection along the edges of a gene-gene co-function network. Such an approach reduces the number of tests performed and provides interpretable interactions, while keeping type I error controlled. Yet, mapping gene-interactions into testable SNP-interaction hypotheses, as well as computing gene pair association scores from SNP pair ones, is not trivial. Here we compare three SNP-gene mappings (positional overlap, eQTL and proximity in 3D structure) and used the adaptive truncated product method to compute gene pair scores. This method is non-parametric, does not require a known null distribution, and is fast to compute. We apply multiple variants of this protocol to a GWAS inflammatory bowel disease (IBD) dataset. Different configurations produced different results, highlighting that various mechanisms are implicated in IBD, while at the same time, results overlapped with known disease biology. Importantly, the proposed pipeline also differs from a conventional approach were no network is used, showing the potential for additional discoveries when prior biological knowledge is incorporated into epistasis detection.

Author summary

In this article, we propose a new epistasis detection protocol that leverages the growing biological knowledge. We focus on genes, rather than SNPs, which facilitate interpretation and are more consistent between populations

or methods. To that end we take known gene-gene interactions, and map them to SNP pairs using functional mappings, based on SNP position, eQTL and chromatin-structure. Then, we search for epistasis exclusively on these SNP pairs. Using only known interactions narrows down the search space and helps identifying biologically relevant epistasis. We convert significant SNP pairs into gene pairs by aggregating significant SNP pairs into gene pairs, whose significance we examined using an ATPM step. Lastly, we examine the network neighborhood of the significant gene pairs to link them to pathways. We illustrate our proposed protocol by applying it to an inflammatory bowel disease (IBD) GWAS dataset. Our proposed protocol recovered genes and interactions previously reported in the literature as relevant to IBD, as well as potentially novel interactions. In contrast, not using functional mappings and known interactions resulted in a very different set of significant gene pairs.

1 Introduction

Genome-wide association studies (GWAS) have identified over 70 000 genetic variants associated with complex traits [1]. Often these variants altogether do not explain the whole variance of a trait. A representative example is inflammatory bowel disease (IBD), like Crohn's disease and ulcerative colitis. Pooled twin studies estimate their heritabilities at 0.75 and 0.67 respectively [2]. Yet, despite large GWAS that identified over 200 IBD-associated loci [3], a low proportion of their variance has been explained [4]. Possible explanations include a large number of common variants with small effects, rare variants with large effects not covered in GWAS, unaccounted gene-environment interactions, and genetic interactions [5]. In this article we explore the latter, called epistasis, which has been linked to IBD in the past [6, 7, 8, 9, 10, 11]. Often, two types of epistasis are described: biological and statistical epistasis [12]. Broadly described, biological epistasis refers to a physical interaction between two biomolecules that has an impact on the phenotype. Statistical epistasis refers to departures from population-level linear models describing relationships between predictive factors such as alleles at different genetic loci.

Genome-wide association interaction studies (GWAIS) focus on the detection of statistical epistasis. To date, these studies have produced few replicable, functional conclusions, and specific gene-gene interactions have rarely been identified. This may be due to the small effects sizes of the interactions, the low statistical power, or the absence of a widely accepted GWAIS protocol. Even in the absence of statistical challenges, GWAIS are usually conducted on Single Nucleotide Polymorphisms (SNPs), with SNP-interactions often lacking a straightforward functional interpretation. Moving from SNP- to gene-level tests, which jointly consider all the SNPs mapped to the same gene, might address both shortcomings. First, aggregating SNP pair statistics into gene pair statistics is likely to increase the statistical power when dealing with complex diseases [13]. Second, converting statistical findings into biological hypotheses [14], may facilitate their functional interpretability [15].

To both reduce the number of tests and improve the interpretablity of significant SNP interactions, some authors propose examining only pairs of SNPs likely to be functionally related [16]. Such approaches use prior biological knowledge, for instance, of SNPs involved in genes that establish a protein-protein interaction [17]. Yet, limiting studies to one particular kind of gene-gene interaction might be reductive. To tackle that

issue, Pendergrass et al. [18] developed Biofilter, a gene-gene co-function network, which aggregates multiple databases. Additionally, such approaches often require as well a proper mapping of SNP to genes.

In this article, we propose guiding statistical epistasis using plausible biological epistasis. Taking exclusively interactions reported from at least 2 different sources in Biofilter, we compile a subset of gene-gene interactions that are biologically plausible. Then, we exclusively search for those interactions in a GWAIS dataset, reducing the multiple test burden and improving the interpretability. We investigate different ways of mapping SNPs to genes and use the adaptive truncated product method to estimate the association of gene pairs. Network and pathway analyses are used to further assist in the interpretation of epistasis findings. The proposed pipeline is applied to GWAS data from the International IBD Genetic Consortium [3].

2 Results

2.1 Type I error

In this article, we propose a protocol for epistasis detection using a gene co-function network (Section 4.2). Due to its multi-stage nature, type I error needs to be controlled. For that purpose, we performed a permutation analysis based on 1 000 permutations for each of the datasets, permuting the phenotypes and running the entire protocol to detect significant gene interactions (Table 1). This permutation procedure is independent of the one used in the proposed protocol to compute significance thresholds. When at least one significant gene interactions was observed in a permutation, that permutation was considered a false positive (FP). This allowed us to compute the type I error rate as $\frac{\#\ \text{FP}}{1000}$. Type I error was under control in all tested experimental settings, with estimates $\leq 6.6\%$.

Analysis	Average num. of significant interactions under H_0	Type 1 error (%)
Standard	0.05	3.6
Positional	0.04	3.7
eQTL	0.09	4.2
Chromatin	0.07	6.6
eQTL + Chromatin	0.05	4.8
Positional + eQTL + Chromatin	0.04	3.6

Table 1: Type I error of the protocol presented in Section 4.2, estimated over 1 000 random permutations, as explained in Section 2.1.

2.2 SNP to gene mapping: Chromatin contacts map more SNPs per gene than other mappings

We obtained gene models from the Biofilter network (Section 4.2.2), and considered three analyses to obtain SNP models from these gene models (Section 4.2.1): *Positional*, *eQTL* and *Chromatin*. *Chromatin* produced the largest number of unique SNP-gene mappings (2 394 590), an order of magnitude more than *eQTL* (411 120) and *Positional* (174 879) (Table 4). The *Chromatin* analysis had on average the largest number of SNPs mapped on to a gene, followed by *eQTL* and *Positional* (Fig 1A). Nonetheless, the number of SNPs mapped to a gene

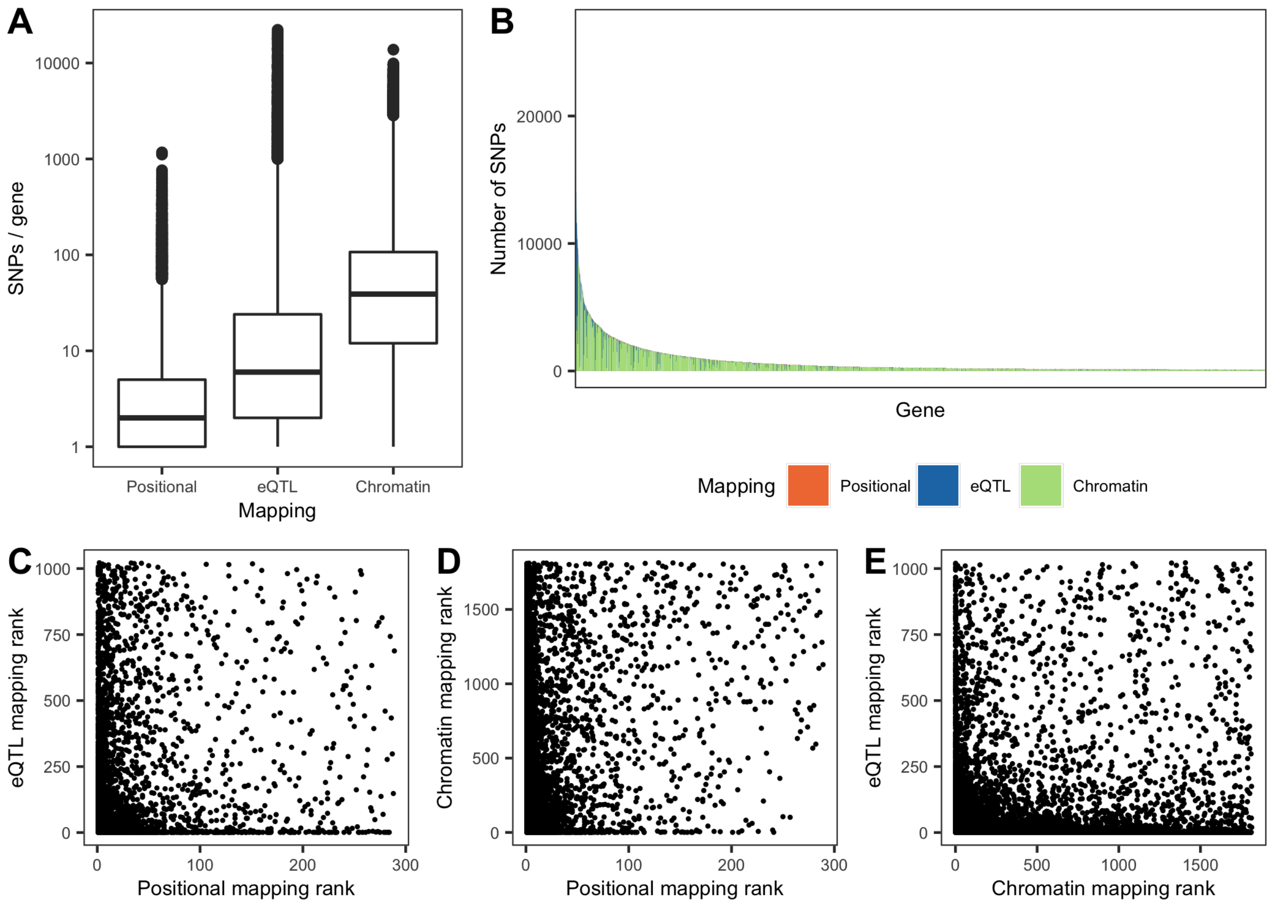

Fig 1: **(A)** Number of SNPs per gene for each of the three mappings described in Section 4.2.1. **(B)** Ranking of genes with most SNPs mapped using any of the mappings, colored by mapping. Only genes with more than 100 SNPs mapped to it are displayed. **(C,D,E)** Comparison between the rank of each gene according to the number of SNPs mapped to it using each mapping.

varied considerably across genes (Fig 1B). In addition, the number of SNPs mapped to a same gene varied considerably across analyses (Fig 1C, D and E): in general, the genes with most SNPs mapped using the *eQTL* mapping had relatively few SNPs mapped in the *Chromatin* mapping, and vice versa.

2.3 The *Positional* analysis does not recover any SNP interaction

Using the aforementioned SNP-gene mappings, and combinations of them (cross-mappings), yielded six datasets in which we analyzed SNP models (Section 4.2.3). The resulting epistatic SNP-SNP networks are described in Table 2 (for visualization, see Supplementary Fig 2). Strikingly, while the *Standard* analysis generated the largest SNP-interaction network (55 nodes/SNPs and 57 edges/interactions), the *eQTL* one was the largest by number of interactions (64). The *Positional* analysis produced no significant interactions at all.

Notably, the significant SNP interactions tended to be located in nearby genomic regions and to overlap with GWAS main effects loci (Fig 2A). To investigate whether main effects could be driving some of the signals, even when in imperfect LD with epistatic SNP pairs (a phenomenon sometimes referred to as "phantom epistasis" [19]), we conducted a linear regression based test as in Section 4.2.3, but including a vector of polygenic risk scores as covariate. The polygenic risk scores were computed with PRSice-2 [20] with the trait adjusted for PCs,

Table 2: Properties of the SNP networks obtained from different datasets (Section 4.2). Nodes are SNPs, which are linked when the SNP model is significant.

Analysis	SNPs	Edges	Components	Avg. degree
Standard	55	57	12	2.07
Positional	0	0	-	-
eQTL	46	64	6	2.78
Chromatin	20	19	5	1.9
eQTL + Chromatin	44	48	8	2.2
Positional + eQTL + Chromatin	39	45	6	2.3

Table 3: Properties of the gene networks obtained from different datasets (Section 4.2). Nodes are genes, which are linked when the corresponding gene model is significant.

Mapping	Genes	Edges	Components	Avg. degree
Standard	29	26	8	1.79
Positional	0	0	-	-
eQTL	11	7	5	1.27
Chromatin	10	5	5	1
eQTL + Chromatin	22	12	10	1.1
Positional + eQTL + Chromatin	23	13	10	1.1

and are expected to capture the variance explained by main effects. The observed effect of many significant SNP model notably decreased when we conditioned on singular SNPs in this way (Fig 2B), but not for all. The latter suggests a masking effect opposite to phantom epistasis. However, it is unclear how to adequately correct for multiple hypotheses testing after this adjustment in our setting, and in what follows we still use the unadjusted P-values, with the understanding that some of them may be inflated by weak correlations with main effects.

2.4 Gene epistasis: "functional" mappings boost discovery and interpretability

Findings of a GWAIS are often presented as a network, with nodes indicating SNPs and edges between nodes being present when the analysis protocol identifies the corresponding SNP pair as significantly interacting with the trait of interest. We converted SNP model networks into gene model epistasis networks (Fig 3), considering an edge between genes whenever gene model significance was obtained through the previously described ATPM approach (Section 4.2.4). The largest network was obtained under *Standard* mapping (26 edges). The *eQTL + Chromatin* combinations performed second best (12, 13 edges). Since no significant SNP pairs were detected under *Positional*, no significant gene pairs were produced either (Table 3).

For both *eQTL* and *Standard* most of the significant SNP models mapped to exclusively one gene model, removing possible sources of ambivalence (Fig 4A). That was less the case under the *Chromatin* analysis, where it was more common for the same SNP model to map to different gene models. We also investigated the relationship between significant gene models and the number of significant SNP models that mapped to them (Fig 4B). Most significant gene interactions were supported by relatively small numbers of SNPs: either few in number, or few with respect to the total number of SNP models for that significant gene model.

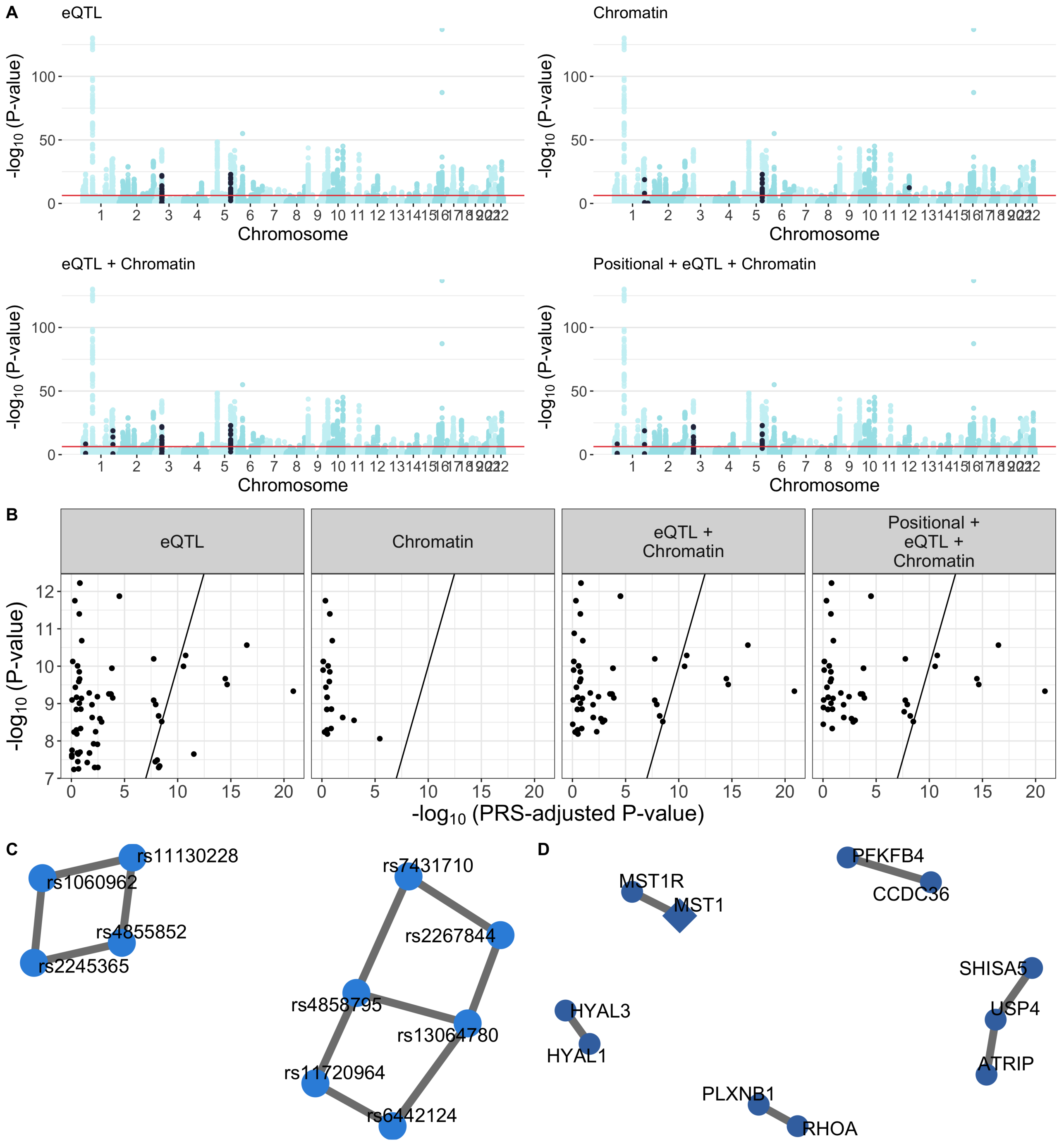

Fig 2: **(A)** Manhattan plot of the main effects, computed using logistic regression. In each subpanel, the SNPs selected via a significant SNP model, by each analysis, are colored in black. For reference, the Bonferroni threshold of main effects significance is displayed with a red horizontal line. **(B)** Comparison between the P-values of the significant SNP interactions, adjusted and unadjusted by main effects (x- and y-axis, respectively). P-values were not adjusted for multiple testing. To help interpretation, we added a $y = x$ line. **(C)** Network containing all the models significant in any of the analyses whose P-values after adjusting for PRS were lower than the original P-values. **(D)** Network containing all the gene models significant in any of the analyses that were mapped to one of the significant SNP models from panel C in its corresponding analysis.

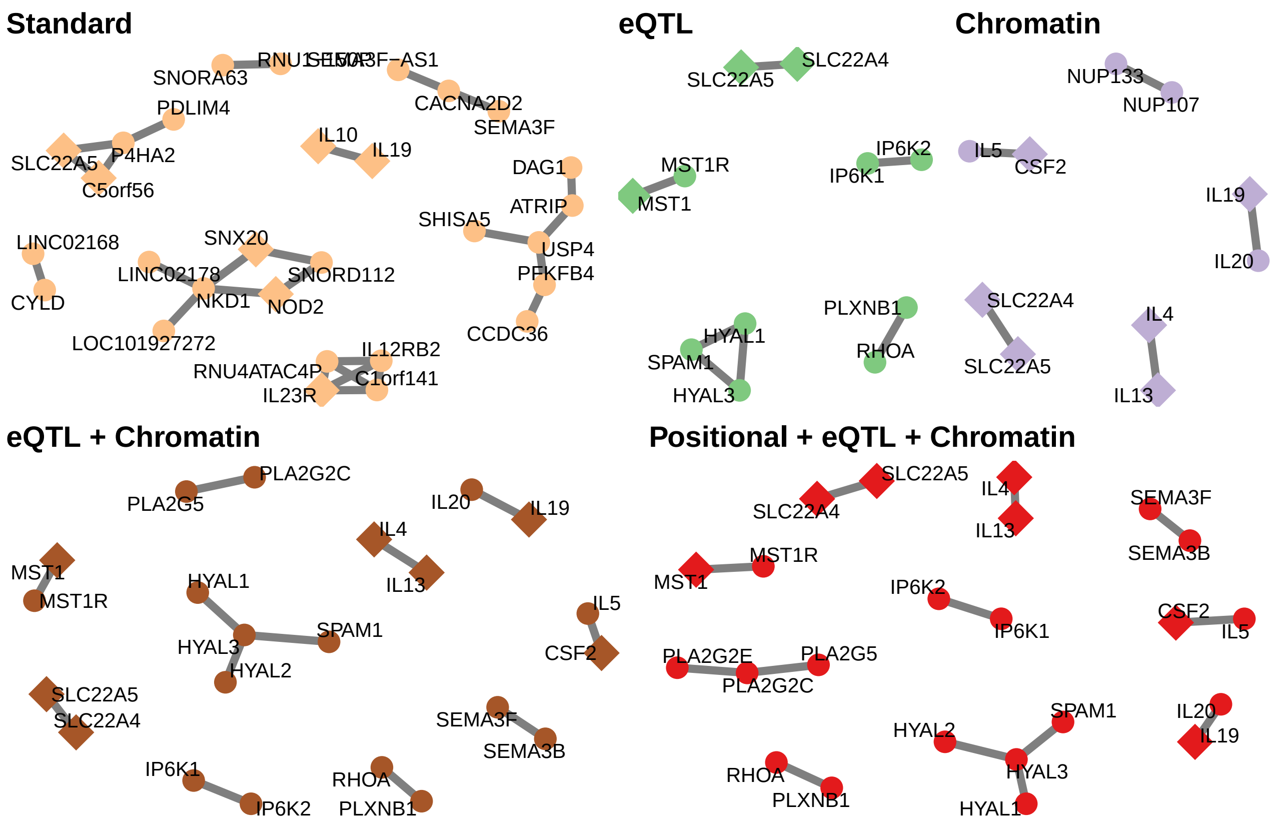

Fig 3: Epistasis networks built from derived significant gene models for the different analysis strategies (Section 4.2.1). The *Positional* analysis is absent, as no gene model was significant. Genes associated to IBD in DisGeNET [21] have a diamond shape.

2.5 Biofilter boosts discovery of interpretable hypotheses

Searching for epistatic interactions exclusively across edges of the Biofilter network greatly reduces the number of tests. Yet, this gain in statistical power might not lead to greater discoveries as it potentially disregards new interactions absent from databases. Hence, we tested whether exhaustively searching for epistasis on the datasets not reduced for Biofilter models but using each mapping, led to similar results. At the SNP level (Fig 5B, upper panel), only a small proportion of the significant interactions were still detected when the network was not used. Strikingly, that difference got smaller at the gene level (Fig 5B, lower panel). This suggests that the significant SNP models, even if fewer in number, are strong enough to lead to the detection of the gene models.

In a similar vein, we studied the number of interactions detected by considering the overlap between the significant models detected in the different analyses. Including more SNP-gene mappings in the analysis was mostly beneficial with respect to considering one mapping at a time, since both at the gene and at the SNP level, the significant interactions in *Positional + eQTL + Chromatin* highly overlapped with the other analyses (Fig 5A). Nonetheless, a few interactions were also missed in this joint analysis, in particular 20 significant SNP models detected in the *eQTL* analysis.

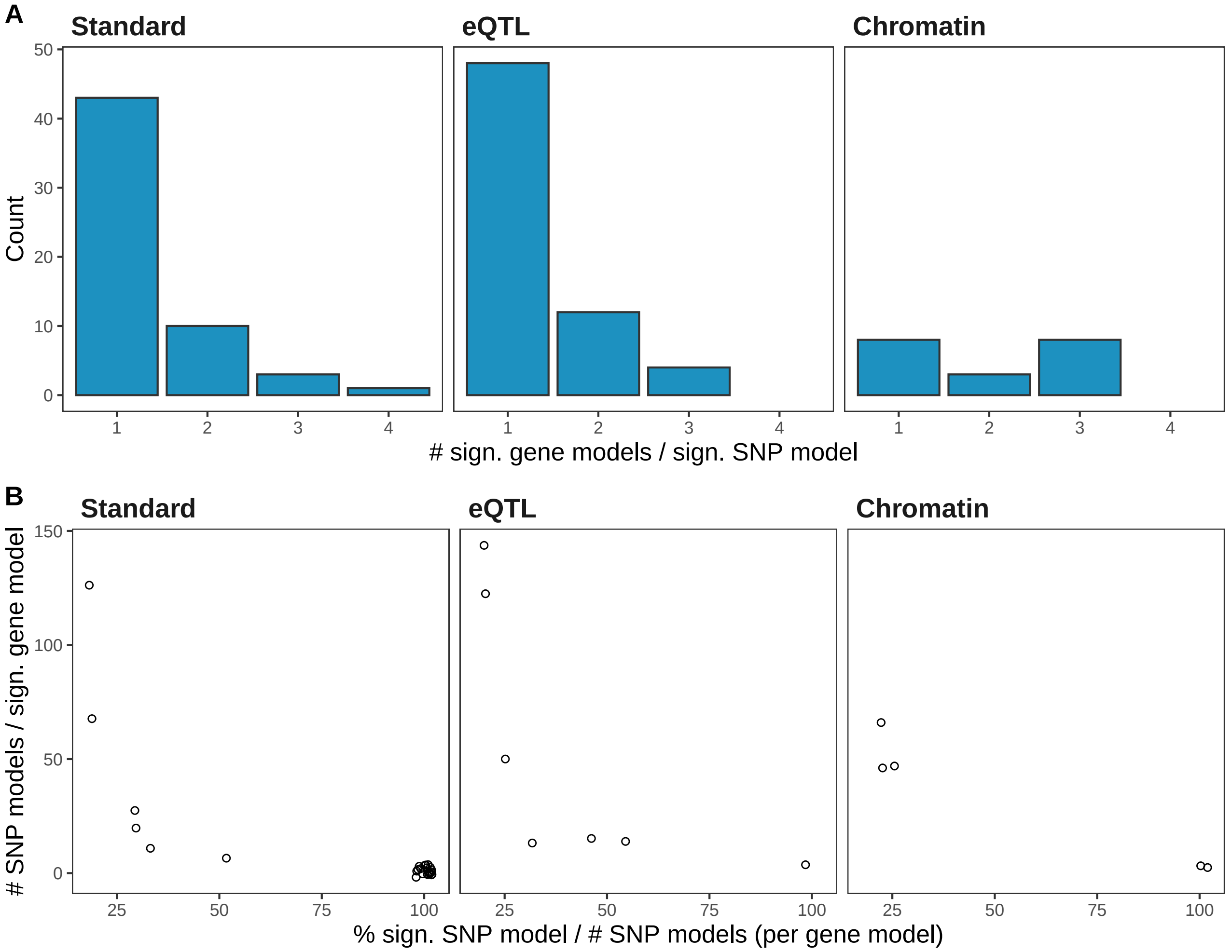

Fig 4: Relationship between the number of significant SNP models and of significant gene models. **(A)** Histogram of the number of significant gene models mapped to the same significant SNP model. **(B)** Relationship between the total number of SNP models mapped to the same significant gene model (y-axis), and the percentage of all the SNP models mapped to the same significant gene model that are significant themselves (x-axis). As multiple points can stack, we introduced a little Gaussian noise on each of them to improve visualization.

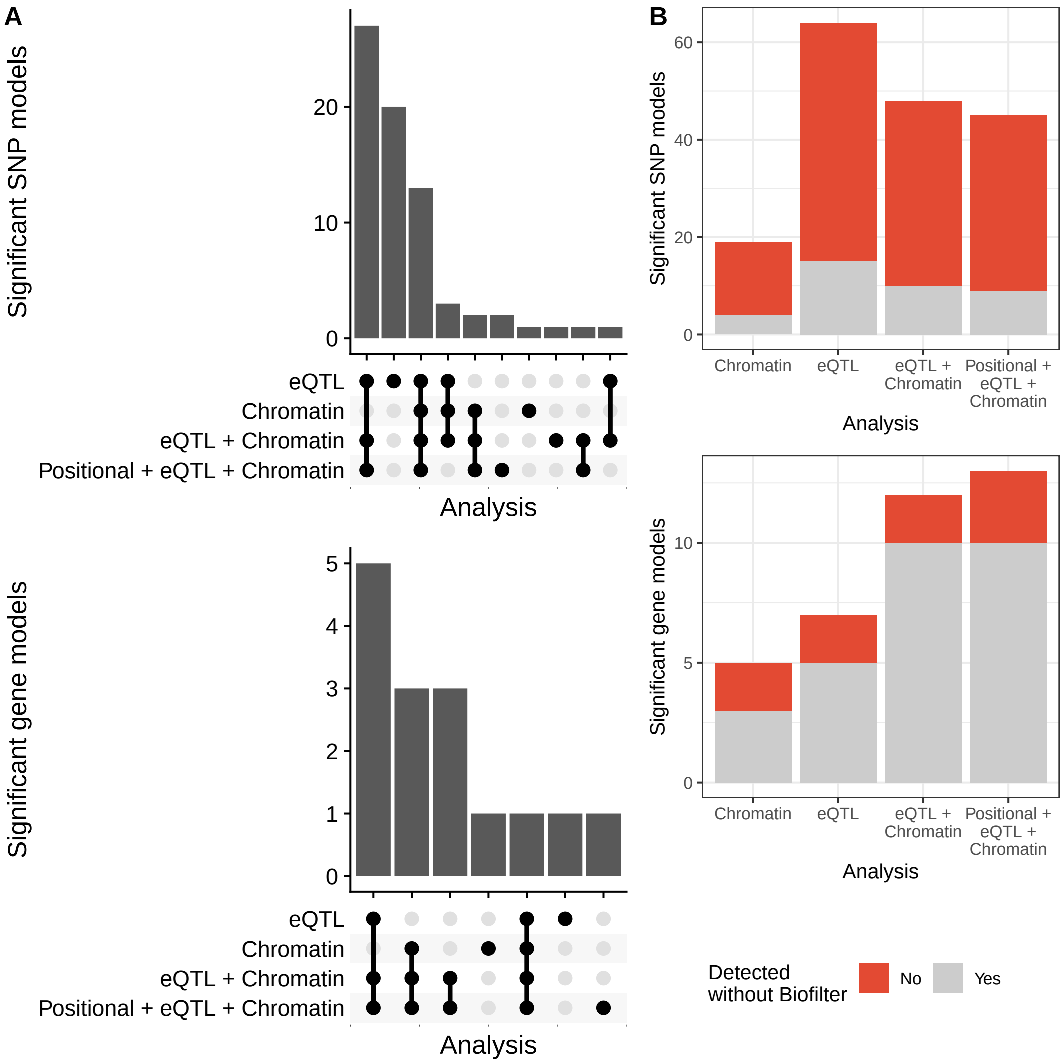

Fig 5: Comparison of the proposed analysis with relaxation of filters at different stages. **(A)** Impact of focusing on one SNP-gene mapping at a time, or at multiple at once. Overlap between the significant interactions detected in the different analyses. SNP interactions on top; gene interactions on the bottom. **(B)** Impact of focusing on interactions mappable to Biofilter interactions. Proportion of significant interactions that were detected using with and without filtering by SNPs mappable to Biofilter interactions. SNP interactions on top; gene interactions on bottom.

2.6 *Chromatin* and *Standard* analyses partially replicate previous studies on IBD

In the past, several genetic studies studying epistasis on IBD have been conducted [6, 9, 7, 10, 11, 22]. We compared them to our results at the gene level, the minimal functional unit at which we expect genetic studies to converge. Several epistatic alterations have been reported involving interleukins [6, 10, 11]. Also our *Standard* analyses resulted in interactions involving three interleukins (*IL-19*, *IL-10* and *IL-23*), although interacting with different genes than in the aforementioned studies. Functional analysis such as *Positional + eQTL + Chromatin* recovered five interleukins (*IL-4*, *IL-5*, *IL-13*, *IL-19*, *IL-20*). In addition, Lin et al. [22] detected interactions involving *NOD2*, with both *IL-23R* and other genes. Our *Standard* analysis highlighted two potentially new epistasic interactions involving *NOD2*.

Discoveries in the proposed protocol are guided by plausible biological interactions. Hence, every significant gene model can be traced back to a biological database, therefore producing biological hypotheses. For instance, the gene model *MST1*-*MST1R* is significant in multiple pipelines. Both genes have been linked to IBD, both by themselves [23, 24] and in interaction with other genes [25]. MST1R is a surface receptor of MST1, and, through physical interaction, they play a role in the regulation of inflammation.

2.7 Pathway analyses highlight the involvement of the extracellular matrix in IBD

Pathway enrichment analyses of each interaction's neighborhood (Section 4.3) allowed us to identify broader biological mechanisms that the significant interaction pairs might be involved in. The *eQTL* analysis thus produced multiple significant pathways (see Supplementary Table 1), involving the triangle of interactions formed by two genes located in 3p21.31 (*HYAL1*, *HYAL3*) and one in 7q31.32 (*SPAM1*) (Fig 3). The affected pathways were related to the extracellular matrix, and specifically to glycosaminoglycan degradation. Links between the turnover of the extracelular matric and IBD-related inflammation have been reported in the past [26]. More specifically, glycosaminoglycan [27] and hyaluronon [28] degradation products lead to inflammatory response. When restricting attention to pathways of minimum gene size 10 and maximum gene size 500 to avoid imbalances and non-normality, four pathways are removed: cellular response to UV B, hyaluronoglucosaminidase activity, hexosaminidase activity and CS/DS degradation. The *Chromatin* mapping and the *Standard* pipeline did not produce significant pathways.

2.8 The proposed pipeline increases reproducibility

GWAIS results are notoriously hard to reproduce. Hence, we studied whether our proposed pipeline led to more stable results. For that purpose, we ran the whole protocol again on a random subset of the data containing 80% of the samples. In each subset, 49% of the individuals were cases, respecting the initial proportion of cases and controls of the entire dataset. We repeated this experiment 10 times for each SNP-gene mapping. Conservatively, we used the same SNP and gene significance thresholds as for the corresponding entire dataset.

The *Standard* pipeline, which does not include Biofilter network-information, produced on average 11.4 significant gene models (standard error (SE) 1.1). With the *eQTL* (respectively *Chromatin*) analysis, we detected

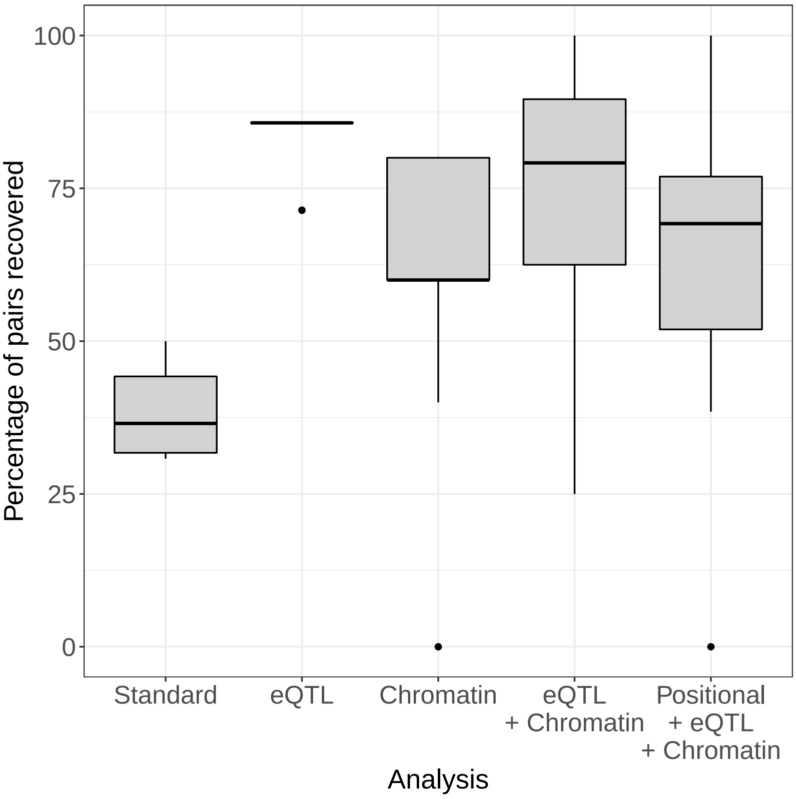

Fig 6: Robustness of the gene pairs produced within each mapping across ten repetitions using 80% of the data, i.e. percentage of gene pairs detected with the entire population that are recovered in the ten replicates with 80% of the individuals

on average 5.8 gene pairs (respectively 3.2) with SE 0.1 (respectively 0.4). Fig 6 shows that pipelines including biological knowledge recover more than 60% of the gene pairs detected with the entire cohort, on average, (83% for *eQTL* and 60% for *Chromatin* mapping), whereas without this knowledge (*Standard*), we recover less than 40% of the pairs. Hence, the *Standard* analysis appears to be the less robust in terms of conservation of gene pairs. This shows that filtering does increase robustness at the gene level. In addition, over the 10 repetitions, the *eQTL* analysis highlights significant pathways only once. The detected pathways are the same than those obtained with the entire population. No enriched pathways were found for the *Chromatin* or the *Standard* analysis.

3 Discussion

In this article we proposed a new protocol for epistasis detection, based on a variety of functional filtering strategies (Section 4.2), and studied its application to GWAS data for Inflammatory Bowel Disease (Section 4.1). The protocol included several components to control for type I error, hereby strengthening our belief in the discovered genetic interactions.

A common theme in the interpretation of epistasis results consists on linking the associated variants to an altered gene function. In this article, we considered 3 different such SNP-gene mappings. Notably, the number of SNP-gene correspondence provided by each mapping differed by orders of magnitude. Moreover,

the different mappings unevenly described genes; for instance, genes that had most SNPs mapped by using a chromatin contact map, had comparatively few eQTL SNPs. This motivated combining multiple mappings into an analysis (e.g. *eQTL + Chromatin*) in order to combine different perspectives of the epistasis process. For the most part, these complementary mappings improved the analyses, by recovering most of the interactions significant in the analyses that used one mapping at a time. Importantly, our results display the benefits of going beyond one single SNP-gene mapping (often, genomic position) to interpret epistasis results.

Restricting the tested interactions to functionally plausible pairs of genes and SNPs joins two faces of epistasis: searching for statistical epistasis, yet exclusively on plausible candidates for biological epistasis. This has several advantages. First, a more targeted input dataset reduces the number of tests and, in consequence, the multiple testing burden. In contrast, the high dimensionality of GWAIS data requires a much more stringent multiple testing correction and limits the detection of epistasis with low effect sizes. As we observed in Section 2.2, adopting one of the proposed analyses may reduce the number of SNP interactions to test by more than half. Yet, the *Standard* analysis, which does not use Biofilter, produces the most significant gene models. Second, the proposed protocol addresses the reproducibility issues widespread in GWAIS by producing results that are consistent at the gene and pathway levels (Section 2.5). Indeed, we observed an increased analytic robustness when using Biofilter gene models, in line with previous reports [29]. In particular, *eQTL* and *Chromatin* mappings, separate or in combination, increased said robustness. Third, restricting the search for epistasis to biologically plausible interactions yields results that are biologically interpretable and strikingly different from the ones obtained without using functional filtering (Section 2.4). Not surprisingly, different mappings also provided very different interaction signals and give resolution of information on different genes. In particular, in Section 2.6 we corroborated that the significant gene models from different functional filters were relevant to the biology of IBD. This was especially true for the *Chromatin* analysis (but also the *eQTL* analysis), giving rise to interactions with seemingly meaningful biological underpinnings, and stressing the relevance of regulatory variants in susceptibility to IBD. In contrast, the *Standard* analysis detected multiple interactions that were hard to interpret. For instance, several interactions involved RNA genes of unknown function (e.g. *LOC101927272* or *LINC02178*).

Remarkably, while the *Standard* analysis produced rich results, the *Positional* analysis did not lead to any significant SNP models. They both use genomic position to map SNPs to genes, but *Positional* is restricted to gene models in Biofilter. We note that the *Positional* analysis does not coincide with how Biofilter is typically used on GWAS data for epistasis detection. The latter involves pooling all SNPs that are mapped to genes which occur in Biofilter proposed gene interaction models, and subsequently exhaustive screening those SNPs for pairwise interactions. These pairs may also involve gene pairs that were not highlighted by Biofilter, in contrast to our *Positional* analysis. We evaluated the impact of biofilter on the final resuls. No significant SNP interaction were detected in *Positional* analysis. In the analysis without biofiltering (dataset reduced to mappable SNPs using genomic proximity, but not reduced to biofilter gene pairs), 62 pairs were significant. Also, on the 86 SNP interactions that passed the experimental threshold in the *Standard* analysis (dataset not reduced to mappable using genomic proximity, nor biofilter gene pairs), only 57 are mappable to gene

interactions using genomic proximity. Hence, 66% of significant SNP pairs are mappable via genomic proximity in the *Standard* analysis.

An important component of our protocol is the conversion of SNP-based epistasis to gene-based epistasis. The most popular approach consists in aggregating SNP-level P-values into gene-level statistics, which can be done in different ways (see [30] for some early examples, and [31] for recent developments). Here, we developed a generic approach that exploits a permutation strategy to define a P-value cutoff for SNP interactions, at a FWER of 5%, and then we followed the original implementation of the adaptive TPM (ATPM) to accommodate several truncation thresholds at once [32] while taking permutations instead of bootstrap as in Yu et al. [33]. The two algorithms are very similar, but we favored the TPM over the rank truncated product method of Yu et al. [33] that employs the product of the L most significant P-values, because the TPM only requires P-values smaller than a specified threshold, which is in line with the output of PLINK epistasis detection and saves storage space. Following both protocols and the recommendation of Becker and Knapp [34] we included measures derived from observed data in computing statistics under the null.

Remarkably, our proposed procedure keeps type I error under control, without additional corrections for multiple testing at the gene model. We hypothesize that this stems from two reasons. First, we apply a stringent correction for multiple testing at the SNP level. Second, when moving from SNP model significance to gene model significance, we restrict attention to significant SNP pairs in the ATPM. Hence, we do not consider any gene models that do not map to any such SNP model. However, alternative strategies could have been considered. For instance, not restricting ourselves to significant SNP models, hence conducting ATPM on all gene models. This could have led to increased discovery, in cases where the SNP models mapped to a gene tend to be low, albeit non-significant. However, it may also lead to an increased type I error. Accounting for that would require a multiple test correction at the gene level. In turn, such correction would be difficult since the dependency between the tests is unknown. Additionally, in common multiple test corrections this would require a much higher number of permutations, in order to obtain the necessary numerical precision.

How to best perform a pathway analysis of epistasis results is understudied. Often, all genes belonging to any significant gene pair are simply pooled together into a joint enrichment analysis. This approach discards the gene-gene interaction information that was, indeed, the object of analysis. Hence, in our procedure we adapted the *Network neighborhood search* protocol from Yip et al. [35], which considers the topology of the network using the shortest paths between the studied genes. It should be noted that we only used the topology to derive a neighborhood for each significant gene pair; then, we discarded the edge information. Yet, there are several directions for improvement. One is to exploit the topology of the epistasis network beyond the creation of a neighborhood. Another one is to take into account the gene size (or the number of SNPs per gene), for instance by performing a weighted version of the statistical test. Jia et al. [36] suggested a method for gene set enrichment analysis of GWAS data, adjusting the gene length bias or the number of SNP per gene. In our data, we did observe a link between the significance of the gene models and the number of SNPs mapped to the gene. For instance, in the *eQTL* analysis, the only one producing significant pathways, the median number of SNPs per genes is 385 among genes in significant pairs, versus 3 SNPs/gene genome-wide.

Several protocol changes may impact final results. As reported elsewhere [29], these changes or choices include the modelling framework (parametric, non-parametric, semi-parametric), encoding of the genetic markers, as well as LD handling. In this work, we used an additive encoding scheme (0, 1, 2 indicating the number of copies of the minor SNP allele), a popular choice in part because of its computational efficiency. However, this encoding schemes has been reported to tend to increase false positives (for instance [37]). This observation is based on type I error studies with data generated under the null hypothesis of no pairwise genetic interactions but in the presence of main effects (see for instance [38]). Here, we investigated the type I error control of our protocols under a general null hypothesis of no genetic associations with the trait (no interactions and no main effects) and established adequate control. As a consequence, this does not guarantee that our generated SNP interaction results were not overly-optimistic. To this end, we adjusted SNP-level epistasis P-values for main effects as comprised in a polygenic risk score. Not only does such a post-analysis adjustment via conditional regression reduce over-optimism due to inadequate control for lower-order effects, thus addressing phantom epistasis [19], but it may also occasionally highlight the masking of SNP interactions (as was shown in Fig 2B - $eQTL$). More work is needed to investigate the impact on gene-level interaction results, derived accordingly. For convenience, we used the regression framework to identify SNP interactions and relied on earlier recommendations regarding LD handling [39].

Our protocols are built on output from Biofilter, that can be presented as a co-functional gene network. One of the motivations was its proven ability to highlight meaningful interactions in a narrower alternative hypothesis space, at the expense of leaving parts of the interaction search space unexplored. The database that Biofilter built contained 37 266 interactions. This is notably smaller than other gene interaction databases, like HINT [40], 173 797 interactions), or STRING [41], 11 759 455 interactions). Testing gene interactions with other (combinations of) biological interaction networks was beyond the scope of this paper. Furthermore, Biofilter analysis or exhaustive screening may lead to non-overlapping results. An example within a regression context is given in [29].

4 Methods

4.1 Dataset and initial quality control

We investigated the IIBDGC dataset, produced by the International Inflammatory Bowel Disease Genetics Consortium (IIBDGC). The large sample size of this dataset helps overcoming the issue of reduced statistical power that is common in GWAIS. This dataset was genotyped on the Immunochip SNP array [42]. We performed quality control as in Ellinghaus et al. [43], hereby reducing the number of SNPs from 196 524 to 130 071. The final dataset contains 66 280 samples, out of which 32 622 are cases (individuals with IBD) and 33 658 are controls.

The IIBDGC dataset aggregates different cohorts, and contains potentially confounding population structure. As in Ellinghaus et al. [43], we used the first 7 principal components to model population stratification. Because

several epistasis detection methods, such as those implemented in PLINK [44], cannot include covariates in their logistic regression models, we instead adjusted the phenotypes by regressing out those principal components. In other words, we derived adjusted phenotypes from the logistic regression model by subtracting model-fitted values from observed phenotype values, i.e. response residuals (see Supplementary Fig 1).

4.2 Gene interaction detection procedure

As we describe in more detail below, we applied different functional filters to the available data. These filters use plausible interactions between genes, and three different ways of mapping SNPs to those genes, and hence, to these interactions. These three mappings exploit different degrees of biological knowledge to map SNPs to genes, referred to as *Positional*, *eQTL* and *Chromatin*. For each of the three SNP-to-gene mappings, we only analyzed the pairs of SNPs corresponding to a gene pair with prior evidence for interaction. In addition, we compared our findings in these scenarios to the *Standard* scenario, where all SNP pairs are analyzed without prior filtering. An overview of the entire pipeline is presented in Fig 7.

4.2.1 From gene models to SNP models

Although the unit of analysis in GWAIS is the SNP, biological interactions are often characterized at the gene level. Hence, we mapped all SNPs in the dataset to genes using FUMA [45], a post-GWAS annotation tool. We created an artificial input where every SNP is significant in order to perform such mapping on all the SNPs. We performed three SNP-gene mappings using FUMA's SNP2GENE: positional, eQTL and 3D chromatin interaction (Table 4). In the *Positional* mapping, we mapped a SNP to a gene when the genomic coordinates of former was within the boundaries of the latter $\pm$ 10 kb. The *eQTL* mapping uses eQTLs obtained from GTEx [46]. We mapped an eQTL SNP to its target gene when the association P-value was significant in any tissue (FDR < 0.05). Lastly, in the *Chromatin* mapping, we mapped a SNP to a gene when a contact had been observed between the former and the region around the latter's promoter in the 3D structure of the genome (250 bp upstream and 500 bp downstream from the transcription start site) in any of the Hi-C datasets included in FUMA (FDR $< 10^{-6}$). This mapping might contain new, undiscovered, regulatory variants which, as for SNPs obtained through eQTL mapping, regulate the expression of a gene.

4.2.2 Co-function gene and SNP networks

We used Biofilter 2.4 [18] to obtain candidate gene pairs to investigate for epistasis evidence. Biofilter generates pairs of genes susceptible to interact (*gene models*) with evidence of co-function across multiple publicly available biological databases. It includes genomic locations of SNPs and genes, as well as known relationships among genes and proteins such as interaction pairs, pathways and ontological categories, but does not use trait information. As per Biofilter's default, we used gene models supported by evidence in at least 2 databases. Additionally, we removed self-interactions, as detection of within-gene epistasis requires special considerations and is beyond the scope of this paper.

(A)

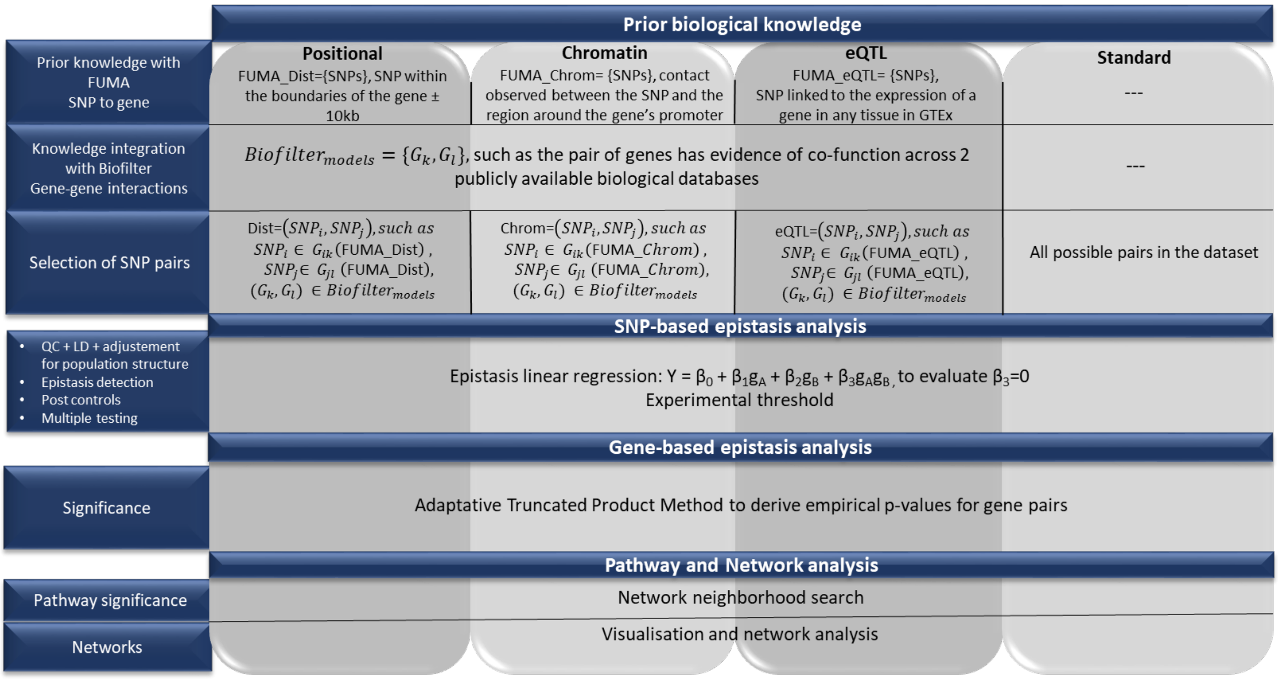

(B)

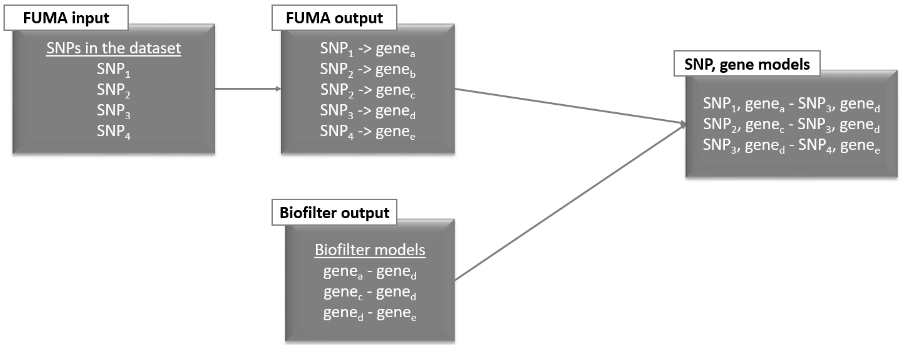

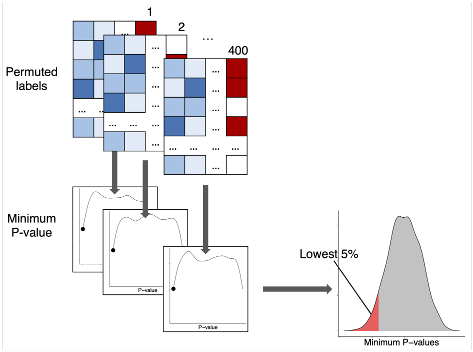

(C)

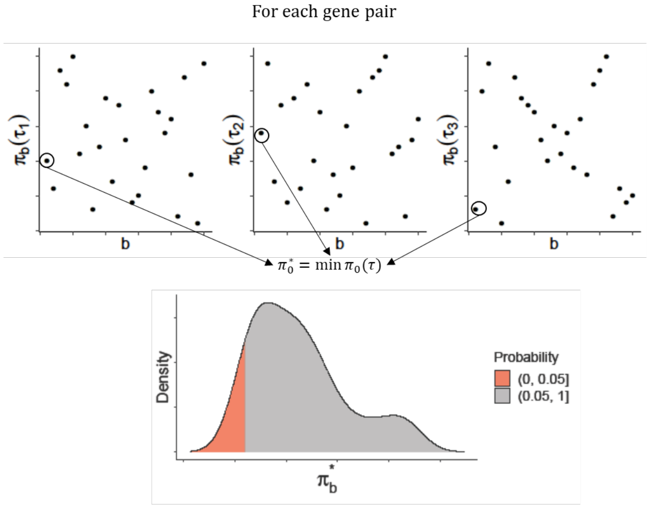

(D)

Fig 7: **(A)** Overview of the investigated gene-gene interaction detection protocols, described in Section 4.2. **(B)** Summary of the procedure to obtain SNP and gene models using FUMA and Biofilter, described in Section 4.2.2. **(C)** Permutation procedure to obtain the SNP model P-value threshold, described in Section 4.2.3. **(D)** Overview of the adaptive truncated product methodology, described in Section 4.2.4

Table 4: Properties of the different SNP-gene mappings and the filtered datasets. We show the empirical threshold of significance obtained through permutation, and the corresponding Bonferroni threshold for comparison.

Analysis	Mappings	SNP models (SNPs)	Empirical	Bonferroni[1]
Standard	-	7.3×10^8 (38 225)	1.1×10^{-10}	6.9×10^{-11}
Positional	1.7×10^5	3.0×10^5 (16 417)	1.6×10^{-7}	1.7×10^{-7}
Chromatin	2.4×10^6	6.0×10^6 (30 146)	1.0×10^{-8}	8.3×10^{-9}
eQTL	4.1×10^5	1.2×10^6 (16 652)	6.2×10^{-8}	4.0×10^{-8}
eQTL + Chromatin	2.7×10^6	9.0×10^6 (33 419)	6.5×10^{-9}	5.6×10^{-9}
Positional + eQTL + Chromatin	2.8×10^6	9.6×10^6 (34 548)	5.0×10^{-9}	5.2×10^{-9}

[1] Bonferroni threshold based on the number of possible SNP pairs in the analysis.

Given this set of gene models, and three different ways of obtaining *SNP models* from it, we removed all the SNPs that did not participate in any SNP model. Subsequently, we created six datasets. In one dataset no filter was applied (*Standard* analysis), i.e. no Biofiltering nor any SNP-to-gene mapping . Hence, the original SNP set was used. We also created one dataset exclusively for each SNP to gene mapping (*Positional*, *eQTL* and *Chromatin*). Lastly, we created two datasets using joint mappings: one with all the mappings (*Positional + eQTL + Chromatin*); and one with only the functional ones (*eQTL + Chromatin*). Since the main objective of this protocol is to increase the biological interpretability of epistasis findings, we have excluded other combinations that mix functional and non-functional information (*Positional + eQTL* and *Positional + Chromatin*).

We discarded SNP models involving rare variants (MAF $< 5\%$) or in Hardy–Weinberg equilibrium (P-value < 0.001). Regardless, all risk SNPs described in Liu et al. [47] were included, even when the aforementioned epistasis quality controls criteria did not hold up. Then, when the two SNPs of a *SNP model* were located in the HLA region, we discarded the pair, as it is difficult to differentiate between main and non-additive effects in this region [48]. Lastly, we discarded models where the SNPs were in linkage equilibrium ($r^2 > 0.75$), as motivated in Gusareva and Van Steen [39].

4.2.3 SNP-level epistasis detection and multiple testing correction

We used PLINK 1.9 to detect epistasis through a linear regression on the population structure adjusted phenotypes with the option `--epistasis`:

$$Y = \beta_0 + \beta_1 g_A + \beta_2 g_B + \beta_3 g_A g_B,$$

where g_A and g_B are the genotypes under additive encoding for SNPs A and B respectively, Y is the adjusted phenotype, and β_0, β_1, β_2, and β_3 are the regression coefficients. PLINK performs a statistical test to evaluate whether $\beta_3 \neq 0$. It only returns SNP pairs with a P-value lower than a specified threshold. We used the default 0.0001. Only SNP models were considered, apart from the *Standard* approach.

To correctly account for multiple testing, the P-value threshold of significance had to be dataset-dependent as the number of tested SNP pairs changed from dataset to dataset (Section 4.2.1). We obtained these thresholds

through permutations as in Hemani et al. [49] (Fig 7). More specifically, for each dataset, we permuted the phenotypes 400 times and fitted the aforementioned regression-based association model. This produced a null distribution of the extreme P-values for this number of tests given the LD structure in the data. For each dataset, we took the most extreme P-value from each of the 400 permutations and set the threshold for 5% family-wise error rate (FWER) to be the 5% percentile of these most extreme P-values. Posterior experiments showed that a higher number of permutations, 1 000, barely changed the empirical threshold (data not shown). Hence, 400 was a sufficient number of permutations to obtain an adequate threshold.

4.2.4 From SNP-level to gene-level epistasis

Our next step was to use significant SNP interactions to identify significant gene interactions, which requires combining the P-values of all SNP pairs mapped to the same gene pair. Suppose that SNP interaction tests have been conducted for N individual hypotheses $H_{0i}, i = 1, 2, \dots, N$, for example, N SNP models mapped to the same gene model. We tested the joint null hypothesis $H_0 = \bigcap_{i=1}^{n} H_{0i}$ at significance level α versus the combined alternative hypothesis H_1: at least one of H_{0i} is false. To do so, we considered all SNP pairs mapped to the same gene pair as a set of tests with the same global null hypothesis, and applied the Adaptive Truncated Product Method (ATPM) [32] (Fig 7).

ATPM is an adaptive variant of the Truncated Product Method (TPM) of Zaykin et al. [50], which uses as a statistic the product of the P-values smaller than some pre-specified threshold (here, significant SNP interactions) tests. More specifically, given a truncation point τ and a number N of significant SNP interactions, this test statistic is given as $W(\tau) = \prod_{i=1}^{N} p_i^{I(p_i \leq \tau)}$ where $I(\cdot)$ is the indicator function. TPM is interesting in our context because it does not require P-values for all SNP pairs but only for the most strongly associated ones.

The distribution of $W(\tau)$ under the null hypothesis is unknown when the individual tests are not independent, which is clearly the case here, but an empirical P-value $\hat{\pi}(\tau)$ can be estimated through permutations. Because the choice of τ is arbitrary, the adaptive version of TPM (ATPM) explores several values of τ and selects $\hat{\pi}^* = \min_\tau \hat{\pi}(\tau)$. The distribution of $\hat{\pi}^*$ under the null hypothesis can again be determined through permutations [51].

In our procedure, which is detailed below for a given gene pair, we used $B = 999$ permutations and $\tau \in \{0.001, 0.01, 0.05\}$. Remarkably, and following the suggestion of Becker and Knapp [34], the null distribution includes both the statistic from the observed dataset, and from the 999 permutations.

1. For each SNP model $i = 1, \dots, N$ mapped to the gene pair, compute its P-values $p_{i,b}$ in the original dataset ($b = 0$) and for each of the $B = 999$ permutations ($b = 1, \dots, B$).

2. For each value of τ and b, compute the test statistic $W(\tau)$.

3. For each value of τ and b, estimate the P-value : $\pi_b(\tau) = \frac{\sum_{l=0}^{B} I(W_b(\tau) \geq W_l(\tau))}{B+1}$.

4. For each value of b, compute $\pi_b^* = min_\tau \pi_b(\tau)$.

5. Estimate the P-value of the gene model as $P_0 = \frac{\sum_{l=0}^{B} I(\pi_0^* \geq \pi_l^*)}{B+1}$.

6. Reject the global null hypothesis if $P_0 \leq \alpha = 0.05$.

4.3 Pathway analysis

A pathway enrichment analysis on the neighborhood of a significant gene model can inform about the broader framework in which gene epistasis occurs. To define such neighborhoods, we adapted the network neighborhood search protocol from Yip et al. [35]. We computed the neighborhood of two genes as the list of all genes that (1) participate in any of the shortest paths between the two studied genes in the Biofilter network, once the direct link between them is removed; and (2) are also involved in a significant interaction with at least one other gene on these paths. We restricted our attention to neighborhoods containing at least 3 genes, including the 2 from the considered gene model. For each of these, we conducted a gene set enrichment analysis in relevant gene sets from the Molecular Signature Database (MSigDB version 7) [52, 53]. We performed the enrichment analysis using a hypergeometric test, which compares the obtained overlap between two sets to the expected overlap from taking equally-sized random sets from the universe of genes. We favored the hypergeometric test over the chi-square test used in Yip et al. [35] because the sample sizes of the neighborhoods were small and because chi-square is an approximation whereas the hypergeometric test is an exact test. The universe set was analysis dependant. It contained all genes in an annotated pathway and that can be mapped via genomic proximity to a SNP of the dataset for the *Standard* analysis, and genes present in Biofilter gene models, in an annotated pathway and that can be mapped via the appropriate SNP to gene mapping to a SNP of the dataset for the other analysis. Finally, pathways were said to be significant when the corresponding test P-value was lower than the Bonferroni threshold (0.05/(# pathways × # tested gene sets)), with *pathways* corresponding to pathways containing at least one gene of the neighborhood.

5 Code availability

The code necessary to reproduce this article's results and analyses is available on GitHub at `https://github.com/DianeDuroux/BiologicalEpistasis`. Additionally, we prepared `network_epistasis.nf`, a dataset agnostic Nextflow version of the proposed pipeline, available at `https://github.com/hclimente/gwas-tools`.

6 Conclusion

In this study we presented a protocol to enhance the interpretation of epistasis screening from GWAS. It includes gene-level epistasis discoveries with type I error under control, as well as a network-guided pathway analysis. Moreover, it improves the robustness of the results, making epistasis detection more reproducible. Aggregating SNP-level results into gene-level epistasis is challenging, but allows to include information from biological interaction databases. Based on that, we conducted multiple analyses that use different sources of prior biological knowledge about SNP-to-gene relationships and gene interaction models, as well as rigorous

statistical approaches to assess significance. Each of them offers a different, albeit complementary view of the disease, which leads to additional insights.

Their application to GWAS data for inflammatory bowel disease highlighted the potential of our strategy, including network-guided pathway analysis, as it recovered known aspects of IBD while capturing relevant and previously unreported features of its genetic architecture. These strategies will contribute to identify gene-level interactions from SNP data for complex diseases, and to enhance our belief in these findings.

Acknowledgements

We thank the International IBD Genetics Consortium for data collection and processing and for interesting discussions. This project has received funding from the European Union's Horizon 2020 research and innovation programme under the Marie Sklodowska-Curie grant agreement No 666003 and 813533. C-A.A. acknowledges funding from Agence Nationale de la Recherche (ANR-18-CE45-0021-01). Computational resources have been provided by the Consortium des Équipements de Calcul Intensif (CÉCI), funded by the Fonds de la Recherche Scientifique de Belgique (F.R.S.-FNRS) under Grant No. 2.5020.11 and by the Walloon Region. KVS acknowledges opportunities and funding provided by WELBIO (Walloon Excellence in Life sciences and BIOtechnology).

References

[1] Annalisa Buniello, Jacqueline A L MacArthur, Maria Cerezo, Laura W Harris, James Hayhurst, Cinzia Malangone, Aoife McMahon, et al. The NHGRI-EBI GWAS Catalog of published genome-wide association studies, targeted arrays and summary statistics 2019. *Nucleic Acids Research*, 47(D1):D1005–D1012, January 2019. ISSN 0305-1048, 1362-4962. doi: 10.1093/nar/gky1120. URL `https://academic.oup.com/nar/article/47/D1/D1005/5184712`. 00092.

[2] Hannah Gordon, Frederik Trier Moller, Vibeke Andersen, and Marcus Harbord. Heritability in inflammatory bowel disease: from the first twin study to genome-wide association studies. *Inflammatory bowel diseases*, 21(6):1428–1434, 2015.

[3] David Ellinghaus, Luke Jostins, Sarah L Spain, Adrian Cortes, Jörn Bethune, Buhm Han, Yu Rang Park, Soumya Raychaudhuri, Jennie G Pouget, Matthias Hübenthal, et al. Analysis of five chronic inflammatory diseases identifies 27 new associations and highlights disease-specific patterns at shared loci. *Nature genetics*, 48(5):510, 2016.

[4] Kelly A Shaw, David J Cutler, David Okou, Anne Dodd, Bruce J Aronow, Yael Haberman, Christine Stevens, Thomas D Walters, Anne Griffiths, Robert N Baldassano, et al. Genetic variants and pathways implicated in a pediatric inflammatory bowel disease cohort. *Genes & Immunity*, 20(2):131, 2019.

[5] Teri A. Manolio, Francis S. Collins, Nancy J. Cox, David B. Goldstein, Lucia A. Hindorff, David J. Hunter, et al. Finding the missing heritability of complex diseases. *Nature*, 461(7265):747–753, October 2009. ISSN

0028-0836, 1476-4687. doi: 10.1038/nature08494. URL http://www.nature.com/articles/nature08494. 06874.

[6] Zhenwu Lin, Zhong Wang, John P Hegarty, Tony R Lin, Yunhua Wang, Sue Deiling, Rongling Wu, Neal J Thomas, and Joanna Floros. Genetic association and epistatic interaction of the interleukin-10 signaling pathway in pediatric inflammatory bowel disease. *World journal of gastroenterology*, 23(27):4897, 2017.

[7] Christophe Pedros, Guillaume Gaud, Isabelle Bernard, Sahar Kassem, Marianne Chabod, Dominique Lagrange, Olivier Andréoletti, Anne S Dejean, Renaud Lesourne, Gilbert J Fournié, et al. An epistatic interaction between themis1 and vav1 modulates regulatory t cell function and inflammatory bowel disease development. *The Journal of Immunology*, 195(4):1608–1616, 2015.

[8] Jie Zhang, Zhi Wei, Christopher J Cardinale, Elena S Gusareva, Kristel Van Steen, Patrick Sleiman, and Hakon Hakonarson. Multiple epistasis interactions within mhc are associated with ulcerative colitis. *Frontiers in genetics*, 10:257, 2019.

[9] Severine Vermeire, Paul Rutgeerts, Kristel Van Steen, Sofie Joossens, G Claessens, Marie Pierik, Marc Peeters, and Robert Vlietinck. Genome wide scan in a flemish inflammatory bowel disease population: support for the ibd4 locus, population heterogeneity, and epistasis. *Gut*, 53(7):980–986, 2004.

[10] Dermot PB McGovern, Jerome I Rotter, Ling Mei, Talin Haritunians, Carol Landers, Carrie Derkowski, Deb Dutridge, Marla Dubinsky, Andy Ippoliti, Eric Vasiliauskas, et al. Genetic epistasis of il23/il17 pathway genes in crohn's disease dermot. *Inflammatory bowel diseases*, 15(6):883–889, 2009.

[11] Jürgen Glas, Johannes Stallhofer, Stephan Ripke, Martin Wetzke, Simone Pfennig, Wolfram Klein, Jörg T Epplen, Thomas Griga, Uwe Schiemann, Martin Lacher, et al. Novel genetic risk markers for ulcerative colitis in the il2/il21 region are in epistasis with il23r and suggest a common genetic background for ulcerative colitis and celiac disease. *The American journal of gastroenterology*, 104(7):1737, 2009.

[12] Jason H. Moore and Scott M. Williams. Traversing the conceptual divide between biological and statistical epistasis: systems biology and a more modern synthesis. *BioEssays*, 27(6):637–646, June 2005. ISSN 0265-9247, 1521-1878. doi: 10.1002/bies.20236. URL http://doi.wiley.com/10.1002/bies.20236. 00327.

[13] Xuesen Wu, Hua Dong, Li Luo, Yun Zhu, Gang Peng, John D Reveille, and Momiao Xiong. A novel statistic for genome-wide interaction analysis. *PLoS genetics*, 6(9):e1001131, 2010.

[14] Benjamin Lehne, Cathryn M Lewis, and Thomas Schlitt. From snps to genes: disease association at the gene level. *PloS one*, 6(6):e20133, 2011.

[15] Eric Jorgenson and John S Witte. A gene-centric approach to genome-wide association studies. *Nature reviews. Genetics*, 7:885–91, 12 2006. doi: 10.1038/nrg1962.

[16] Clément Niel, Christine Sinoquet, Christian Dina, and Ghislain Rocheleau. A survey about methods dedicated to epistasis detection. *Frontiers in Genetics*, 6, September 2015. ISSN 1664-8021. doi: 10.

3389/fgene.2015.00285. URL http://journal.frontiersin.org/Article/10.3389/fgene.2015.00285/abstract. 00000.

[17] Anja C Gumpinger, Bastian Rieck, Dominik G Grimm, International Headache Genetics Consortium, and Karsten Borgwardt. Network-guided search for genetic heterogeneity between gene pairs. *Bioinformatics*, page btaa581, June 2020. ISSN 1367-4803, 1460-2059. doi: 10.1093/bioinformatics/btaa581. URL https://academic.oup.com/bioinformatics/advance-article/doi/10.1093/bioinformatics/btaa581/5861532. 00000.

[18] Sarah A Pendergrass, Alex Frase, John Wallace, Daniel Wolfe, Neerja Katiyar, Carrie Moore, and Marylyn D Ritchie. Genomic analyses with biofilter 2.0: knowledge driven filtering, annotation, and model development. *BioData Mining*, 6(1), December 2013. ISSN 1756-0381. doi: 10.1186/1756-0381-6-25. URL http://biodatamining.biomedcentral.com/articles/10.1186/1756-0381-6-25. 00042.

[19] Gustavo de los Campos, Daniel Alberto Sorensen, and Miguel Angel Toro. Imperfect linkage disequilibrium generates phantom epistasis (& perils of big data). *G3: Genes, Genomes, Genetics*, 9(5):1429–1436, March 2019. doi: 10.1534/g3.119.400101. URL https://doi.org/10.1534/g3.119.400101.

[20] Shing Wan Choi and Paul F O'Reilly. Prsice-2: Polygenic risk score software for biobank-scale data. *Gigascience*, 8(7):giz082, 2019.

[21] Janet Piñero, Juan Manuel Ramírez-Anguita, Josep Saüch-Pitarch, Francesco Ronzano, Emilio Centeno, Ferran Sanz, and Laura I Furlong. The DisGeNET knowledge platform for disease genomics: 2019 update. *Nucleic Acids Research*, November 2019. doi: 10.1093/nar/gkz1021. URL https://doi.org/10.1093/nar/gkz1021.

[22] Zhenwu Lin, John P Hegarty, Gerrit John, Arthur Berg, Zhong Wang, Rishabh Sehgal, Danielle M Pastor, Yunhua Wang, Leonard R Harris, Lisa S Poritz, et al. Nod2 mutations affect muramyl dipeptide stimulation of human b lymphocytes and interact with other ibd-associated genes. *Digestive diseases and sciences*, 58 (9):2599–2607, 2013.

[23] John B. Beckly, Laura Hancock, Alessandra Geremia, Fraser J.R. Cummings, Andrew Morris, Rachel Cooney, Saad Pathan, Changcun Guo, and Derek P. Jewell. Two-stage candidate gene study of chromosome 3p demonstrates an association between nonsynonymous variants in the MST1r gene and crohn's disease. *Inflammatory Bowel Diseases*, 14(4):500–507, April 2008. doi: 10.1002/ibd.20365. URL https://doi.org/10.1002/ibd.20365.

[24] Paul R. Burton, David G. Clayton, Lon R. Cardon, Nick Craddock, Panos Deloukas, Audrey Duncanson, et al. Genome-wide association study of 14,000 cases of seven common diseases and 3,000 shared controls. *Nature*, 447(7145):661–678, June 2007. ISSN 1476-4687. doi: 10.1038/nature05911. URL https://doi.org/10.1038/nature05911. 00000.

[25] William K.K. Wu, Rui Sun, Tao Zuo, Yuanyuan Tian, Zhirong Zeng, Jeffery Ho, Justin C.Y. Wu, Francis K.L. Chan, Matthew T.V. Chan, Jun Yu, Joseph J.Y. Sung, Sunny H. Wong, Maggie H. Wang, and Siew C. Ng. A novel susceptibility locus in MST1 and gene-gene interaction network for crohn's disease in the chinese population. *Journal of Cellular and Molecular Medicine*, 22(4):2368–2377, February 2018. doi: 10.1111/jcmm.13530. URL `https://doi.org/10.1111/jcmm.13530`.

[26] Aaron C Petrey and A Carol. The extracellular matrix in ibd: a dynamic mediator of inflammation. *Current opinion in gastroenterology*, 33(4):234, 2017.

[27] Artin Soroosh, Sami Albeiroti, Gail A West, Belinda Willard, Claudio Fiocchi, and A Carol. Crohn's disease fibroblasts overproduce the novel protein kiaa1199 to create proinflammatory hyaluronan fragments. *Cellular and molecular gastroenterology and hepatology*, 2(3):358–368, 2016.

[28] Sami Albeiroti, Artin Soroosh, and Carol A de la Motte. Hyaluronan's role in fibrosis: a pathogenic factor or a passive player? *BioMed research international*, 2015, 2015.

[29] Kyrylo Bessonov, Elena S Gusareva, and Kristel Van Steen. A cautionary note on the impact of protocol changes for genome-wide association snp$\times$ snp interaction studies: an example on ankylosing spondylitis. *Human genetics*, 134(7):761–773, 2015.

[30] Li Ma, Andrew G Clark, and Alon Keinan. Gene-based testing of interactions in association studies of quantitative traits. *PLoS genetics*, 9(2), 2013.

[31] Olga A Vsevolozhskaya, Fengjiao Hu, and Dmitri V Zaykin. Detecting weak signals by combining small p-values in genetic association studies. *Frontiers in genetics*, 10:1051, 2019.

[32] Xuguang Sheng and Jingyun Yang. An adaptive truncated product method for combining dependent p-values. *Economics letters*, 119(2):180–182, 2013.

[33] Kai Yu, Qizhai Li, Andrew W Bergen, Ruth M Pfeiffer, Philip S Rosenberg, Neil Caporaso, Peter Kraft, and Nilanjan Chatterjee. Pathway analysis by adaptive combination of p-values. *Genetic Epidemiology: The Official Publication of the International Genetic Epidemiology Society*, 33(8):700–709, 2009.

[34] Tim Becker and Michael Knapp. A powerful strategy to account for multiple testing in the context of haplotype analysis. *The American Journal of Human Genetics*, 75(4):561–570, 2004.

[35] Danny Kit-Sang Yip, Landon L Chan, Iris K Pang, Wei Jiang, Nelson LS Tang, Weichuan Yu, and Kevin Y Yip. A network approach to exploring the functional basis of gene–gene epistatic interactions in disease susceptibility. *Bioinformatics*, 34(10):1741–1749, 2018.

[36] Peilin Jia, Lily Wang, Ayman H Fanous, Xiangning Chen, Kenneth S Kendler, Zhongming Zhao, International Schizophrenia Consortium, et al. A bias-reducing pathway enrichment analysis of genome-wide association data confirmed association of the mhc region with schizophrenia. *Journal of medical genetics*, 49(2):96–103, 2012.

[37] Kristel Van Steen and JH Moore. How to increase our belief in discovered statistical interactions via large-scale association studies? *Human genetics*, 138(4):293–305, 2019.

[38] Jestinah M Mahachie John, Tom Cattaert, François Van Lishout, Elena S Gusareva, and Kristel Van Steen. Lower-order effects adjustment in quantitative traits model-based multifactor dimensionality reduction. *PLoS One*, 7(1), 2012.

[39] Elena S. Gusareva and Kristel Van Steen. Practical aspects of genome-wide association interaction analysis. *Human Genetics*, 133(11):1343–1358, November 2014. ISSN 0340-6717, 1432-1203. doi: 10.1007/s00439-014-1480-y. URL `http://link.springer.com/10.1007/s00439-014-1480-y`. 00015.

[40] Jishnu Das and Haiyuan Yu. HINT: High-quality protein interactomes and their applications in understanding human disease. *BMC Systems Biology*, 6(1):92, 2012. ISSN 1752-0509. doi: 10.1186/1752-0509-6-92. URL `http://bmcsystbiol.biomedcentral.com/articles/10.1186/1752-0509-6-92`. 00204.

[41] Damian Szklarczyk, Annika L Gable, David Lyon, Alexander Junge, Stefan Wyder, Jaime Huerta-Cepas, et al. STRING v11: protein–protein association networks with increased coverage, supporting functional discovery in genome-wide experimental datasets. *Nucleic Acids Research*, 47(D1):D607–D613, January 2019. ISSN 0305-1048, 1362-4962. doi: 10.1093/nar/gky1131. URL `https://academic.oup.com/nar/article/47/D1/D607/5198476`. 00072.

[42] Adrian Cortes and Matthew A Brown. Promise and pitfalls of the Immunochip. *Arthritis Research & Therapy*, 13(1):101, 2010. ISSN 1478-6354. doi: 10.1186/ar3204. URL `http://arthritis-research.biomedcentral.com/articles/10.1186/ar3204`. 00451.

[43] David Ellinghaus, Sarah L Spain, Adrian Cortes, Jörn Bethune, Buhm Han, Yu Rang Park, et al. Analysis of five chronic inflammatory diseases identifies 27 new associations and highlights disease-specific patterns at shared loci. *Nature Genetics*, 48(5):510–518, May 2016. ISSN 1061-4036, 1546-1718. doi: 10.1038/ng.3528. URL `http://www.nature.com/articles/ng.3528`. 00214.

[44] Shaun Purcell, Benjamin Neale, Kathe Todd-Brown, Lori Thomas, Manuel AR Ferreira, David Bender, Julian Maller, Pamela Sklar, Paul IW De Bakker, Mark J Daly, et al. Plink: a tool set for whole-genome association and population-based linkage analyses. *The American journal of human genetics*, 81(3):559–575, 2007.

[45] Kyoko Watanabe, Erdogan Taskesen, Arjen van Bochoven, and Danielle Posthuma. Functional mapping and annotation of genetic associations with FUMA. *Nature Communications*, 8(1), December 2017. ISSN 2041-1723. doi: 10.1038/s41467-017-01261-5. URL `http://www.nature.com/articles/s41467-017-01261-5`. 00139.

[46] GTEx Consortium. Genetic effects on gene expression across human tissues. *Nature*, 550(7675):204–213, October 2017. ISSN 0028-0836, 1476-4687. doi: 10.1038/nature24277. URL `http://www.nature.com/articles/nature24277`. 00708.

[47] Jimmy Z Liu, Suzanne Van Sommeren, Hailiang Huang, Siew C Ng, Rudi Alberts, Atsushi Takahashi, Stephan Ripke, James C Lee, Luke Jostins, Tejas Shah, et al. Association analyses identify 38 susceptibility loci for inflammatory bowel disease and highlight shared genetic risk across populations. *Nature genetics*, 47(9):979, 2015.

[48] JA Traherne. Human mhc architecture and evolution: implications for disease association studies. *International journal of immunogenetics*, 35(3):179–192, 2008.

[49] Gibran Hemani, Konstantin Shakhbazov, Harm-Jan Westra, Tonu Esko, Anjali K. Henders, Allan F. McRae, et al. Detection and replication of epistasis influencing transcription in humans. *Nature*, 508 (7495):249–253, April 2014. ISSN 0028-0836, 1476-4687. doi: 10.1038/nature13005. URL `http://www.nature.com/articles/nature13005`. 00162.

[50] Dmitri V Zaykin, Lev A Zhivotovsky, Peter H Westfall, and Bruce S Weir. Truncated product method for combining p-values. *Genetic Epidemiology: The Official Publication of the International Genetic Epidemiology Society*, 22(2):170–185, 2002.

[51] Youngchao Ge, Sandrine Dudoit, and Terence P Speed. Resampling-based multiple testing for microarray data analysis. *Test*, 12(1):1–77, 2003.

[52] A. Subramanian, P. Tamayo, V. K. Mootha, S. Mukherjee, B. L. Ebert, M. A. Gillette, A. Paulovich, S. L. Pomeroy, T. R. Golub, E. S. Lander, and J. P. Mesirov. Gene set enrichment analysis: A knowledge-based approach for interpreting genome-wide expression profiles. *Proceedings of the National Academy of Sciences*, 102(43):15545–15550, September 2005. doi: 10.1073/pnas.0506580102. URL `https://doi.org/10.1073/pnas.0506580102`.

[53] Arthur Liberzon, Chet Birger, Helga Thorvaldsdóttir, Mahmoud Ghandi, Jill P. Mesirov, and Pablo Tamayo. The molecular signatures database hallmark gene set collection. *Cell Systems*, 1(6):417–425, December 2015. doi: 10.1016/j.cels.2015.12.004. URL `https://doi.org/10.1016/j.cels.2015.12.004`.

Supporting information

Supporting information

Interpretable network-guided epistasis detection

Supplementary Table 1: eQTL enriched Pathways

GO hyaluronoglycosaminidase activity
GO hexosaminidase activity
KEGG glycosaminoglycan degradation
GO hydrolase activity hydrolyzing o glycosyl compounds
GO hydrolase activity acting on glycosyl bonds
NABA ecm regulators
GO response to UV B
GO hyaloronan catabolic process
REACTOME hyaluronan metabolism
GO hyaluronan metabolic process
REACTOME chondoitrin sulfate dermatan sulfate
GO aminoglycan catabolic process
NABA matrisome associated
GO cellular response to UV B
REACTOME CS DS degradation
REACTOME hyaluronan uptake and degradation

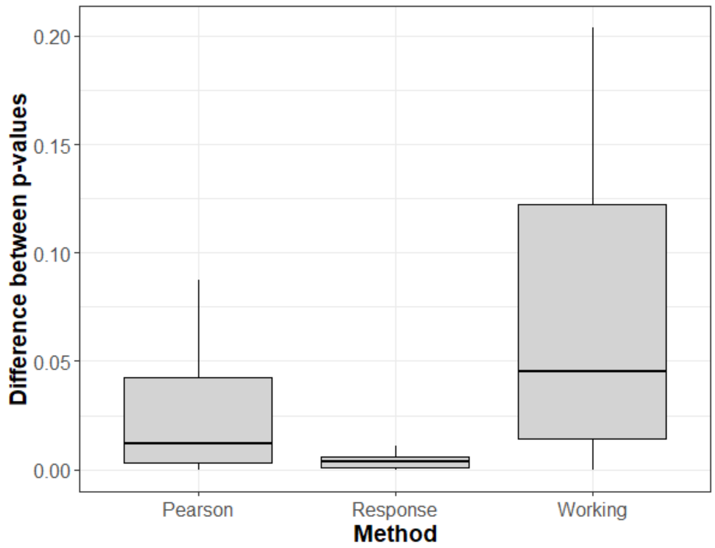

Supplementary Fig 1: To choose the best way of computing residuals in order to obtain the phenotype adjusted for population structure, we randomly extracted five SNPs in the dataset (rs12488468, rs1005678, rs11714286, rs2267844, rs11720964) and compared the associated outputs of epistasis detection. First, we computed the different residuals: we ran a logistic regression model with binary phenotypes as response variable and 7 PCs as independent variables. We derived three vectors of adjusted phenotypes from response, working and Pearson residuals. Then, we looked for statistical epistasis: we computed three linear models using the different residuals as response variable and SNP interactions as dependent variables. Finally, we performed logistic regressions with the binary phenotype as dependent variable, two SNPs and their interaction as explanatory variables, in addition to 7 PCs as covariates. We aimed at identifying the residuals leading to P-values as close as possible to the P-values from the logistic regression. P-values obtained with response residuals as phenotypes are the closest to the ones obtained with the logistic regression and are therefore selected as adjusted phenotypes in our analysis.

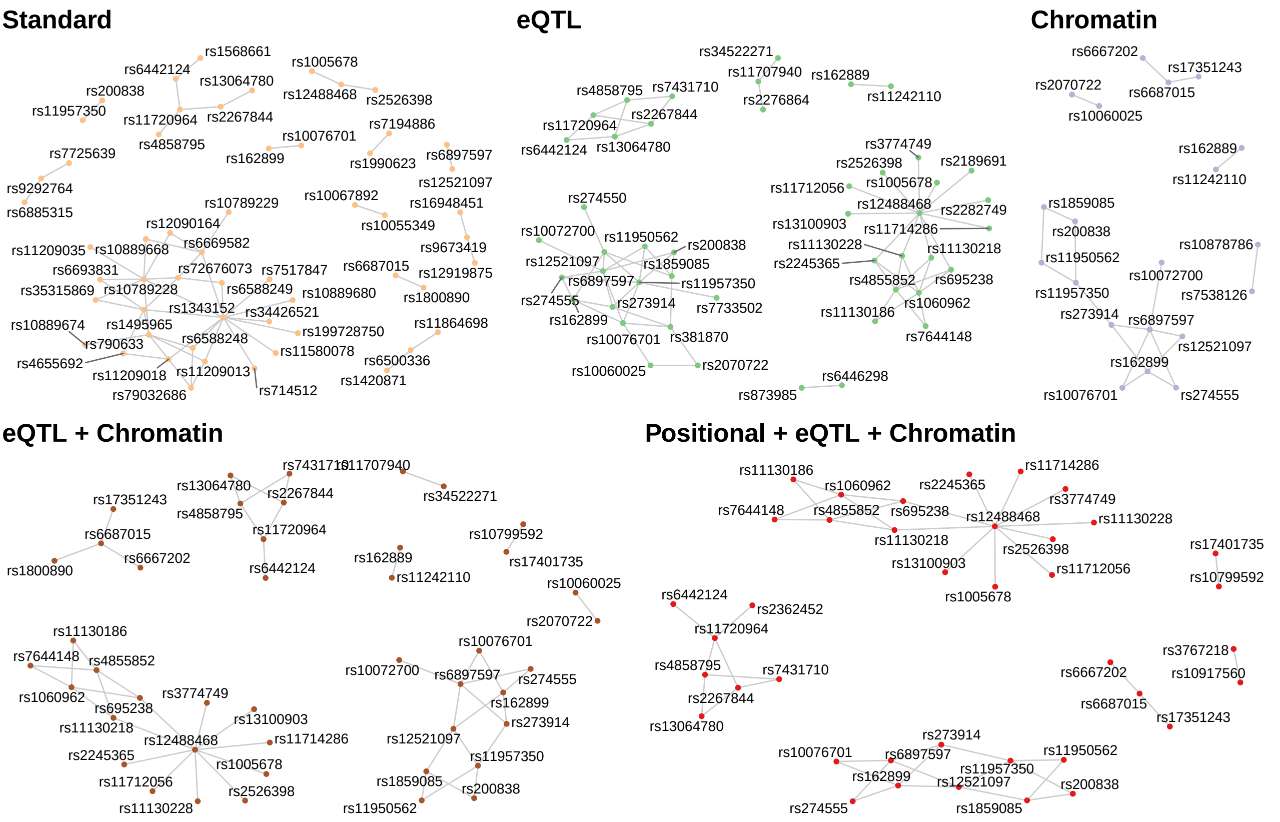

Supplementary Fig 2: Epistasis networks built from the significant SNP models of the different analysis. The *Positional* analysis is absent, as no SNP model was significant.
