# Supplementary material for: Interpretable network-guided epistasis detection
Source: Gigascience. 2022 Feb 4;11:giab093. doi: 10.1093/gigascience/giab093 (PMC8848319; doi:10.1093/gigascience/giab093)
Supplement: giab093_GIGA-D-21-00039_Revision_1 [file giab093_giga-d-21-00039_revision_1.pdf]

GigaScience

Interpretable network-guided epistasis detection

--Manuscript Draft--

**Manuscript Number:**	GIGA-D-21-00039R1
**Full Title:**	Interpretable network-guided epistasis detection
**Article Type:**	Research
**Funding Information:**	RIKEN — Dr Héctor Climente-González<br>H2020 Marie Skłodowska-Curie Actions () (666003) — Dr Chloé-Agathe Azencott<br>H2020 Marie Skłodowska-Curie Actions (813533) — Dr Kristel Van Steen<br>Agence Nationale de la Recherche (FR) (ANR-18-CE45-0021-01) — Dr Chloé-Agathe Azencott<br>WELBIO — Dr Kristel Van Steen
**Abstract:**	Detecting epistatic interactions at the gene level is essential to understanding the biological mechanisms of complex diseases. Unfortunately, genome-wide interaction association studies (GWAIS) involve many statistical challenges that make such detection hard. We propose a multi-step protocol for epistasis detection along the edges of a gene-gene co-function network. Such an approach reduces the number of tests performed and provides interpretable interactions, while keeping type I error controlled. Yet, mapping gene-interactions into testable SNP-interaction hypotheses, as well as computing gene pair association scores from SNP pair ones, is not trivial. Here we compare three SNP-gene mappings (positional overlap, eQTL and proximity in 3D structure) and used the adaptive truncated product method to compute gene pair scores. This method is non-parametric, does not require a known null distribution, and is fast to compute. We apply multiple variants of this protocol to a GWAS inflammatory bowel disease (IBD) dataset. Different configurations produced different results, highlighting that various mechanisms are implicated in IBD, while at the same time, results overlapped with known disease biology. Importantly, the proposed pipeline also differs from a conventional approach were no network is used, showing the potential for additional discoveries when prior biological knowledge is incorporated into epistasis detection.
**Corresponding Author:**	Héctor Climente-González<br>RIKEN<br>Tokyo, JAPAN
**Corresponding Author Secondary Information:**	
**Corresponding Author's Institution:**	RIKEN
**Corresponding Author's Secondary Institution:**	
**First Author:**	Diane Duroux
**First Author Secondary Information:**	
**Order of Authors:**	Diane Duroux<br>Héctor Climente-González<br>Chloé-Agathe Azencott<br>Kristel Van Steen
**Order of Authors Secondary Information:**	
**Response to Reviewers:**	Dear Dr. Edmunds, dear reviewers,<br><br>We thank you for your consideration, attention to detail, and positive feedback on our manuscript. We have read and discussed the comments you raised with great interest

	and tried to address them in detail below (responses in blue). We believe this peer-review step has substantially improved the quality and comprehensiveness of the manuscript. We attach to this letter a final version of the article, and a marked version with all the changes.<br><br>We summarize here the main changes to the manuscript:<br><br>- We have adapted our manuscript to GigaScience's guidelines, including the appropriate sections when necessary. Notably, since GigaScience's articles do not number sections and subsections, we removed some of the cross-references to Sections and replaced others with named references or the most relevant Figure/Table.<br>- Moving the Methods after the Analysis section required adding context to some of the parts of the Analysis.<br>- We have included two additional families of analyses:<br>- Tissue-specific eQTL and chromatin interactions<br>- Positional + eQTL and Positional + chromatin analyses<br>At the same time, we have removed the results about eQTL + Chromatin from the last sections of the article.<br>- We replaced all the networks layout for a circular one, which facilitates comparing them.<br>- We have replaced our claims about the reproducibility of the pipeline with the more accurate (as per our experiments) robustness.<br>- We have fixed typos and added clarifications.<br><br>Additionally, we attach a letter in which we address all the comments from the reviewers.
**Additional Information:**	
**Question**	**Response**
Are you submitting this manuscript to a special series or article collection?	No
**Experimental design and statistics**<br><br>Full details of the experimental design and statistical methods used should be given in the Methods section, as detailed in our Minimum Standards Reporting Checklist. Information essential to interpreting the data presented should be made available in the figure legends.<br><br>Have you included all the information requested in your manuscript?	Yes
**Resources**<br><br>A description of all resources used, including antibodies, cell lines, animals and software tools, with enough information to allow them to be uniquely identified, should be included in the Methods section. Authors are strongly	Yes

Powered by Editorial Manager® and ProduXion Manager® from Aries Systems Corporation

encouraged to cite Research Resource Identifiers (RRIDs) for antibodies, model organisms and tools, where possible.<br><br>Have you included the information requested as detailed in our Minimum Standards Reporting Checklist?	
**Availability of data and materials**<br><br>All datasets and code on which the conclusions of the paper rely must be either included in your submission or deposited in publicly available repositories (where available and ethically appropriate), referencing such data using a unique identifier in the references and in the "Availability of Data and Materials" section of your manuscript.<br><br>Have you have met the above requirement as detailed in our Minimum Standards Reporting Checklist?	Yes

Powered by Editorial Manager® and ProduXion Manager® from Aries Systems Corporation

Manuscript
Click here to access/download;Manuscript;SNP2GENE_main.pdf

OXFORD (GIGA)^n SCIENCE

GigaScience, 2017, 1–15

doi: xx.xxxx/xxxx
Manuscript in Preparation
Paper

PAPER

Interpretable network-guided epistasis detection

Diane Duroux[1,†], Héctor Climente-González[2,3,4,5,†], Chloé-Agathe Azencott[4,2,3] and Kristel Van Steen[1,6,*]

[1]BIO3 - GIGA-R Medical Genomics, University of Liege and [2]Institut Curie, PSL Research University and [3]INSERM, U900 and [4]MINES ParisTech, PSL Research University and [5]RIKEN Center for Advanced Intelligence Project (AIP) and [6]BIO3 - Department of Human Genetics, KU Leuven

[*]kristel.vansteen@uliege.be
[†]Contributed equally.

Abstract

Detecting epistatic interactions at the gene level is essential to understanding the biological mechanisms of complex diseases. Unfortunately, genome-wide interaction association studies (GWAIS) involve many statistical challenges that make such detection hard. We propose a multi-step protocol for epistasis detection along the edges of a gene-gene co-function network. Such an approach reduces the number of tests performed and provides interpretable interactions, while keeping type I error controlled. Yet, mapping gene-interactions into testable SNP-interaction hypotheses, as well as computing gene pair association scores from SNP pair ones, is not trivial. Here we compare three SNP-gene mappings (positional overlap, eQTL and proximity in 3D structure) and use the adaptive truncated product method to compute gene pair scores. This method is non-parametric, does not require a known null distribution, and is fast to compute. We apply multiple variants of this protocol to a GWAS inflammatory bowel disease (IBD) dataset. Different configurations produced different results, highlighting that various mechanisms are implicated in IBD, while at the same time, results overlapped with known disease biology. Importantly, the proposed pipeline also differs from a conventional approach were no network is used, showing the potential for additional discoveries when prior biological knowledge is incorporated into epistasis detection.

Key words: Gene-gene interaction; Inflammatory Bowel Disease; systems biology; biology-informed analysis; epistasis network

Background

Genome-wide association studies (GWAS) have identified over 70 000 genetic variants associated with complex traits [1]. Often these variants altogether do not explain the whole variance of a trait. A representative example is inflammatory bowel disease (IBD), like Crohn's disease and ulcerative colitis. Pooled twin studies estimate their heritabilities at 0.75 and 0.67 respectively [2]. Yet, despite large GWAS that identified over 200 IBD-associated loci [3], a low proportion of their variance has been explained [4]. Possible explanations include a large number of common variants with small effects, rare variants with large effects not covered in GWAS, unaccounted gene-environment interactions, and genetic interactions [5]. In this article we explore the latter, called epistasis, which has been linked to IBD in the past [6, 7, 8, 9, 10, 11]. Often, two types of epistasis are described: biological and statistical epistasis [12]. Broadly described, biological epistasis refers to a physical interaction between two biomolecules that has an impact on the phenotype. Statistical epistasis refers to departures from population-level linear models describing relationships between predictive factors such as alleles at different genetic loci.

Genome-wide association interaction studies (GWAIS) focus on the detection of statistical epistasis. To date, these studies have produced few replicable, functional conclusions, and specific gene-gene interactions have rarely been identified. This may be due to the small effects sizes of the interac-

Compiled on: October 12, 2021.
Draft manuscript prepared by the author.

Key Points

- We propose an epistasis detection protocol that exploits prior knowledge on how genes relate to each other (using a gene-gene network), and how SNPs relate to genes (positional overlap, expression regulation, or chromatin interactions).
- The proposed protocol reduces number of tested interactions, and provides more interpretable results.
- Applied to an inflammatory bowel disease GWAS dataset, our protocol recovers both known genes and interactions previously reported, as well as potentially novel mechanisms.

tions, the low statistical power, or the absence of a widely accepted GWAIS protocol. Even in the absence of statistical challenges, GWAIS are usually conducted on single nucleotide polymorphisms (SNPs), and SNP-interactions often lack a straightforward functional interpretation. Moving from SNP- to gene-level tests, which jointly consider all the SNPs mapped to the same gene, might address both shortcomings. First, aggregating SNP pair statistics into gene pair statistics is likely to increase the statistical power when dealing with complex diseases [13]. Second, converting statistical findings into biological hypotheses [14] may facilitate their functional interpretability [15].

To both reduce the number of tests and improve the interpretablity of significant SNP interactions, some authors propose examining only pairs of SNPs likely to be functionally related [16]. Such approaches use prior biological knowledge, for instance, of SNPs involved in genes that establish a protein-protein interaction [17]. Yet, limiting studies to one particular kind of gene-gene interaction might be reductive. To tackle that issue, Pendergrass et al. [18] developed Biofilter, a gene-gene co-function network, which aggregates multiple databases. Additionally, such approaches often require as well a proper mapping of SNP to genes.

In this article, we propose guiding the search for statistical epistasis using plausible biological epistasis. Taking exclusively interactions reported from at least 2 different sources in Biofilter, we compile a subset of gene-gene interactions that are biologically plausible. Then, we exclusively search for those interactions in a GWAIS dataset, reducing the multiple test burden and improving the interpretability. We investigate different ways of mapping SNPs to genes and use the adaptive truncated product method [19] to estimate the association of gene pairs. Network and pathway analyses are used to further assist in the interpretation of epistasis findings. The proposed pipeline is applied to GWAS data from the International IBD Genetic Consortium [3].

Data description

We investigated the IIBDGC dataset, produced by the International Inflammatory Bowel Disease Genetics Consortium (IIBDGC). This dataset was genotyped on the Immunochip SNP array [20]. We performed quality control as in Ellinghaus et al. [21], hereby reducing the number of SNPs from 196 524 to 130 071. The final dataset contains 66 280 samples, out of which 32 622 are cases (individuals with IBD) and 33 658 are controls. The large sample size of this dataset helps overcoming the issue of reduced statistical power that is common in GWAIS.

The IIBDGC dataset aggregates different cohorts, and contains potentially confounding population structure. As in Ellinghaus et al. [21], we used the first 7 principal components to model population stratification. Because several epistasis detection methods, such as those implemented in PLINK [22], cannot include covariates in their logistic regression models, we instead adjusted the phenotypes by regressing out those principal components. In other words, we derived adjusted phenotypes from the logistic regression model by subtracting model-fitted values from observed phenotype values, i.e. response residuals (see Supplementary Fig 1).

Analysis

SNP to gene mapping: Chromatin contacts map more SNPs per gene than other mappings

In this article, we present a pipeline to detect gene epistasis across the edges of a network. We extract interacting pairs of genes from the gene-gene Biofilter network to obtain candidate gene epistatic pairs (*gene models*). We considered three ways to match genes to SNPs and obtain *SNP models* from them: *Positional*, *eQTL* and *Chromatin* (detailed in section *From gene models to SNP models*). *Chromatin* produced the largest number of unique SNP-gene mappings (2 394 590), an order of magnitude more than *eQTL* (411 120) and *Positional* (174 879) (Table 4). The *Chromatin* mapping had on average the largest number of SNPs mapped on to a gene, followed by *eQTL* and *Positional* (Fig 1A). Nonetheless, the number of SNPs mapped to a gene varied considerably across genes (Fig 1B). In addition, the number of SNPs mapped to a same gene varied considerably across analyses (Fig 1C, D and E): in general, the genes with most SNPs mapped using the *eQTL* mapping had relatively few SNPs mapped in the *Chromatin* mapping, and vice versa.

The *Positional* analysis does not recover any SNP interaction

The aforementioned SNP-gene mappings, and combinations of them (cross-mappings), yielded seven sets of SNP models. Running our pipeline on them resulted in seven epistatic SNP-SNP networks described in Table 1 (for visualization, see Supplementary Fig 2). We also conducted what we called a *Standard* analysis, which reflects a conventional epistasis detection procedure. In this one, we exhaustively searched for epistatic interactions between all the SNPs that passed quality control. Then, we used positional mapping to assign gene interactions to the significant SNP interactions. Strikingly, while the *Standard* analysis generated the largest SNP-interaction network (55 nodes/SNPs and 57 edges/interactions), the *Positional + eQTL* one was the largest by number of interactions (76). The *Positional* analysis produced no significant interactions at all.

Gene epistasis: “functional” mappings boost discovery and interpretability

Findings of a GWAIS are often presented as a network, with nodes indicating SNPs and edges between nodes being present when the analysis protocol identifies the corresponding SNP

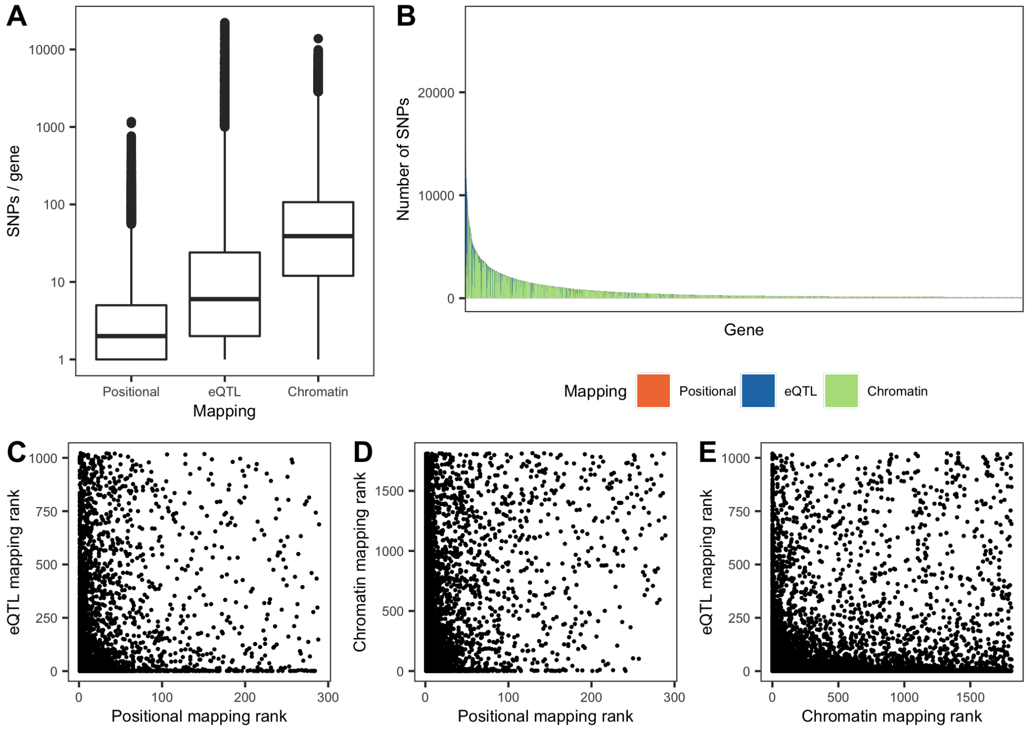

Figure 1. **(A)** Number of SNPs per gene for each of the three mappings described in *From gene models to SNP models*. **(B)** Ranking of genes with most SNPs mapped using any of the mappings, colored by mapping. Only genes with more than 100 SNPs mapped to it are displayed. **(C,D,E)** Comparison between the rank of each gene according to the number of SNPs mapped to it using each mapping.

Table 1. Properties of the SNP networks obtained from different datasets. Nodes are SNPs, which are linked when the SNP model is significant.

Analysis	SNPs	Edges	Components	Avg. degree
Standard	55	57	12	2.07
Positional	0	0	-	-
eQTL	46	64	6	2.78
Chromatin	20	19	5	1.9
eQTL + Chromatin	44	48	8	2.2
Positional + eQTL	43	76	6	2.7
Positional + Chromatin	21	39	5	1.9
Positional + eQTL + Chromatin	39	45	6	2.3

pair as significantly interacting with the trait of interest. We converted SNP model networks into gene model epistasis networks (Fig 2), adding an edge between two genes whenever the corresponding gene model significance was ascertained through an ATPM approach. The largest network was obtained under the *Standard* analysis (26 edges, Table 2). The *Positional + eQTL + Chromatin* combinations performed second best (13 edges). Since no significant SNP pairs were detected under *Positional*, no significant gene pairs were produced either. Similarly, the two analyses that included *Positional* information on top of another source barely added any new information in comparison to *Positional + eQTL + Chromatin*: *Positional + eQTL* included a new gene pair (*SPAM1-HYAL1*, already detected in the *eQTL* analysis), while *Positional + Chromatin* did not produce any additional pair. On the same line, *eQTL + Chromatin* included only one gene model absent from *Positional + eQTL + Chromatin*: *PLA2G2E-PLA2G2C*. Hence, we removed those three from further analyses, since *Positional + eQTL + Chromatin* captured more biological signal than those separately.

For both *eQTL* and *Standard* most of the significant SNP models mapped to exclusively one gene model, removing possible sources of ambivalence (Fig 3A). That was less the case under the *Chromatin* analysis, where it was more common for the same SNP model to map to different gene models. We also investigated the relationship between significant gene models and the number of significant SNP models that mapped to them (Fig 3B). Most significant gene interactions were supported by relatively small numbers of SNPs: either few in number, or few with respect to the total number of SNP models for that significant gene model.

Significant SNP pairs are near each other and near loci with main effects

Notably, the SNPs in significant SNP interactions are located near each other in the genome (the median distance between the pair of SNPs in *Chromatin*, *eQTL* and *Positional + eQTL + Chromatin* was 161 kbp). Moreover, they tend to overlap with GWAS main effects loci (Fig 4A). To investigate whether main effects could be driving some of the signals, even when in imperfect LD with epistatic SNP pairs (a phenomenon sometimes referred to as "phantom epistasis" [24]), we conducted a linear regression-based test, including a vector of polygenic risk scores as covariate. The observed effect of many significant SNP model notably decreased when we conditioned on single SNPs in this way (Fig 4B), but not for all. The latter suggests

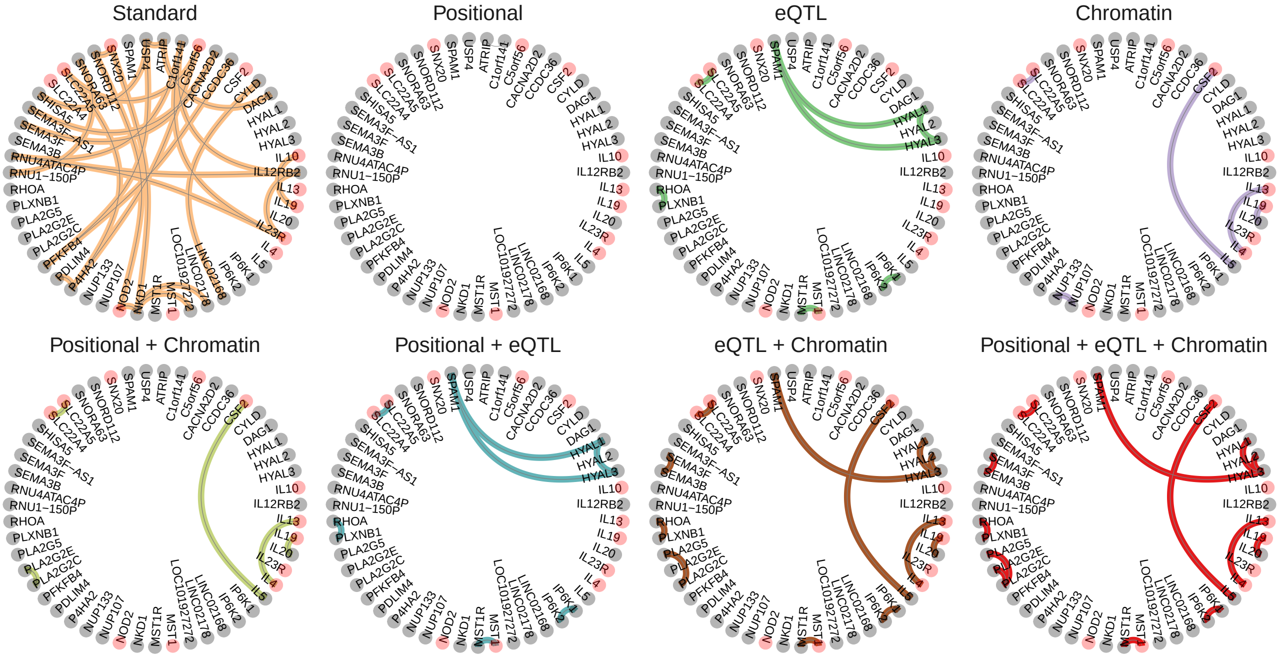

Figure 2. Epistasis networks built from derived significant gene models for the different analysis strategies. Genes associated to IBD in DisGeNET [23] are colored in pink. An alternative layout of the networks is available in Supplementary Fig 3.

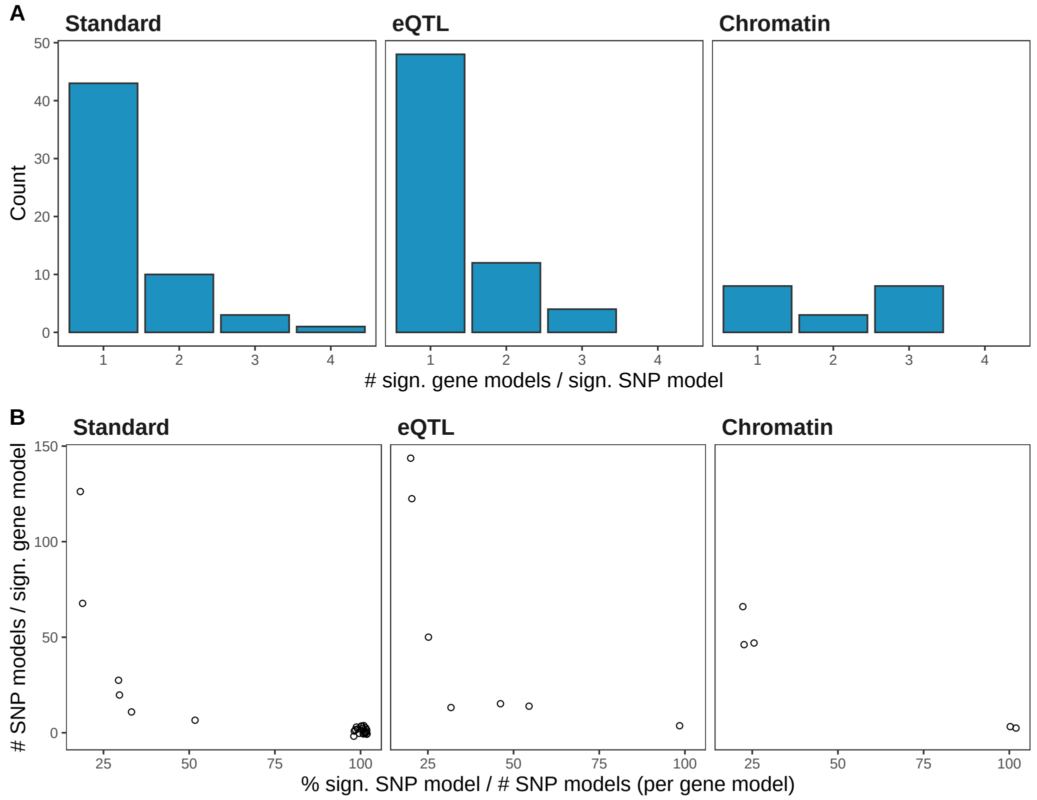

Figure 3. Relationship between the number of significant SNP models and of significant gene models. **(A)** Histogram of the number of significant gene models mapped to the same significant SNP model. **(B)** Relationship between the total number of SNP models mapped to the same significant gene model (y-axis), and the percentage of all the SNP models mapped to the same significant gene model that are significant themselves (x-axis). As multiple points can stack, we introduced a little Gaussian noise on each of them to improve visualization.

Table 2. Properties of the gene networks obtained from different datasets. Nodes are genes, which are linked when the corresponding gene model is significant.

Mapping	Genes	Edges	Components	Avg. degree
Standard	29	26	8	1.79
Positional	0	0	-	-
eQTL	11	7	5	1.27
Chromatin	10	5	5	1
eQTL + Chromatin	22	12	10	1.1
Positional + eQTL	11	7	5	1.3
Positional + Chromatin	10	5	5	1
Positional + eQTL + Chromatin	23	13	10	1.1

a masking effect opposite to phantom epistasis. However, it is unclear how to adequately correct for multiple hypotheses testing after this adjustment in our setting, and in what follows we still use the unadjusted P-values, with the understanding that some of them may be inflated by weak correlations with main effects.

The type I error is controlled

To evaluate the statistical relevance of the detected gene interactions, we studied if the proposed protocol controlled the type I error. For that purpose, we performed a permutation analysis based on 1 000 permutations for each of the datasets, permuting the phenotypes and running the entire protocol to detect significant gene interactions. This permutation procedure is independent of the one used in the proposed protocol to compute significance thresholds. When at least one significant gene interactions was observed in a permutation, that permutation was considered a false positive (FP). This allowed us to compute the type I error rate as $\frac{\# \text{FP}}{1000}$. Type I error was under control in all tested experimental settings, with estimates $\leq$ 6.6% (Table 3).

Biofilter boosts discovery of interpretable hypotheses

Searching for epistatic interactions exclusively across edges of the Biofilter network greatly reduces the number of tests. Yet, this gain in statistical power might not lead to greater discoveries as it potentially disregards new interactions absent from databases. Hence, we tested whether exhaustively searching for epistasis on the datasets not reduced for Biofilter models but using each mapping, led to similar results. At the SNP level (Fig 5A, upper panel), only a small proportion of the significant interactions were still detected when the network was not used. Strikingly, that difference got smaller at the gene level (Fig 5A, lower panel). This suggests that the significant SNP models, even if fewer in number, are strong enough to lead to the detection of the gene models.

In a similar vein, we studied the overlap between the significant models detected in the different analyses. Including more SNP-gene mappings in the analysis was mostly beneficial with respect to considering one mapping at a time, since both at the gene and at the SNP level, the significant interactions in *Positional + eQTL + Chromatin* highly overlapped with the other analyses (Fig 5B). Nonetheless, a few interactions were also missed in this joint analysis, in particular 20 significant SNP models detected in the *eQTL* analysis.

Chromatin and *Standard* analyses partially replicate previous studies on IBD

In the past, several genetic studies have investigated epistasis on IBD [6, 7, 9, 10, 11, 25]. We compared them to our results at the gene level, the minimal functional unit at which we expect genetic studies to converge. Several epistatic alterations have been reported involving interleukins [6, 10, 11]. Also our *Standard* analyses resulted in interactions involving three interleukins (*IL-19*, *IL-10* and *IL-23*), although interacting with different genes than in the aforementioned studies. *Positional + eQTL + Chromatin* recovered five interleukins (*IL-4*, *IL-5*, *IL-13*, *IL-19*, *IL-20*). In addition, Lin et al. [25] detected interactions involving *NOD2*, with both *IL-23R* and other genes. Our *Standard* analysis highlighted two potentially new epistasic interactions involving *NOD2*.

Discoveries in the proposed protocol are guided by plausible biological interactions. Hence, every significant gene model can be traced back to a biological database, therefore producing biological hypotheses. For instance, the gene model *MST1-MST1R* is significant in multiple pipelines. Both genes have been linked to IBD, both by themselves [26, 27] and in interaction with other genes [28]. MST1R is a surface receptor of MST1, and, through physical interaction, they play a role in the regulation of inflammation.

Pathway analyses highlight the involvement of the extracellular matrix in IBD

Pathway enrichment analyses of each interaction's neighborhood allowed us to identify broader biological mechanisms that the significant interaction pairs might be involved in. The *eQTL* analysis produced multiple significant pathways (see Supplementary Table 1), involving the triangle of interactions formed by two genes located in 3p21.31 (*HYAL1*, *HYAL3*) and one in 7q31.32 (*SPAM1*) (Fig 2). The affected pathways were related to the extracellular matrix, and specifically to glycosaminoglycan degradation. Links between the turnover of the extracelular matric and IBD-related inflammation have been reported [29]. More specifically, glycosaminoglycan [30] and hyaluronon [31]

Table 3. Type I error of the protocol presented in *Gene interaction detection procedure*, estimated over 1 000 random permutations, as explained in section *The type I error is controlled*.

Analysis	Average num. of significant interactions under H_0	Type 1 error (%)
Standard	0.05	3.6
Positional	0.04	3.7
eQTL	0.09	4.2
Chromatin	0.07	6.6
Positional + eQTL + Chromatin	0.04	3.6

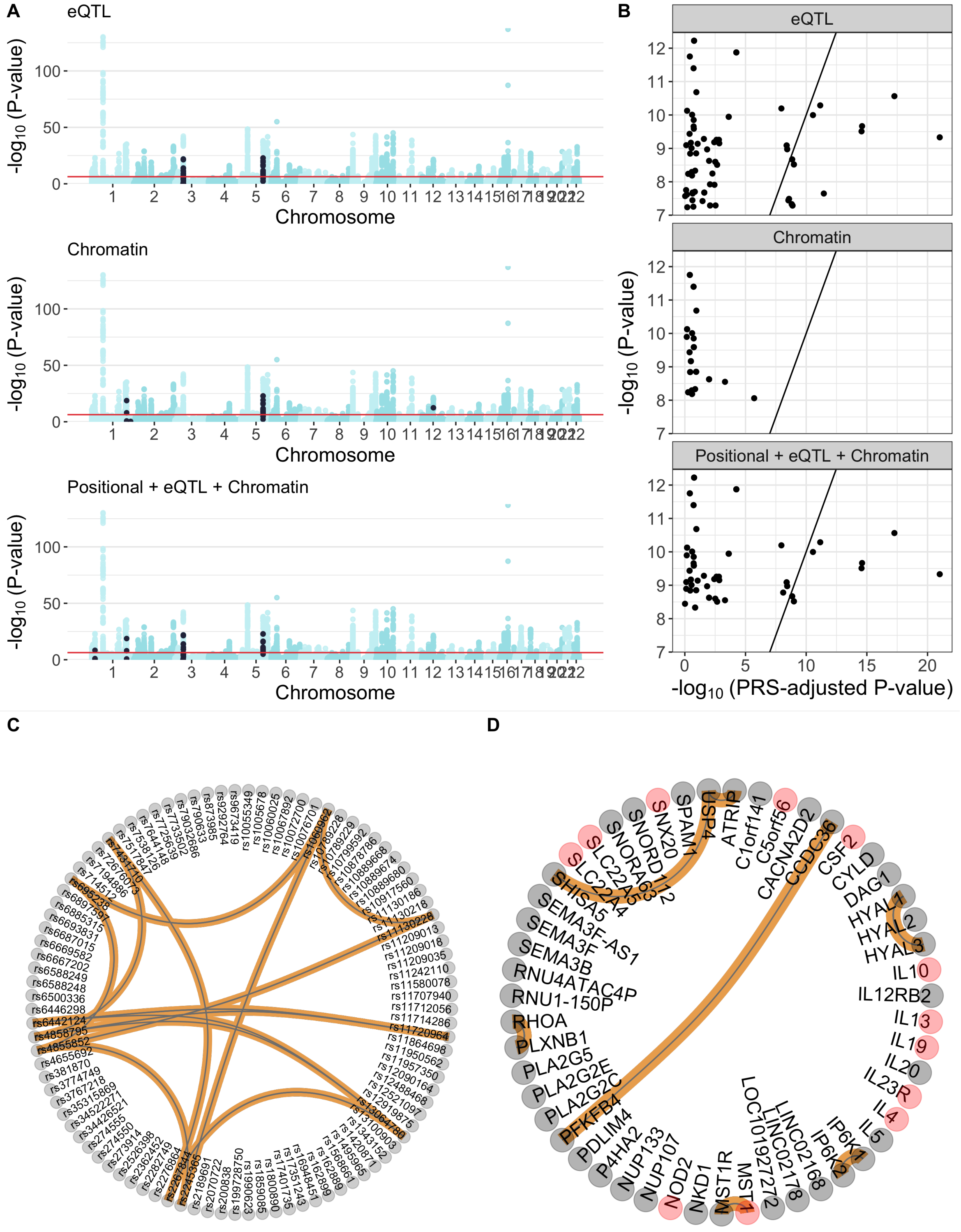

Figure 4. **(A)** Manhattan plot of the main effects, computed using logistic regression. In each subpanel, the SNPs selected via a significant SNP model, by each analysis, are colored in black. For reference, the Bonferroni threshold of main effects significance is displayed with a red horizontal line. **(B)** Comparison between the P-values of the significant SNP interactions, adjusted and unadjusted by main effects (x- and y-axis, respectively). P-values were not adjusted for multiple testing. To help interpretation, we added a $y = x$ line. **(C)** Network containing all the SNP models significant in any of the analyses whose P-values after adjusting for PRS were lower than the original P-values. **(D)** Network containing all the gene models significant in any of the analyses that were mapped to one of the significant SNP models from panel C in its corresponding analysis. Genes associated to IBD in DisGeNET [23] are colored in pink.

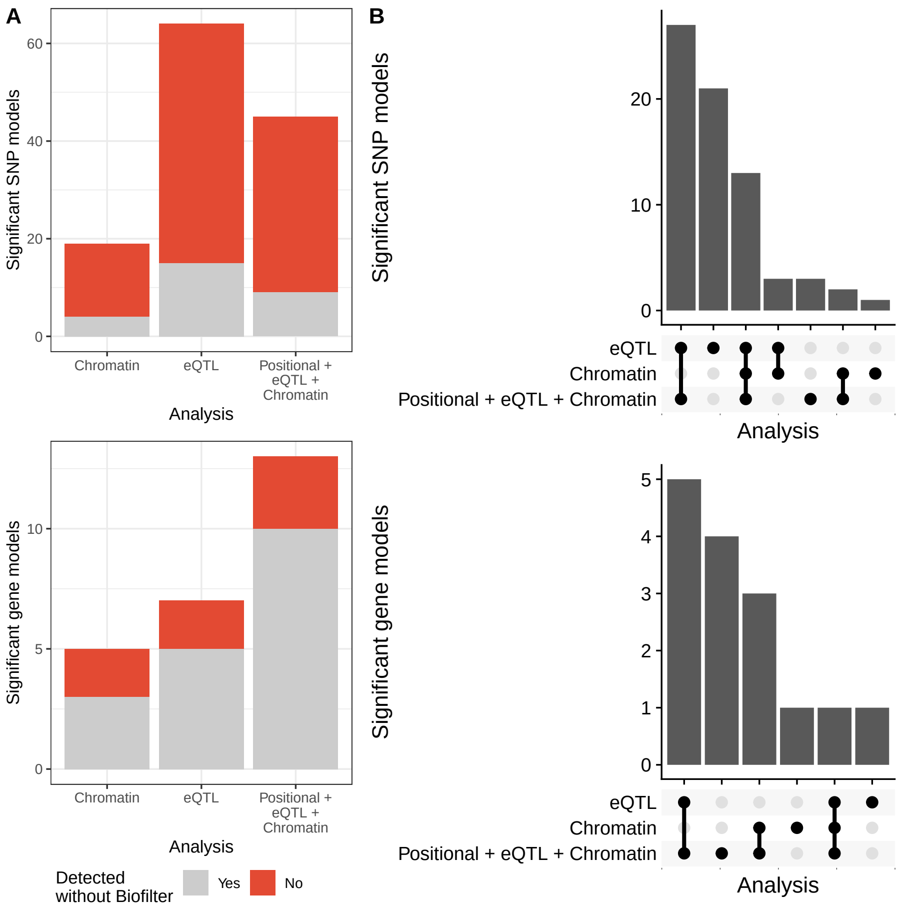

Figure 5. Comparison of the proposed analysis with relaxation of filters at different stages. **(A)** Impact of focusing on one SNP-gene mapping at a time, or at multiple at once. Overlap between the significant interactions detected in the different analyses. SNP interactions on top; gene interactions on the bottom. **(B)** Impact of focusing on interactions mappable to Biofilter interactions. Proportion of significant interactions that were detected using with and without filtering by SNPs mappable to Biofilter interactions. SNP interactions on top; gene interactions on bottom.

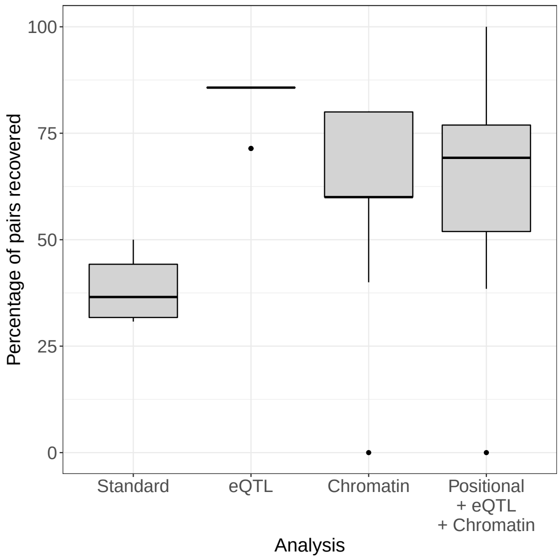

Figure 6. Gene pairs produced within each mapping across ten repetitions using 80% of the data, i.e. percentage of gene pairs detected with the entire dataset that are recovered in the ten subsets with 80% of the individuals.

degradation products lead to inflammatory response. When restricting attention to pathways of minimum gene size 10 and maximum gene size 500 to avoid imbalances and non-normality, four pathways are removed: cellular response to UV B, hyaluronoglucosaminidase activity, hexosaminidase activity and CS/DS degradation. The *Chromatin* mapping and the *Standard* pipeline did not produce significant pathways. The *Positional + eQTL + Chromatin* analysis produced 71 significant pathways (Supplementary Table 2), involving the neighborhoods *HYAL3*, *HYAL1*, *HYAL2* and *PLA2G2E*, *PLA2G5*, *PLA2G2C*.

The proposed pipeline increases robustness

We studied whether our proposed pipeline led to more robust results. For that purpose, we ran the whole protocol again on a random subset of the data containing 80% of the samples. We repeated this experiment 10 times for each SNP-gene mapping. In each subset, 49% of the individuals were cases, respecting the initial proportion of cases and controls of the entire dataset. Conservatively, we used the same SNP and gene significance thresholds as for the corresponding entire dataset.

The *Standard* pipeline, which does not include Biofilter network-information, produced on average 11.4 significant gene models (standard error (SE) 1.1). With the *eQTL* (respectively *Chromatin*) analysis, we detected on average 5.8 gene pairs (respectively 3.2) with SE 0.1 (respectively 0.4). With the *Positional + eQTL + Chromatin* mappings process, we detected on average 8.6 gene pairs with SE 1.3.

Fig 6 shows that pipelines including biological knowledge recover more than 60% of the gene pairs detected with the entire cohort, on average, (83% for *eQTL*, 60% for *Chromatin* mapping and 64% for *Positional + eQTL + Chromatin*), whereas without this knowledge (*Standard*), we recover less than 40% of the pairs. Hence, the *Standard* analysis appears to be the less robust in terms of conservation of gene pairs. This shows that filtering does increase robustness at the gene level.

Tissue-specific mappings do not recover many new interactions

To analyze the impact of tissue-specific mappings, we ran two analyses using exclusively eQTL and Chromatin mappings obtained from relevant tissue types. Specifically, we used mappings obtained from organs and tissues from the nervous and digestive systems (Supplementary Table 3). While the tissue-specific Chromatin analysis did not produce any significant gene pair, the tissue-specific eQTL analysis produced 4 significant gene pairs (Supplementary Fig 4). Nonetheless, only one is novel with regards to the (organism-wide) *eQTL* analysis: *IL18RAP*-*IL18R1*.

Discussion

In this article we proposed a new protocol for epistasis detection, based on a variety of functional filtering strategies, and studied its application to GWAS data for Inflammatory Bowel Disease. The protocol included several components to control for type I error, hereby strengthening our belief in the discovered genetic interactions.

A common theme in the interpretation of epistasis results consists on linking the associated variants to an altered gene function. In this article, we considered 3 different such SNP-gene mappings. Notably, the number of SNP-gene correspondences provided by each mapping differed by orders of magnitude. Moreover, the different mappings unevenly described genes; for instance, genes that had most SNPs mapped by using a chromatin contact map, had comparatively few eQTL SNPs. This motivated combining multiple mappings into an analysis (*Positional + eQTL + Chromatin*) in order to combine different perspectives of the epistasis process. For the most part, this complementary approach improved the analysis, by recovering most of the interactions significant in the analyses that used one mapping at a time. Importantly, our results display the benefits of going beyond one single SNP-gene mapping (often, genomic position) to interpret epistasis results. To our surprise, tissue-specific analyses using exclusively eQTL and Chromatin mappings from tissues related to IBD resulted in less significant gene pairs. Despite this setback, we believe that more targeted analyses (e.g. using only interactions from open chromatin in relevant cell-types) might lead to novel discoveries.

Restricting the tested interactions to functionally plausible pairs of genes and SNPs joins two faces of epistasis: searching for statistical epistasis, yet exclusively on plausible candidates for biological epistasis. This has several advantages. First, a more targeted input dataset reduces the number of tests and, in consequence, the multiple testing burden. In contrast, the high dimensionality of GWAIS data requires a much more stringent multiple testing correction and limits the detection of epistasis with low effect sizes. Adopting one of the proposed analyses may reduce the number of SNP interactions to test by more than half (Fig 1). Yet, the *Standard* analysis, which does not use Biofilter, produced the most significant gene models. Second, the proposed protocol addresses the robustness issues widespread in GWAIS by producing results that are consistent at the gene and pathway levels (Fig 5). Indeed, we observed an increased analytic robustness when using Biofilter gene models, in line with previous reports [32]. In particular, *eQTL* and *Chromatin* mappings increased said robustness. Third, restricting the search for epistasis to biologically plausible interactions yields results that are biologically interpretable and strikingly different from the ones obtained without using functional filtering (Fig 2). Not surprisingly, different mappings also provided very different interaction signals and give resolution of

information on different genes. In particular, we corroborated that the significant gene models from different functional filters were relevant to the biology of IBD. This was especially true for the *Chromatin* analysis (but also the *eQTL* analysis), giving rise to interactions with seemingly meaningful biological underpinnings, and stressing the relevance of regulatory variants in susceptibility to IBD. In contrast, the *Standard* analysis detected multiple interactions that were hard to interpret. For instance, several interactions involved RNA genes of unknown function (e.g. *LOC101927272* or *LINC02178*).

Remarkably, while the *Standard* analysis produced rich results, the *Positional* analysis did not lead to any significant SNP model. They both use genomic position to map SNPs to genes, but *Positional* is restricted to gene models in Biofilter. We note that the *Positional* analysis does not coincide with how Biofilter is typically used on GWAS data for epistasis detection. The latter involves pooling all SNPs that are mapped to genes which occur in Biofilter proposed gene interaction models, and subsequently exhaustive screening those SNPs for pairwise interactions. These pairs may also involve gene pairs that were not highlighted by Biofilter, in contrast to our *Positional* analysis. We evaluated the impact of Biofilter on the final results. No significant SNP interaction were detected in the *Positional* analysis. In the analysis without biofiltering (dataset reduced to mappable SNPs using genomic proximity, but not reduced to Biofilter gene pairs), 62 pairs were significant. Also, on the 86 SNP interactions that passed the experimental threshold in the *Standard* analysis (dataset not reduced to mappable using genomic proximity, nor Biofilter gene pairs), only 57 are mappable to gene interactions using genomic proximity. Hence, 66% of significant SNP pairs are mappable via genomic proximity in the *Standard* analysis.

An important component of our protocol is the conversion of SNP-based tests to gene-based tests. The most popular approach consists in aggregating SNP-level P-values into gene-level statistics, which can be done in different ways (see Ma et al. [33] for some early examples, and Vsevolozhskaya et al. [34] for recent developments). Here, we developed a generic approach that exploits a permutation strategy to define a P-value cutoff for SNP interactions, at a FWER of 5%, and then we followed the original implementation of the adaptive TPM (ATPM) to accommodate several truncation thresholds at once [19] while taking permutations instead of bootstrap as in Yu et al. [35]. The two algorithms are very similar, but we favored the TPM over the rank truncated product method of Yu et al. [35] that employs the product of the L most significant P-values, because the TPM only requires P-values smaller than a specified threshold, which is in line with the output of PLINK epistasis detection and saves storage space. Following both protocols and the recommendation of Becker and Knapp [36] we included measures derived from observed data in computing statistics under the null.

Remarkably, our proposed procedure keeps type I error under control, without additional corrections for multiple testing at the gene model. We hypothesize that this stems from two reasons. First, we apply a stringent correction for multiple testing at the SNP level. Second, when moving from SNP model significance to gene model significance, the ATPM only considers gene models that map to at least one significant SNP model. However, alternative strategies could have been considered. For instance, not restricting ourselves to significant SNP models, hence conducting ATPM on all gene models. This could have led to increased discovery, in cases where the SNP models mapped to a gene tend to be low, albeit non-significant. However, it may also lead to an increased type I error. Accounting for that would require a multiple test correction at the gene level. In turn, such correction would be difficult since the dependency between the tests is unknown. Additionally, in common multiple test correction procedures this would require a much higher number of permutations, in order to obtain the necessary numerical precision.

How to best perform a pathway analysis of epistasis results is understudied. Often, all genes belonging to any significant gene pair are simply pooled together into a joint enrichment analysis. This approach discards the gene-gene interaction information that was, indeed, the object of analysis. Hence, in our procedure we adapted the *Network neighborhood search* protocol from Yip et al. [37], which considers the topology of the network using the shortest paths between the studied genes. It should be noted that we only used the topology to derive a neighborhood for each significant gene pair; then, we discarded the edge information. Yet, there are several directions for improvement. One is to exploit the topology of the epistasis network beyond the creation of a neighborhood. Another one is to take into account the gene size (or the number of SNPs per gene), for instance by performing a weighted version of the statistical test. Jia et al. [38] suggested a method for gene set enrichment analysis of GWAS data, adjusting the gene length bias or the number of SNP per gene. In our data, we did observe a link between the significance of the gene models and the number of SNPs mapped to the gene. For instance, in the *eQTL* analysis, the only one producing significant pathways, the median number of SNPs per genes is 385 among genes in significant pairs, versus 3 SNPs/gene genome-wide.

Several protocol changes may impact final results. As reported elsewhere [32], these changes or choices include the modelling framework (parametric, non-parametric, semi-parametric), encoding of the genetic markers, as well as LD handling. In this work, we used an additive encoding scheme (0, 1, 2 indicating the number of copies of the minor SNP allele), a popular choice in part because of its computational efficiency. However, this encoding schemes has been reported to tend to increase false positives (for instance [39]). This observation is based on type I error studies with data generated under the null hypothesis of no pairwise genetic interactions but in the presence of main effects (see for instance [40]). Here, we investigated the type I error control of our protocols under a general null hypothesis of no genetic associations with the trait (no interactions and no main effects) and established adequate control. As a consequence, this does not guarantee that our generated SNP interaction results were not overly-optimistic. To this end, we adjusted SNP-level epistasis P-values for main effects as comprised in a polygenic risk score. Not only does such a post-analysis adjustment via conditional regression reduce over-optimism due to inadequate control for lower-order effects, thus addressing phantom epistasis [24], but it may also occasionally highlight the masking of SNP interactions (as was shown in Fig 4B - *eQTL*). More work is needed to investigate the impact on gene-level interaction results, derived accordingly. For convenience, we used the regression framework to identify SNP interactions and relied on earlier recommendations regarding LD handling [41].

Our protocols are built on output from Biofilter, that can be presented as a co-functional gene network. One of the motivations was its proven ability to highlight meaningful interactions in a narrower alternative hypothesis space, at the expense of leaving parts of the interaction search space unexplored. The database that Biofilter built contained 37 266 interactions. This is notably smaller than other gene interaction databases, like HINT [42], 173 797 interactions), or STRING [43], 11 759 455 interactions). Testing gene interactions with other (combinations of) biological interaction networks was beyond the scope of this paper. Furthermore, Biofilter analysis or exhaustive screening may lead to non-overlapping results. An example within a regression context is given in [32].

Potential implications

In this study we presented a protocol to enhance the interpretation of epistasis screening from GWAS. It includes gene-level epistasis discoveries with type I error under control, as well as a network-guided pathway analysis. Moreover, it improves the robustness of the results. While SNP pairs from a GWAIS study rarely replicate in other cohorts and arrays, results at the gene and pathway level are more likely to be reproducible. This can be achieved directly by applying the proposed protocol, or by testing SNP models in a cohort obtained from the gene pairs and pathways significant in other studies, via the SNP-gene mapping of interest. Aggregating SNP-level results into gene-level epistasis is challenging, but allows to include information from biological interaction databases. Based on that, we conducted multiple analyses that use different sources of prior biological knowledge about SNP-to-gene relationships and gene interaction models, as well as rigorous statistical approaches to assess significance. Each of them offers a different, albeit complementary view of the disease, which leads to additional insights.

Their application to GWAS data for inflammatory bowel disease highlighted the potential of our strategy, including network-guided pathway analysis, as it recovered known aspects of IBD while capturing relevant and previously unreported features of its genetic architecture. These strategies will contribute to identify gene-level interactions from SNP data for complex diseases, and to enhance our belief in these findings.

Methods

Gene interaction detection procedure

As we describe in more detail below, we applied different functional filters to the available data. These filters use plausible interactions between genes, and three different ways of mapping SNPs to those genes, and hence, to these interactions. These three mappings exploit different degrees of biological knowledge to map SNPs to genes, referred to as *Positional*, *eQTL* and *Chromatin*. For each of the three SNP-to-gene mappings, we only analyzed the pairs of SNPs corresponding to a gene pair with prior evidence for interaction. Across this article, we compared our findings in these scenarios to a *Standard* scenario. In this case, we exhaustively search for epistasis between all 38 225 SNPs that passed quality control (Table 4). We mapped the resulting significant SNP interactions to potential gene interactions using the positional mapping. An overview of the entire pipeline is presented in Fig 7.

From gene models to SNP models

Although the unit of analysis in GWAIS is the SNP, biological interactions are often characterized at the gene level. Hence, we mapped all SNPs in the dataset to genes using FUMA [44], a post-GWAS annotation tool. We created an artificial input where every SNP is significant in order to perform such mapping on all the SNPs. We performed three SNP-gene mappings using FUMA's SNP2GENE: positional, eQTL and 3D chromatin interaction (Table 4). In the *Positional* mapping, we mapped a SNP to a gene when the genomic coordinates of former was within the boundaries of the latter $\pm$ 10 kb. The *eQTL* mapping uses eQTLs obtained from GTEx [45]. We mapped an eQTL SNP to its target gene when the association P-value was significant in any tissue (FDR < 0.05). Lastly, in the *Chromatin* mapping, we mapped a SNP to a gene when a contact had been observed between the former and the region around the latter's promoter in the 3D structure of the genome (250 bp upstream and 500 bp downstream from the transcription start site) in any of the Hi-C datasets included in FUMA (FDR < 10^{-6}). This mapping might contain new, undiscovered, regulatory variants which, as for SNPs obtained through eQTL mapping, regulate the expression of a gene.

Co-function gene and SNP networks

We used Biofilter 2.4 [18] to obtain candidate gene pairs to investigate for epistasis evidence. Biofilter generates pairs of genes susceptible to interact (*gene models*) with evidence of co-function across multiple publicly available biological databases. It includes genomic locations of SNPs and genes, as well as known relationships among genes and proteins such as interaction pairs, pathways and ontological categories, but does not use trait information. As per Biofilter's default, we used gene models supported by evidence in at least 2 databases. Additionally, we removed self-interactions, as detection of within-gene epistasis requires special considerations and is beyond the scope of this paper.

Given this set of gene models, and three different ways of obtaining *SNP models* from it, we removed all the SNPs that did not participate in any SNP model. Subsequently, we created eight datasets. In one dataset no filter was applied (*Standard* analysis), i.e. no Biofiltering nor any SNP-to-gene mapping. Hence, the original SNP set was used. We also created one dataset exclusively for each SNP-to-gene mapping (*Positional*, *eQTL* and *Chromatin*). Lastly, we created four datasets using joint mappings: one with all the mappings (*Positional + eQTL + Chromatin*); and three with only two of them (*eQTL + Chromatin*, *Positional + eQTL* and *Positional + Chromatin*).

We discarded SNP models involving rare variants (MAF < 5%) or in Hardy–Weinberg equilibrium (P-value < 0.001). Regardless, all risk SNPs described in Liu et al. [46] were included, even when the aforementioned epistasis quality controls criteria did not hold up. Then, when the two SNPs of a *SNP model* were located in the HLA region, we discarded the pair, as it is difficult to differentiate between main and non-additive effects in this region [47]. Lastly, we discarded models where the SNPs were in linkage equilibrium (r^2 > 0.75), as motivated in Gusareva and Van Steen [41].

SNP-level epistasis detection and multiple testing correction

We used PLINK 1.9 to detect epistasis through a linear regression on the population structure adjusted phenotypes with the option `-epistasis`:

$$Y = \beta_0 + \beta_1 g_A + \beta_2 g_B + \beta_3 g_A g_B, \quad (1)$$

where g_A and g_B are the genotypes under additive encoding for SNPs A and B respectively, Y is the adjusted phenotype, and β_0, β_1, β_2, and β_3 are the regression coefficients. PLINK performs a statistical test to evaluate whether $\beta_3 \neq 0$. It only returns SNP pairs with a P-value lower than a specified threshold. We used the default 0.0001. Only SNP models were considered, apart from the *Standard* approach.

To correctly account for multiple testing, the P-value threshold of significance had to be dataset-dependent as the number of tested SNP pairs changed from dataset to dataset. We obtained these thresholds through permutations as in Hemani et al. [48] (Fig 7). More specifically, for each dataset, we permuted the phenotypes 400 times and fitted the aforementioned regression-based association model. This produced a null distribution of the extreme P-values for this number of tests given the LD structure in the data. For each dataset, we took the most extreme P-value from each of the 400 permutations and set the threshold for 5% family-wise error rate (FWER) to be the 5% percentile of these most extreme P-values. Posterior experiments showed that a higher number of permu-

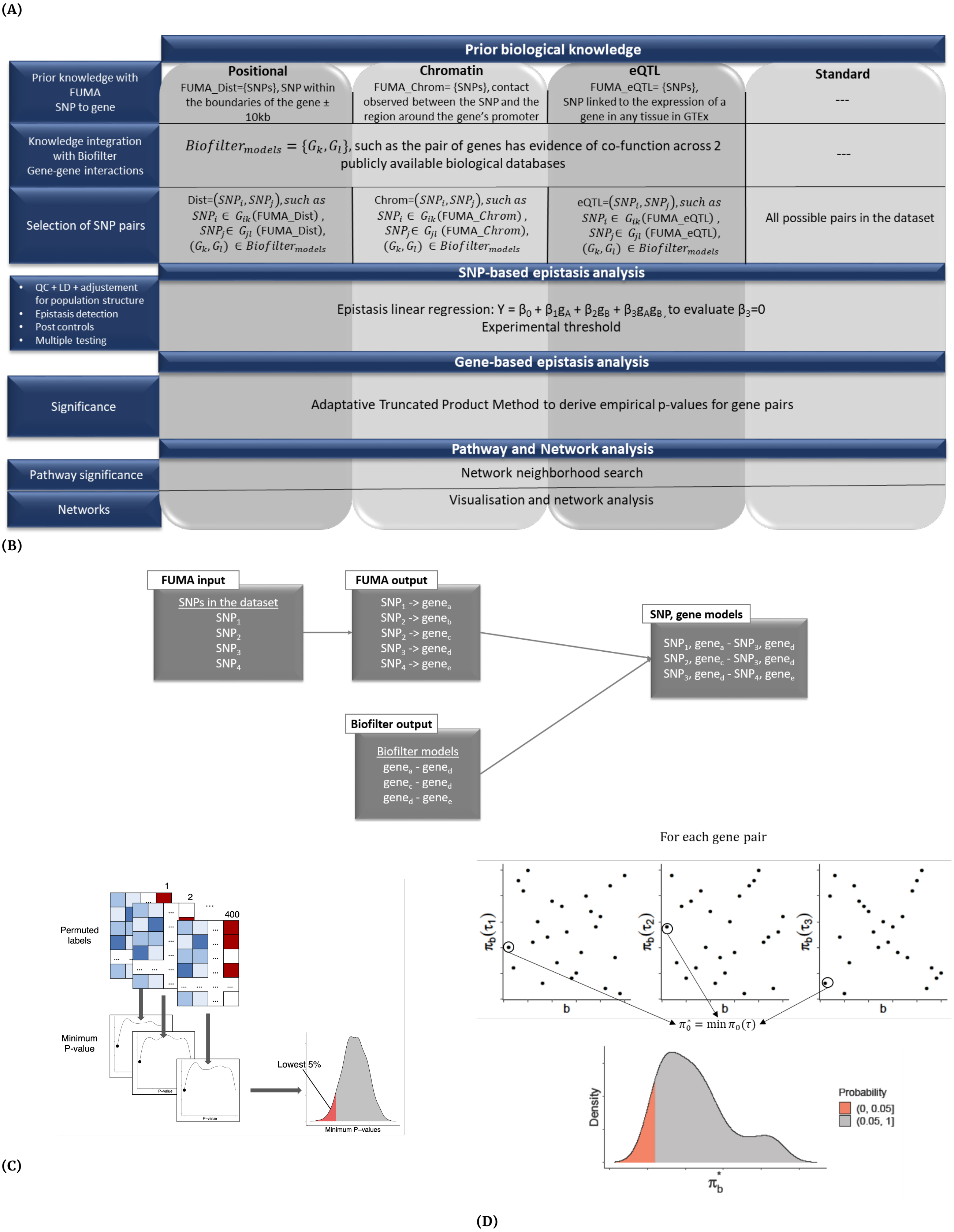

Figure 7. **(A)** Overview of the investigated gene-gene interaction detection protocols, described in *Gene interaction detection procedure*. **(B)** Summary of the procedure to obtain SNP and gene models using FUMA and Biofilter, described in *Co-function gene and SNP networks*. **(C)** Permutation procedure to obtain the SNP model P-value threshold, described in *SNP-level epistasis detection and multiple testing correction*. **(D)** Overview of the adaptive truncated product methodology, described in *From SNP-level to gene-level epistasis*.

Table 4. Properties of the different SNP-gene mappings and the filtered datasets. We show the empirical threshold of significance obtained through permutation, and the corresponding Bonferroni threshold for comparison.

Analysis	Mappings	SNP models (SNPs)	Empirical	Bonferroni[1]
Standard	-	7.3×10^8 (38 225)	1.1×10^{-10}	6.9×10^{-11}
Positional	1.7×10^5	3.0×10^5 (16 417)	1.6×10^{-7}	1.7×10^{-7}
Chromatin	2.4×10^6	6.0×10^6 (30 146)	1.0×10^{-8}	8.3×10^{-9}
eQTL	4.1×10^5	1.2×10^6 (16 652)	6.2×10^{-8}	4.0×10^{-8}
eQTL + Chromatin	2.7×10^6	9.0×10^6 (33 419)	6.5×10^{-9}	5.6×10^{-9}
Positional + eQTL	5.3×10^5	1.7×10^6 (23 642)	3.9×10^{-8}	2.9×10^{-8}
Positional + Chromatin	2.5×10^6	7×10^6 (33 195)	8.2×10^{-9}	7.1×10^{-9}
Positional + eQTL + Chromatin	2.8×10^6	9.6×10^6 (34 548)	5.0×10^{-9}	5.2×10^{-9}

[1]Bonferroni threshold based on the number of possible SNP pairs in the analysis.

tations, 1 000, barely changed the empirical threshold (data not shown). Hence, 400 was a sufficient number of permutations to obtain an adequate threshold.

From SNP-level to gene-level epistasis

Our next step was to use significant SNP interactions to identify significant gene interactions, which requires combining the P-values of all SNP pairs mapped to the same gene pair. Suppose that SNP interaction tests have been conducted for N individual hypotheses $H_{0i}, i = 1, 2, \ldots, N$, for example, N SNP models mapped to the same gene model. We tested the joint null hypothesis $H_0 = \bigcap_{i=1}^{n} H_{0i}$ at significance level α versus the combined alternative hypothesis H_1: at least one of H_{0i} is false. To do so, we considered all SNP pairs mapped to the same gene pair as a set of tests with the same global null hypothesis, and applied the Adaptive Truncated Product Method (ATPM) [19] (Fig 7).

ATPM is an adaptive variant of the Truncated Product Method (TPM) of Zaykin et al. [49], which uses as a statistic the product of the P-values smaller than some pre-specified threshold (here, significant SNP interactions) tests. More specifically, given a truncation point τ and a number N of significant SNP interactions, this test statistic is given as $W(\tau) = \prod_{i=1}^{N} p_i^{I(p_i \leq \tau)}$ where $I(\cdot)$ is the indicator function. TPM is interesting in our context because it does not require P-values for all SNP pairs but only for the most strongly associated ones.

The distribution of $W(\tau)$ under the null hypothesis is unknown when the individual tests are not independent, which is clearly the case here, but an empirical P-value $\hat{\pi}(\tau)$ can be estimated through permutations. Because the choice of τ is arbitrary, the adaptive version of TPM (ATPM) explores several values of τ and selects $\hat{\pi}^* = \min_\tau \hat{\pi}(\tau)$. The distribution of $\hat{\pi}^*$ under the null hypothesis can again be determined through permutations [50].

In our procedure, which is detailed below for a given gene pair, we used $B = 999$ permutations and $\tau \in \{0.001, 0.01, 0.05\}$. Remarkably, and following the suggestion of Becker and Knapp [36], the null distribution includes both the statistic from the observed dataset, and from the 999 permutations.

i. For each SNP model $i = 1, \ldots, N$ mapped to the gene pair, compute its P-values $p_{i,b}$ in the original dataset ($b = 0$) and for each of the $B = 999$ permutations ($b = 1, \ldots, B$).
ii. For each value of τ and b, compute the test statistic $W(\tau)$.
iii. For each value of τ and b, estimate the P-value : $\pi_b(\tau) = \frac{\sum_{l=0}^{B} I(W_b(\tau) \geq W_l(\tau))}{B+1}$.
iv. For each value of b, compute $\pi_b^* = min_\tau \pi_b(\tau)$.
v. Estimate the P-value of the gene model as $P_0 = \frac{\sum_{l=0}^{B} I(\pi_0^* \geq \pi_l^*)}{B+1}$.
vi. Reject the global null hypothesis if $P_0 \leq \alpha = 0.05$.

Studying the impact of confounding main effects

The SNPs from some detected interactions were near SNPs with main effects. To assess the impact on the results, we studied the difference between β_3 in Eq. 1 and in the following model:

$$Y = \beta_0 + \beta_1 g_A + \beta_2 g_B + \beta_3 g_A g_B + \beta_4 \text{PRS}.$$

PRS is the polygenic risk score (PRS) computed for the sample. We expect the PRS to capture the variance explained by main all effects.

We computed the PRS with PRSice-2 [51], using the default options. Since it requires GWAS summary statistics, we used PLINK `-assoc` to compute the association of each SNP in the original dataset (130 071 SNPs and 66 280 individuals, with the trait adjusted for PCs). Since the adjusted phenotype is quantitative, PLINK computes the linear regression coefficients and assesses their significance using the Wald test. PRSice performs clumping to remove SNPs that are in LD with each other. The r^2 values computed by PRSice are based on maximum likelihood haplotype frequency estimates. From the 130 071 initial variants, 28 389 variants remained after clumping (`-clump-kb 250kb, -clump-p 1, -clump-r2 0.1`). We used the average effect size method to calculate the PRS, with high-resolution scoring.

Pathway analysis

A pathway enrichment analysis on the neighborhood of a significant gene model can inform about the broader framework in which gene epistasis occurs. To define such neighborhoods, we adapted the network neighborhood search protocol from Yip et al. [37]. We computed the neighborhood of two genes as the list of all genes that (1) participate in any of the shortest paths between the two studied genes in the Biofilter network, once the direct link between them is removed; and (2) are also involved in a significant interaction with at least one other gene on these paths. We restricted our attention to neighborhoods containing at least 3 genes, including the 2 from the considered gene model. For each of these, we conducted a gene set enrichment analysis in all human gene sets from the Molecular Signature Database (MSigDB version 7) [52, 53]. We performed the enrichment analysis using a hypergeometric test, which compares the obtained overlap between two sets to the expected overlap from taking equally-sized random sets from the universe of genes. We favored the hypergeometric test over the chi-square test used in Yip et al. [37] because the sample sizes of the neighborhoods were small and because chi-square is an approximation whereas the hypergeometric test is an exact test. The universe set was analysis dependant. It contained all genes in an annotated pathway and that can be mapped via

genomic proximity to a SNP of the dataset for the *Standard* analysis, and genes present in Biofilter gene models, in an annotated pathway and that can be mapped via the appropriate SNP to gene mapping to a SNP of the dataset for the other analysis. Finally, pathways were said to be significant when the corresponding test P-value was lower than the Bonferroni threshold (0.05/(# pathways $\times$ # tested gene neighborhoods)), with *pathways* corresponding to pathways containing at least one gene of the neighborhood.

Availability of source code and requirements

- Project name: `network_epistasis.nf`
- Project home page: https://github.com/hclimente/gwas-tools
- Operating system(s): Platform independent
- Programming language: nextflow
- Other requirements: Bash, nextflow, PLINK 1.9 and R 4.0 or higher, with the following packages: clusterProfiler, data.table, igraph, msigdbr, snpStats, tidyverse.
- License: GNU GPL v3.0

Additionally, the code necessary to reproduce this article's results and analyses is available on GitHub at https://github.com/DianeDuroux/BiologicalEpistasis.

Data Availability

The data set supporting the results of this article is available upon request from the International Inflammatory Bowel Disease Genetics Consortium (https://www.ibdgenetics.org/). GWAS summary statistics are publicly available.

Declarations

List of abbreviations

ATPM: adaptive truncated product method; eQTL: expression quantitative trait loci; FWER: family-wise error rate; GWAS: genome-wide association study; GWAIS: genome-wide association interaction study; IBD: inflammatory bowel disease; LD: linkage disequilibrium; SNP: single-nucleotide polymorphism; TPM: truncated product method.

Ethical Approval

Not applicable.

Consent for publication

Not applicable.

Competing interests

The authors declare that they have no competing interests.

Funding

This project has received funding from the European Union's Horizon 2020 research and innovation programme under the Marie Sklodowska-Curie grant agreement No 666003 and 813533. Also from the RIKEN Special Postdoctoral Researcher Program. C-A.A. acknowledges funding from Agence Nationale de la Recherche (ANR-18-CE45-0021-01). Computational resources have been provided by the Consortium des Équipements de Calcul Intensif (CÉCI), funded by the Fonds de la Recherche Scientifique de Belgique (F.R.S.-FNRS) under Grant No. 2.5020.11 and by the Walloon Region. K.V.S. acknowledges opportunities and funding provided by WELBIO (Walloon Excellence in Life sciences and BIOtechnology).

Authors' contributions

Conceptualization: D.D., H.C.G., C-A.A., K.V.S.; data curation: D.D., H.C.G.; formal Analysis: D.D., H.C.G.; funding acquisition: C-A.A., K.V.S.; investigation: D.D., H.C.G.; methodology: D.D., K.V.S.; project administration: C-A.A., K.V.S.; resources: C-A.A., K.V.S.; software: D.D., H.C.G.; supervision: C-A.A., K.V.S.; validation: D.D., H.C.G.; visualization: D.D., H.C.G.; writing – original draft: D.D., H.C.G.; writing – review & editing: D.D., H.C.G.

Acknowledgments

We thank the International IBD Genetics Consortium for data collection and processing and for interesting discussions.

References

1. Buniello A, MacArthur JA, Cerezo M, Harris LW, Hayhurst J, Malangone C, et al. The NHGRI-EBI GWAS Catalog of published genome-wide association studies, targeted arrays and summary statistics 2019. Nucleic Acids Research 2019 Jan;47(D1):D1005–D1012. https://academic.oup.com/nar/article/47/D1/D1005/5184712, 00092.
2. Gordon H, Trier Moller F, Andersen V, Harbord M. Heritability in inflammatory bowel disease: from the first twin study to genome-wide association studies. Inflammatory bowel diseases 2015;21(6):1428–1434.
3. Ellinghaus D, Jostins L, Spain SL, Cortes A, Bethune J, Han B, et al. Analysis of five chronic inflammatory diseases identifies 27 new associations and highlights disease-specific patterns at shared loci. Nature genetics 2016;48(5):510.
4. Shaw KA, Cutler DJ, Okou D, Dodd A, Aronow BJ, Haberman Y, et al. Genetic variants and pathways implicated in a pediatric inflammatory bowel disease cohort. Genes & Immunity 2019;20(2):131.
5. Manolio TA, Collins FS, Cox NJ, Goldstein DB, Hindorff LA, Hunter DJ, et al. Finding the missing heritability of complex diseases. Nature 2009 Oct;461(7265):747–753. http://www.nature.com/articles/nature08494, 06874.
6. Lin Z, Wang Z, Hegarty JP, Lin TR, Wang Y, Deiling S, et al. Genetic association and epistatic interaction of the interleukin-10 signaling pathway in pediatric inflammatory bowel disease. World journal of gastroenterology 2017;23(27):4897.
7. Pedros C, Gaud G, Bernard I, Kassem S, Chabod M, Lagrange D, et al. An epistatic interaction between Themis1 and Vav1 modulates regulatory T cell function and inflammatory bowel disease development. The Journal of Immunology 2015;195(4):1608–1616.
8. Zhang J, Wei Z, Cardinale CJ, Gusareva ES, Steen KV, Sleiman P, et al. Multiple Epistasis Interactions Within MHC Are Associated With Ulcerative Colitis. Frontiers in genetics 2019;10:257.
9. Vermeire S, Rutgeerts P, Van Steen K, Joossens S, Claessens G, Pierik M, et al. Genome wide scan in a Flemish inflammatory bowel disease population: support for the

IBD4 locus, population heterogeneity, and epistasis. Gut 2004;53(7):980–986.
10. McGovern DP, Rotter JI, Mei L, Haritunians T, Landers C, Derkowski C, et al. Genetic Epistasis of IL23/IL17 Pathway Genes in Crohn's Disease Dermot. Inflammatory bowel diseases 2009;15(6):883–889.
11. Glas J, Stallhofer J, Ripke S, Wetzke M, Pfennig S, Klein W, et al. Novel genetic risk markers for ulcerative colitis in the IL2/IL21 region are in epistasis with IL23R and suggest a common genetic background for ulcerative colitis and celiac disease. The American journal of gastroenterology 2009;104(7):1737.
12. Moore JH, Williams SM. Traversing the conceptual divide between biological and statistical epistasis: systems biology and a more modern synthesis. BioEssays 2005 Jun;27(6):637–646. http://doi.wiley.com/10.1002/bies.20236, 00327.
13. Wu X, Dong H, Luo L, Zhu Y, Peng G, Reveille JD, et al. A novel statistic for genome-wide interaction analysis. PLoS genetics 2010;6(9):e1001131.
14. Lehne B, Lewis CM, Schlitt T. From SNPs to genes: disease association at the gene level. PloS one 2011;6(6):e20133.
15. Jorgenson E, S Witte J. A gene-centric approach to genome-wide association studies. Nature reviews Genetics 2006 12;7:885–91.
16. Niel C, Sinoquet C, Dina C, Rocheleau G. A survey about methods dedicated to epistasis detection. Frontiers in Genetics 2015 Sep;6. http://journal.frontiersin.org/Article/10.3389/fgene.2015.00285/abstract, 00000.
17. Gumpinger AC, Rieck B, Grimm DG, International Headache Genetics Consortium, Borgwardt K. Network-guided search for genetic heterogeneity between gene pairs. Bioinformatics 2020 Jun;p. btaa581. https://academic.oup.com/bioinformatics/advance-article/doi/10.1093/bioinformatics/btaa581/5861532, 00000.
18. Pendergrass SA, Frase A, Wallace J, Wolfe D, Katiyar N, Moore C, et al. Genomic analyses with biofilter 2.0: knowledge driven filtering, annotation, and model development. BioData Mining 2013 Dec;6(1). http://biodatamining.biomedcentral.com/articles/10.1186/1756-0381-6-25, 00042.
19. Sheng X, Yang J. An adaptive truncated product method for combining dependent p-values. Economics letters 2013;119(2):180–182.
20. Cortes A, Brown MA. Promise and pitfalls of the Immunochip. Arthritis Research & Therapy 2010;13(1):101. http://arthritis-research.biomedcentral.com/articles/10.1186/ar3204, 00451.
21. Ellinghaus D, Spain SL, Cortes A, Bethune J, Han B, Park YR, et al. Analysis of five chronic inflammatory diseases identifies 27 new associations and highlights disease-specific patterns at shared loci. Nature Genetics 2016 May;48(5):510–518. http://www.nature.com/articles/ng.3528, 00214.
22. Purcell S, Neale B, Todd-Brown K, Thomas L, Ferreira MA, Bender D, et al. PLINK: a tool set for whole-genome association and population-based linkage analyses. The American journal of human genetics 2007;81(3):559–575.
23. Piñero J, Ramírez-Anguita JM, Saüch-Pitarch J, Ronzano F, Centeno E, Sanz F, et al. The DisGeNET knowledge platform for disease genomics: 2019 update. Nucleic Acids Research 2019 Nov;https://doi.org/10.1093/nar/gkz1021.
24. de los Campos G, Sorensen DA, Toro MA. Imperfect Linkage Disequilibrium Generates Phantom Epistasis (& Perils of Big Data). G3: Genes, Genomes, Genetics 2019 Mar;9(5):1429–1436. https://doi.org/10.1534/g3.119.400101.
25. Lin Z, Hegarty JP, John G, Berg A, Wang Z, Sehgal R, et al. NOD2 mutations affect muramyl dipeptide stimulation of human B lymphocytes and interact with other IBD-associated genes. Digestive diseases and sciences 2013;58(9):2599–2607.
26. Beckly JB, Hancock L, Geremia A, Cummings FJR, Morris A, Cooney R, et al. Two-stage candidate gene study of chromosome 3p demonstrates an association between nonsynonymous variants in the MST1R gene and Crohn's disease. Inflammatory Bowel Diseases 2008 Apr;14(4):500–507. https://doi.org/10.1002/ibd.20365.
27. Burton PR, Clayton DG, Cardon LR, Craddock N, Deloukas P, Duncanson A, et al. Genome-wide association study of 14,000 cases of seven common diseases and 3,000 shared controls. Nature 2007 Jun;447(7145):661–678. https://doi.org/10.1038/nature05911, 00000.
28. Wu WKK, Sun R, Zuo T, Tian Y, Zeng Z, Ho J, et al. A novel susceptibility locus in MST1 and gene-gene interaction network for Crohn's disease in the Chinese population. Journal of Cellular and Molecular Medicine 2018 Feb;22(4):2368–2377. https://doi.org/10.1111/jcmm.13530.
29. Petrey AC, Carol A. The extracellular matrix in IBD: a dynamic mediator of inflammation. Current opinion in gastroenterology 2017;33(4):234.
30. Soroosh A, Albeiroti S, West GA, Willard B, Fiocchi C, Carol A. Crohn's disease fibroblasts overproduce the novel protein KIAA1199 to create proinflammatory hyaluronan fragments. Cellular and molecular gastroenterology and hepatology 2016;2(3):358–368.
31. Albeiroti S, Soroosh A, de la Motte CA. Hyaluronan's role in fibrosis: a pathogenic factor or a passive player? BioMed research international 2015;2015.
32. Bessonov K, Gusareva ES, Van Steen K. A cautionary note on the impact of protocol changes for genome-wide association SNP× SNP interaction studies: an example on ankylosing spondylitis. Human genetics 2015;134(7):761–773.
33. Ma L, Clark AG, Keinan A. Gene-based testing of interactions in association studies of quantitative traits. PLoS genetics 2013;9(2).
34. Vsevolozhskaya OA, Hu F, Zaykin DV. Detecting weak signals by combining small P-values in genetic association studies. Frontiers in genetics 2019;10:1051.
35. Yu K, Li Q, Bergen AW, Pfeiffer RM, Rosenberg PS, Caporaso N, et al. Pathway analysis by adaptive combination of P-values. Genetic Epidemiology: The Official Publication of the International Genetic Epidemiology Society 2009;33(8):700–709.
36. Becker T, Knapp M. A powerful strategy to account for multiple testing in the context of haplotype analysis. The American Journal of Human Genetics 2004;75(4):561–570.
37. Yip DKS, Chan LL, Pang IK, Jiang W, Tang NL, Yu W, et al. A network approach to exploring the functional basis of gene–gene epistatic interactions in disease susceptibility. Bioinformatics 2018;34(10):1741–1749.
38. Jia P, Wang L, Fanous AH, Chen X, Kendler KS, Zhao Z, et al. A bias-reducing pathway enrichment analysis of genome-wide association data confirmed association of the MHC region with schizophrenia. Journal of medical genetics 2012;49(2):96–103.
39. Van Steen K, Moore J. How to increase our belief in discovered statistical interactions via large-scale association studies? Human genetics 2019;138(4):293–305.
40. John JMM, Cattaert T, Van Lishout F, Gusareva ES, Van Steen K. Lower-order effects adjustment in quantitative traits model-based multifactor dimensionality reduction. PLoS One 2012;7(1).
41. Gusareva ES, Van Steen K. Practical aspects of genome-wide association interaction analysis. Human Genetics

2014 Nov;133(11):1343–1358. http://link.springer.com/10.1007/s00439-014-1480-y, 00015.
42. Das J, Yu H. HINT: High-quality protein interactomes and their applications in understanding human disease. BMC Systems Biology 2012;6(1):92. http://bmcsystbiol.biomedcentral.com/articles/10.1186/1752-0509-6-92, 00204.
43. Szklarczyk D, Gable AL, Lyon D, Junge A, Wyder S, Huerta-Cepas J, et al. STRING v11: protein–protein association networks with increased coverage, supporting functional discovery in genome-wide experimental datasets. Nucleic Acids Research 2019 Jan;47(D1):D607–D613. https://academic.oup.com/nar/article/47/D1/D607/5198476, 00072.
44. Watanabe K, Taskesen E, van Bochoven A, Posthuma D. Functional mapping and annotation of genetic associations with FUMA. Nature Communications 2017 Dec;8(1). http://www.nature.com/articles/s41467-017-01261-5, 00139.
45. GTEx Consortium. Genetic effects on gene expression across human tissues. Nature 2017 Oct;550(7675):204–213. http://www.nature.com/articles/nature24277, 00708.
46. Liu JZ, Van Sommeren S, Huang H, Ng SC, Alberts R, Takahashi A, et al. Association analyses identify 38 susceptibility loci for inflammatory bowel disease and highlight shared genetic risk across populations. Nature genetics 2015;47(9):979.
47. Traherne J. Human MHC architecture and evolution: implications for disease association studies. International journal of immunogenetics 2008;35(3):179–192.
48. Hemani G, Shakhbazov K, Westra HJ, Esko T, Henders AK, McRae AF, et al. Detection and replication of epistasis influencing transcription in humans. Nature 2014 Apr;508(7495):249–253. http://www.nature.com/articles/nature13005, 00162.
49. Zaykin DV, Zhivotovsky LA, Westfall PH, Weir BS. Truncated product method for combining P-values. Genetic Epidemiology: The Official Publication of the International Genetic Epidemiology Society 2002;22(2):170–185.
50. Ge Y, Dudoit S, Speed TP. Resampling-based multiple testing for microarray data analysis. Test 2003;12(1):1–77.
51. Choi SW, O'Reilly PF. PRSice-2: Polygenic Risk Score software for biobank-scale data. Gigascience 2019;8(7):giz082.
52. Subramanian A, Tamayo P, Mootha VK, Mukherjee S, Ebert BL, Gillette MA, et al. Gene set enrichment analysis: A knowledge-based approach for interpreting genome-wide expression profiles. Proceedings of the National Academy of Sciences 2005 Sep;102(43):15545–15550. https://doi.org/10.1073/pnas.0506580102.
53. Liberzon A, Birger C, Thorvaldsdóttir H, Ghandi M, Mesirov JP, Tamayo P. The Molecular Signatures Database Hallmark Gene Set Collection. Cell Systems 2015 Dec;1(6):417–425. https://doi.org/10.1016/j.cels.2015.12.004.

Click here to access/download
Supplementary Material
SNP2GENE_supplementary.pdf

Personal Cover
Click here to access/download;Personal Cover;SNP2GENE_letter.pdf

Dear Dr. Edmunds, dear reviewers,

We thank you for your consideration, attention to detail, and positive feedback on our manuscript. We have read and discussed the comments you raised with great interest and tried to address them in detail below (responses in blue). We believe this peer-review step has substantially improved the quality and comprehensiveness of the manuscript. We attach to this letter a final version of the article, and a marked version with all the changes.

We summarize here the main changes to the manuscript:

- We have adapted our manuscript to GigaScience's guidelines, including the appropriate sections when necessary. Notably, since GigaScience's articles do not number sections and subsections, we removed some of the cross-references to Sections and replaced others with named references or the most relevant Figure/Table.
- Moving the Methods after the Analysis section required adding context to some of the parts of the Analysis.
- We have included two additional families of analyses:
    - Tissue-specific eQTL and chromatin interactions
    - Positional + eQTL and Positional + chromatin analyses

  At the same time, we have removed the results about eQTL + Chromatin from the last sections of the article.
- We replaced all the networks layout for a circular one, which facilitates comparing them.
- We have replaced our claims about the reproducibility of the pipeline with the more accurate (as per our experiments) robustness.
- We have fixed typos and added clarifications.

Reviewer #1: Here, the authors presented a pipeline for the analysis of epistasis effects in GWAS data (GWAIS). This is traditionally a difficult problem due to the large search space involved, which makes the analysis computationally intensive and suffers from multiple testing, a new method that restrict search space can definitely help making GWAIS more feasible and reproducible. I have some questions after reading the current paper, please excuse me if the information is already presented within the paper:

1. I am not sure what the Standard model comprised of. According to the methodology section, the Standard analyzed all SNP pairs without prior filtering, does that mean all 14,501,130,150 SNP pairs (C(170301, 2)) were tested? Or was it not all SNP pairs were considered?

By Standard, we refer to what we see as a Standard pipeline to detect epistatic interactions: after quality control, we exhaustively search for significant epistatic interactions between 38,225 SNPs. In other words, all possible 7.3×10^8 possible interactions are evaluated. Then, we use positional mapping to assign significant SNP pairs to gene pairs.

We have improved our description at the beginning of the Methods, as well as added a brief description the first time we refer to it in the results to make it clearer.

2. When presenting the number of SNPs linked to each gene based on different criteria (e.g. position, eQTL or Chromatin contact), wouldn't the gene size be a major predictor of the number of SNPs link? It would most likely be the case for positional mapping, right?

This is indeed a valid concern, which could result in sampling hypotheses in a biased way. However, when we compared the size of the genes to the number of SNPs that we mapped to them, we do not find that more SNPs are mapped to longer genes:

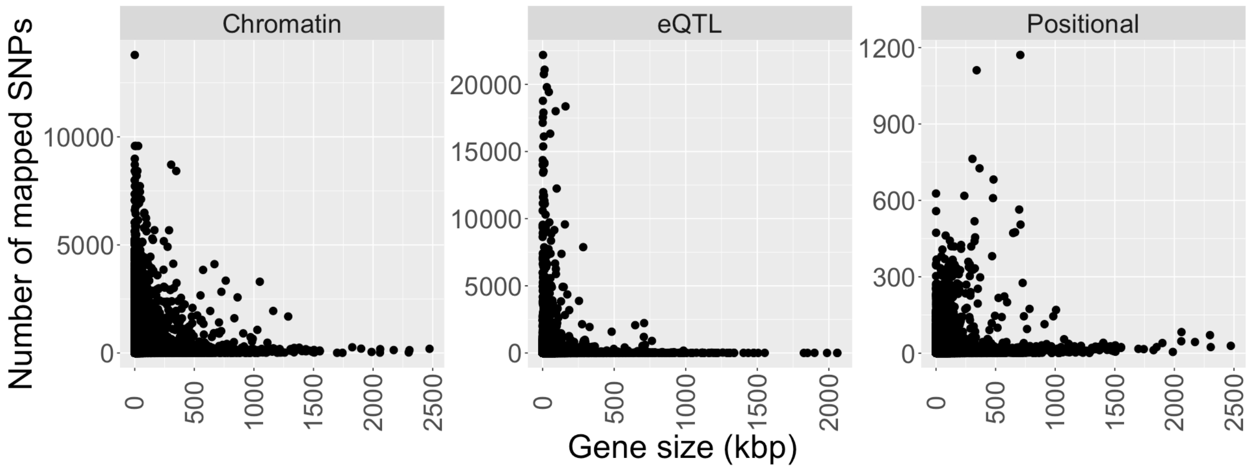

3. I am curious to see if restricting the eQTL and Chromatin information to disease specific tissue will help improving the performance of the current model.

Indeed, this is a relevant analysis. We found that tissue-specific eQTL results in a new significant gene pair; however, it misses 4 pairs that were significant organism-wide. With regards to the tissue-specific Chromatin protocol, it does not result in any new significant gene pair. For more details, see our answer to Reviewer #2's 3rd question.

4. Very little information was provided for the PRS analysis.

Thanks for pointing this out. We have included these details in the new Methods section "Studying the impact of confounding main effects".

What genome wide association summary statistics were used?

The genome-wide association summary statistics were obtained via PLINK option --*assoc* on the original dataset containing 130,071 SNPs and 66,280 individuals. Since the phenotype is quantitative, PLINK computes linear regression coefficients, which are used by PRSice, and assess their significance via a Wald test.

Did the authors perform high-resolution scoring?

Yes, since we did not change PRSice's default option.

With shrinkage / thresholding done in normal PRS analysis, some SNPs' effect might be excluded or "shrinked" away from the PRS model, would that affect the interpretation of the PRS covariate analysis? E.g. maybe SNPs not included in the PRS model were those unaffected? (With PRSice, can use --print-snp to obtain list of SNPs that are included in the model)

To calculate the PRS, the default options of PRSice were used. The 130,071 variants observed in the original file were included. By default, PRSice performs clumping to remove SNPs that are in LD with each other. The r^2 values computed by PRSice are based on maximum likelihood haplotype frequency estimates. A total of 28,389 variants remained after clumping (--clump-kb 250kb, --clump-p 1.000000, --clump-r2 0.100000). The average effect size method is used to calculate the PRS. We have included these details in the new Methods section "Studying the impact of confounding main effects".

To identify if SNPs not included in the PRS model were those unaffected, we performed GWAS with all 130,071 SNPs and the continuous trait as outcome: 5,658 SNPs pass the genome-wide significance threshold ($5x10^{-8}$). Among them 264 SNPs are also used in the PRS computation (~4%). PRS is supposed to capture polygenic effects, also those not included in the PRS model via existing LD patterns. So in the same way a PRS model is used to capture genome-wide effects for predictions in an independent dataset, we are using PRS to capture genome-wide effects to correct for in our epistasis runs.

5. It seems like Biofilter provide a SNP-SNP interaction prediction model, how does that compare to what was presented here?

In theory, that is the case, in practice we couldn't make Biofilter to compute them.

Biofilter conducts a simple positional mapping, based on the genomic coordinates of the SNP and the gene. Hence, it is equivalent to our Positional mapping, albeit probably with different parameters. Given gene-gene interactions, and a SNP-to-gene mapping, obtaining (potential) SNP-SNP interactions is a trivial step. It simply involves two *inner join* operations over two tables.

Hence, computing SNP-SNP models ourselves had two advantages. First, that there is no difference other than the choice of mapping between the eQTL and Chromatin pipelines (which Biofilter wouldn't compute) and the Positional one. Second, FUMA gave us great control over how to perform the SNP-gene positional mapping.

6. Figure 3, the results from eQTL + Chromatin and Position + eQTL + Chromatin is identical.

Although they are very similar, they are not exactly the same. The eQTL + Chromatin analysis detected 48 significant SNP pairs and 12 significant gene pairs, whereas the eQTL + Chromatin + positional analysis identified 45 SNP pairs and 13 gene pairs. The additional gene pair is *PLA2G2E-PLA2G2C*; we have added this information to the main text.

Additionally, we noticed that the graph layout made it hard to compare the networks. We have replaced all of them with a circular plot which, we believe, facilitates spotting differences between the analyses.

Together, it seems like the positional mapping does not contribute to the result at all, which is a bit surprising. Are there any explanation of that?

Indeed, both the lack of significant gene pairs in the Positional analysis and similarities between eQTL + Chromatin and Position + eQTL + Chromatin analyses point towards the little contribution of positional information. A partial explanation is that the positional mapping maps genes to fewer SNPs than the other mappings (Fig 1A). Our results seem to suggest that epistasis is particularly relevant to IBD via alterations in gene expression regulation, rather than other sorts of regulation captured by the positional mapping, like alternative splicing. This is reinforced by the fact that our Positional mapping only included SNPs within ±10 kbp of the gene coordinates. In contrast, other studies consider larger windows to capture potential cis-eQTLs (sometimes up to 2 Mbp).

Would it be due to mapping of the Immunochip array, or a characteristic of IBD?

Our primary suspect would be biology itself. The Immunochip was designed from regions deemed interesting in previous genetic studies on autoimmune and inflammatory diseases, regardless of their functional implications. Hence, we see no reason for the array design to have a big impact in this regard.

On the other hand, our results are consistent with broader findings in the GWAS community. Specifically, the observation that most GWAS findings fall outside the gene boundaries, and regulatory elements play an important role.

7. Given the sample size of the current data, a HWE threshold of 0.001 seems rather stringent. Will the result improve if a less stringent threshold is used (e.g. 1e-6?)

Indeed, we used a pretty conservative threshold with respect to common practice. Since one of our focuses was boosting reproducibility, we wanted to avoid false positives as much as possible. In any case, the amount of SNPs included in the analysis barely changes when we use a threshold of 10^{-6}:

	No filter	Positional	eQTL	Chromatin	eQTL + Chromatin	eQTL + Chromatin + Positional

HWE $P < 10^{-3}$	38,225	16,417	16,652	30,146	33,419	34,548
HWE $P < 10^{-6}$	38,667	16,606	16,834	30,460	33,779	34,889
Relative change (%)	1.2	1.2	1.1	1.0	1.1	1.0

Reviewer #2: This manuscript presents a workflow for gene-gene epistasis detection which leverages functional annotation resources such as Biofilter (to reduce the search space) and FUMA (to map SNPs to genes) and investigates the results obtained via different SNP-gene mapping criteria (positional, eQTL, Chromatin contacts, and some combinations of these). Moreover, these results are compared with those obtained via a 'standard' analysis where no filtering is applied to pair selection and positional SNP-gene mapping is used. Due to the challenges presented by GWAIS, leveraging functional genomics to focus the search is a valid strategy which has been employed in other recent works in the field. This is a nice work and the paper is generally well written, with sufficiently detailed methodological information. Below are some comments and questions.

1. As indicated in recent GWAS works aimed at 'solving GWAS loci' (i.e. determining the genes affected by significant SNPs), it is not always the case that the gene affected by a SNP is that positionally closest to the SNP. Indeed, a SNP may not only affect a gene when it resides in its coding or promoter regions, but it may also affect a gene when it resides in a far away enhancer. This is why epigenetic information such as chromatin loops (referred by the authors as 'Chromatin') can be useful for SNP-gene mapping. In the presence of chromatin contacts or eQTL information, typically one would use the derived mapping to augment the positional mapping, which is always available. That is, if one had chromatin contacts data, they would use positional + Chromatin to map SNPs to genes. If one had eQTL data, they would use positional + eQTL to map to genes. If one had both, they would use positional + eQTL + Chromatin. From a biological interpretability perspective, there is no reason to exclude the positional information.

For example, a SNP in the promoter of gene could interact with a SNP in a distal enhancer of another gene, affecting a specific trait. In view of this, the statement (lines 326-328) "Since the main objective of this protocol is to increase the biological interpretability of epistasis findings, we have excluded other combinations that mix functional and non-functional information (Positional + eQTL and Positional + Chromatin)" is not quite valid, as positional information is also functional.

On the other hand, using eQTL only, Chromatin only, or eQTL + Chromatin, albeit interesting in terms of looking at how this type of reduction in the search space affects results, do not quite reflect a biologically guided approach.

Indeed, one primary goal was to characterize the contribution of each source of information. To

not overwhelm the reader and due to the little contribution that the Positional mapping had in general, we decided to exclude Positional + eQTL and Positional + Chromatin analyses from the final manuscript. However, we recognize that this is information that might be of interest to some readers. We have included two rows corresponding to these analyses in Tables 1, 2 and 4, as well as the corresponding SNP and gene networks to Supplementary Fig 2 and Figure 2, respectively. Moreover, we have justified our exclusion from the rest of the manuscript to the reader in the section *Gene epistasis: "functional" mappings boost discovery and interpretability*. Moreover, for the same reasons, we have removed eQTL + Chromatin from the rest of the paper.

These are the newly included results:

	Positional + eQTL	Positional + Chromatin
SNP models (SNPs (after QC and biological filters)	1,739,908 (23,642)	7,025,632 (33,195)
Experimental threshold	3.85×10^{-8}	8.16×10^{-9}
Number of significant SNP pairs (number of SNPs)	76 (43)	39 (21)
Number of significant gene pairs (number of genes)	7 (11)	5 (10)
Number of significant pathways	16	0
Number of components in the gene network	5	5

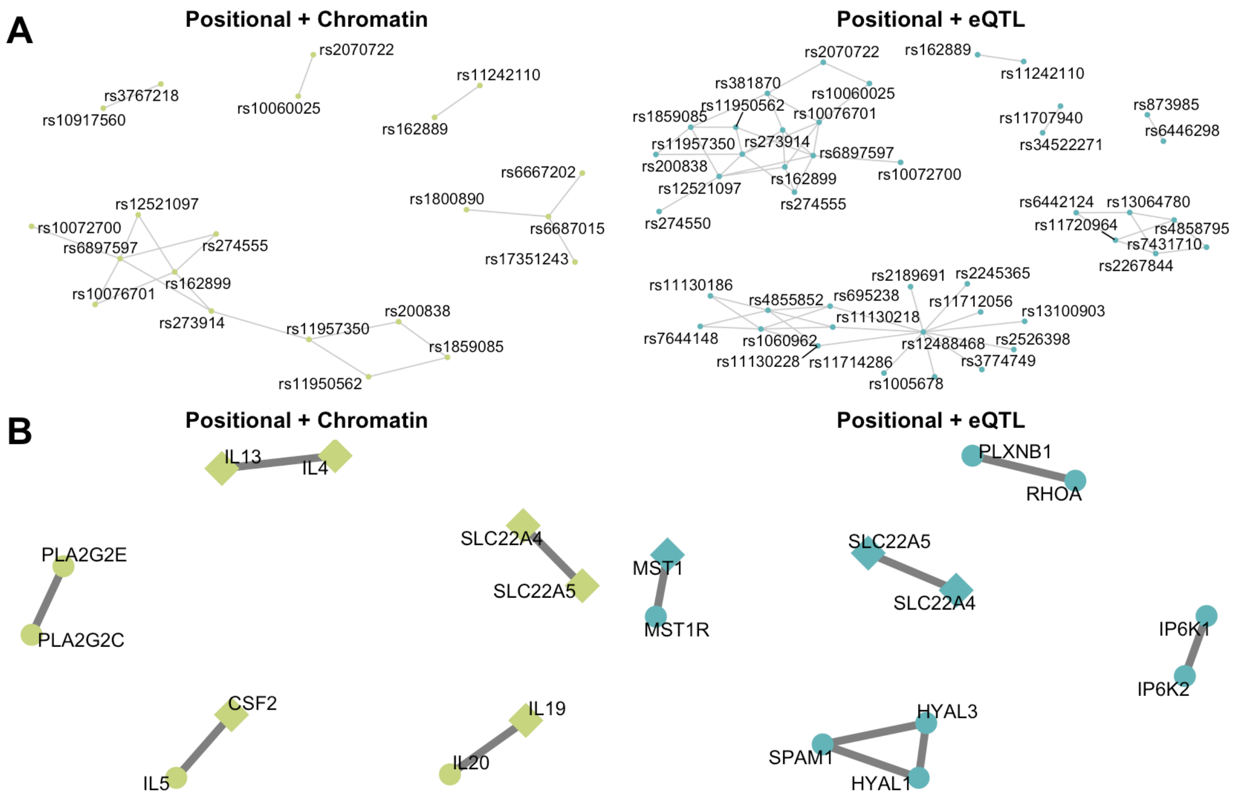

2. I wonder whether the authors have considered filtering also by markers of relevant chromatin states. Information about open chromatin and other epigenetic marks could help further filtering SNPs, both in enhancers and promoters. This would be particularly useful for SNPs mapped via chromatin contacts, which are likely to contain many irrelevant signals.

Indeed, we had considered this possibility. However, we were discouraged by the lack of new results when considering only tissue-specific chromatin interactions (see next answer). Since the relevant changes in chromatin state should be those happening in the tissues relevant to the disease, we hypothesized that we would attain similar (lack of) results if we restricted, even more, the number of gene pairs tested. Nonetheless, we believe this is a promising line of research, and we have modified the discussion to reflect that. However, we would need to overcome certain barriers first (e.g. larger sample size, a different phenotype, a more clear tissue candidate, etc.).

3. The eQTL and chromatin contact data used in this work were from all available tissues. Typically, GWAS related functional filtering is done using data from tissues relevant to the trait under investigation, when available. For IBD, it may help to restrict to intestinal tissues, immune cells (like T-cells, macrophages, dendritic cells), and possibly also nervous system cells (which, at least according to some, could also be among the potential 'culprit' IBD tissues).

Thanks for bringing this up. We conducted two additional analyses, for eQTL and Chromatin,

using exclusively mappings obtained from relevant tissues. We used eQTLs and Chromatin mappings from a set of tissues from the digestive and nervous systems (see full list below). These are the results:

	eQTL	Chromatin
Tissues	Colon Sigmoid,<br>Colon Transverse,<br>Esophagus Gastroesophageal Junction,<br>Esophagus Mucosa,<br>Esophagus Muscularis,<br>Pancreas,<br>Small Intestine Terminal Ileum,<br>Stomach,<br>Whole Blood,<br>aveALL,<br>Brain Anterior cingulate cortex BA24,<br>Brain Caudate basal ganglia,<br>Brain Cerebellar Hemisphere,<br>Brain Cerebellum,<br>Brain Cortex,<br>Brain Frontal Cortex BA9,<br>Brain Hippocampus,<br>Brain Hypothalamus,<br>Brain Nucleus accumbens basal ganglia,<br>Brain Putamen basal ganglia,<br>Brain Amygdala	Pancreas,<br>Small Bowel,<br>Orsolateral Prefrontal Cortex,<br>Hippocampus
SNP models (SNPs (after QC and biological filters)	3×10^5 (6,769)	4.6×10^3 (990)
Experimental threshold	7.893×10^{-7}	NA - no epistatic SNP-pair of the permutations is mappable a biofilter gene-pair
Number of significant SNP pairs (number of SNPs)	15 (15)	NA
Number of significant gene pairs (number of genes)	4 (8)	NA

Number of significant pathways	0	NA
Number of components in the gene network	4	NA

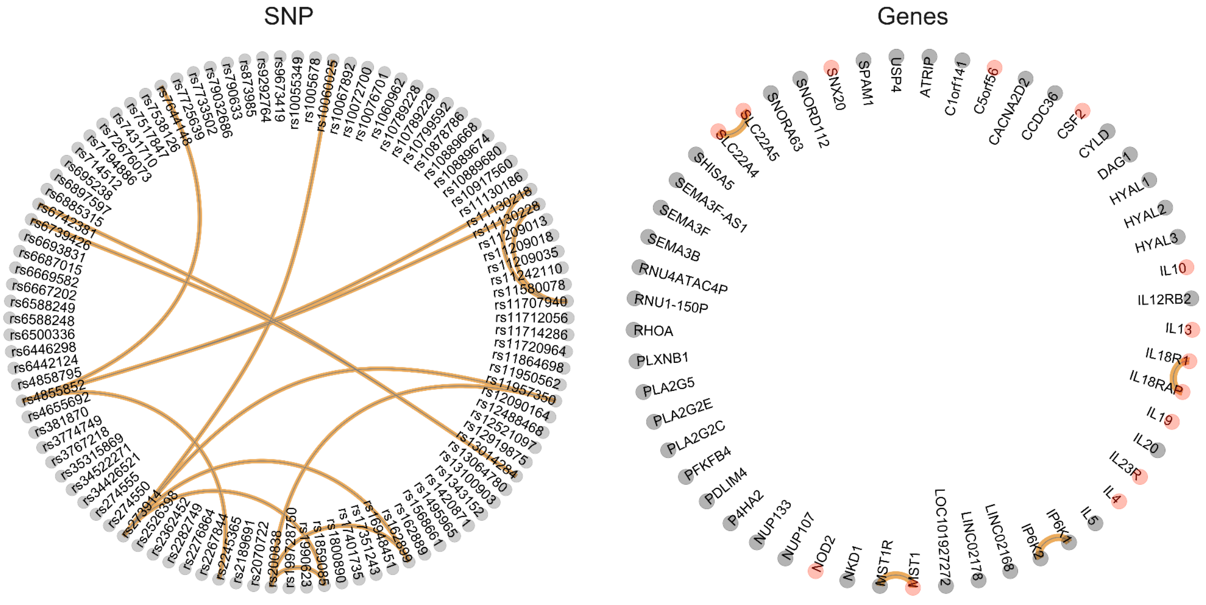

(We have included these results in the article, as Supplementary Table 3 and Supplementary Fig 4.)

The eQTL analysis results in a new gene pair (*IL18RAP-IL18R1*) and misses 4 pairs that were significant organism-wide (*SPAM1-HYAL1, HYAL1-HYAL3, HYAL3-SPAM1, PLXNB1-RHOA*), while the Chromatin analysis does not return any pair at all. This might be due to low statistical power, lack of relevant, cell type-specific information. We have added a short section in Results ("Tissue-specific mappings do not recover many new interactions") and a line in the Discussion to this effect.

4. To adjust for population structure the authors regressed out the first 7 PCs from the phenotype. Given that the PCs are confounders, it would be good to discuss the impact of doing this as opposed to also regress the confounders out of the SNPs, i.e. testing the response residuals vs the SNP residuals.

In this regard, our options were restricted by the inputs accepted by our tool of choice (PLINK), which did not take adjusted genotypes or additional, fixed confounders in the model.

In the same spirit, it would be good to discuss the impact of the PC-SNP association on the p-value and type-I error results obtained by permuting the response residuals.

Notably, adding PCs as fixed covariates is not an option for many epistasis methodologies. It was also not considered due to the computational burden. We acknowledge that pragmatic choices may be suboptimal. In particular, the Frisch-Waugh-Lovell theorem says that the multiple regression coefficient of any single variable can also be obtained by first netting out the effect of other variables in the regression model from both the dependent variable and the independent variable. It means that coefficients do not change if a model is fitted for adjusted trait versus adjusted predictors. However, this theorem relates to multiple linear regression whereas we are considering a binary phenotype. Also, as predictors are not adjusted for, the model parameter estimates for SNP-trait relationships are likely to be different. The latter has not diminished the popularity of the practice, when PCs cannot be entered into the regression model as covariates, especially when sample sizes are large enough and inference based on models with a logit or identity link function become more similar. Another motivation often finds its basis in type I error control: if we were to reshuffle the phenotypes and keep the confounders (PCs) and the SNPs untouched, then we would also break the link between the confounding variables capturing shared genetic ancestry or population structure and the trait. Thus, we would be generating a more global null of no genetic effect and no population structure. Lastly, when choosing not to account for confounders in the model formalism, an a posteriori correction needs to be carried out. Genomic control is one such approach which has been shown to be suboptimal over on-the-fly correction approaches in GWAS. The alternative is to encapsulate the entire analysis in a two-stage analysis design: stage 1 involves the analysis without correction for confounding; stage 2 involves only following up on hits with a proper model. Doing so may reduce power (as one is risking to follow up on overly-optimistic results) or offers additional challenges, such as type I error control and assessing the impact of mixed modeling paradigms across stages.

5. Section 2.1 is somewhat too concise and may result unclear to the reader. Later in the Discussion (lines 229-239) the authors explain how their procedure corrects for multiple testing at the SNP model without additional corrections for multiple testing at the gene model (this is also implicitly described in Fig 7CD), but yet their procedure keeps type I error under control. However, it may be beneficial, for ease of reading, to expand section 2.1 (via text and/or figure) so to clarify better, at the onset, where and when multiple testing corrections are applied.

We fully agree with this point. The presented type I error refers to significant gene interactions. To make it clearer, we have moved this section right after the section “Gene epistasis: ‘functional’ mappings boost discovery and interpretability”, in which we introduce the detected gene interactions. We have adapted the text to fit its new placement.

6. In the absence of a replication data set, the authors assess the robustness of the gene pair results via 10 repetitions of the workflow using 80% of the discovery data set. It would be useful to include some discussion of how their results could be further assessed in other GWAS data sets (e.g. from UK biobank, etc.), in view of the fact that it is typically hard to reproduce epistasis

findings, at least at the SNP level. Certainly one could first check whether the discovered SNP-SNP interactions are reproduced and limiting the analyses to those pairs would require a less severe multiple testing correction. But another approach may be to start with the discovered gene pairs and then analyze all pairs of SNPs mapping to these genes (not necessarily those discovered in this study), etc. Do the authors plan future follow-up studies on this?

One of the needs our study tried to address was the lack of replicability of epistasis results at the SNP level. However, by moving up to higher levels in biology (genes and pathways), we expect that the results become more replicable in different cohorts. Of course, the reverse process that you suggest is also promising: using significant pathways and genes identified in one study are used to propose SNP models in another study. We have included these considerations in Potential implications.

7. In section 2.7 the results of pathway analyses on 3 (eQTL, Positional, and Standard) of the 5 networks presented in Figure 3 are provided. What about the other 2?

We apologize for this oversight. We detected 39 significant pathways in the eQTL + Chromatin analysis. They all come from the neighbourhood formed by *HYAL3*, *HYAL1* and *HYAL2*. However, as per another comment, we have removed this analysis from most of the paper.

We identified 71 significant pathways for the eQTL + Chromatin + Positional analysis. They come from the two neighbourhoods formed by *HYAL3*, *HYAL1* and *HYAL2*, and *PLA2G2E*, *PLA2G5* and *PLA2G2C* respectively. We have included these results in “Pathway analyses highlight the involvement of the extracellular matrix in IBD”, and in Supplementary Table 2.

8. For these two points I defer to the editor:
(i) The format of the manuscript is close to but does not exactly match the specifications at https://academic.oup.com/gigascience//pages/research. I do not know how strict these specifications are and I have no objections to the current format.

Thanks for pointing this out. We have adapted our article to GigaScience’s specifications.

(ii) Data availability is not discussed (as per Data and materials in https://academic.oup.com/gigascience/pages/instructions_to_authors). I imagine that the IIBDGC only makes publicly available the summary statistics. This is, however, common in the GWAS field.

Indeed, that is the case. However, the consortium is open to new research proposals, in which case interested researchers could access the data. We have specified this in the new “Data availability” section.

9. Some minor notes follow:
(i) In the Author Summary the 'ATPM' acronym is used for the first time without explanation.

Since GigaScience’s articles do not include an “Author summary”, we have removed it. Instead, we distilled the main ideas of the “Author Summary” into the “Key Points”.

(ii) In section 4.2 it would be helpful to re-iterate that the SNP-gene mapping for the Standard analysis was genomic proximity (this is only mentioned briefly at line 206).

Thanks for pointing this out, indeed the Standard analysis is not well-defined. We have added a brief description at the beginning of (old) Section 4.2, as well as a brief description the first time we refer to it in the results.

(iii) Typo at line 168 "the same than" should be "the same as".

Thanks, we fixed the typo.

(iv) It should be specified which of the MigSigDB collections was used. Later in this section gene sets are referred to as 'pathways' but there is more than one pathway collection in MigSigDB.

Indeed. We used all human pathways/collections available. We have specified this in the methods section.

(v) In the formula at line 397 doesn't "tested gene sets" refer to "tested gene neighborhoods"? If so, it would be better to use the latter for clarity.

Indeed, we fixed it.

(vi) There appear to be some typos in the caption for Supplementary Figure 1: "we computed three linear models using the different residuals as response variable and SNP interactions as dependent variables". I guess should be "SNP interactions as independent variables".

Thanks, we fix that typo and others present in the caption.

Also, weren't the two individual SNPs also included as independent variables in these models?

Indeed, the two individual SNPs should have been included as independent variables. We updated Supplementary Fig 1 accordingly.
