# Supplementary material for: Interpretable network-guided epistasis detection
Source: Gigascience. 2022 Feb 4;11:giab093. doi: 10.1093/gigascience/giab093 (PMC8848319; doi:10.1093/gigascience/giab093)
Supplement: giab093_Response_to_Reviewer_Comments_Revision_1 [file giab093_response_to_reviewer_comments_revision_1.pdf]

Author's Response To Reviewer Comments

Close

We have included a citation to the data in GigaDB as reference 54.

Close
