# Supplementary material for: Interpretable network-guided epistasis detection
Source: Gigascience. 2022 Feb 4;11:giab093. doi: 10.1093/gigascience/giab093 (PMC8848319; doi:10.1093/gigascience/giab093)
Supplement: giab093_Reviewer_1_Report_Original_Submission — Shing Wan Choi -- 3/24/2021 Reviewed [file giab093_reviewer_1_report_original_submission.pdf]

Reviewer Report

Title: Interpretable network-guided epistasis detection

Version: Original Submission **Date:** 3/24/2021

Reviewer name: Shing Wan Choi

Reviewer Comments to Author:

Here, the authors presented a pipeline for the analysis of epistasis effects in GWAS data (GWAIS). This is traditionally a difficult problem due to the large search space involved, which makes the analysis computationally intensive and suffers from multiple testing, a new method that restrict search space can definitely help making GWAIS more feasible and reproducible. I have some questions after reading the current paper, please excuse me if the information is already presented within the paper:

1. I am not sure what the Standard model comprised of. According to the methodology section, the Standard analyzed all SNP pairs without prior filtering, does that mean all 14,501,130,150 SNP pairs (C(170301, 2)) were tested? Or was it not all SNP pairs were considered?
2. When presenting the number of SNPs linked to each gene based on different criteria (e.g. position, eQTL or Chromatin contact), wouldn't the gene size be a major predictor of the number of SNPs link? It would most likely be the case for positional mapping, right?
3. I am curious to see if restricting the eQTL and Chromatin information to disease specific tissue will help improving the performance of the current model.
4. Very little information was provided for the PRS analysis. What genome wide association summary statistics were used? Did the authors perform high-resolution scoring? With shrinkage / thresholding done in normal PRS analysis, some SNPs' effect might be excluded or "shrinked" away from the PRS model, would that affect the interpretation of the PRS covariate analysis? E.g. maybe SNPs not included in the PRS model were those unaffected? (With PRSice, can use --print-snp to obtain list of SNPs that are included in the model)
5. It seems like Biofilter provide a SNP-SNP interaction prediction model, how does that compare to what was presented here?
6. Figure 3, the results from eQTL + Chromatin and Position + eQTL + Chromatin is identical. Together, it seems like the positional mapping does not contribute to the result at all, which is a bit surprising. Are there any explanation of that? Would it be due to mapping of the Immunochip array, or a characteristic of IBD?
7. Given the sample size of the current data, a HWE threshold of 0.001 seems rather stringent. Will the result improve if a less stringent threshold is used (e.g. 1e-6?)

Methods

Are the methods appropriate to the aims of the study, are they well described, and are necessary controls included? Choose an item.

Conclusions

Are the conclusions adequately supported by the data shown? Choose an item.

Reporting Standards

Does the manuscript adhere to the journal's guidelines on [minimum standards of reporting?]() Choose an item.

Choose an item.

Statistics

Are you able to assess all statistics in the manuscript, including the appropriateness of statistical tests used? Choose an item.

Quality of Written English

Please indicate the quality of language in the manuscript: Choose an item.

Declaration of Competing Interests

Please complete a declaration of competing interests, considering the following questions:

- Have you in the past five years received reimbursements, fees, funding, or salary from an organisation that may in any way gain or lose financially from the publication of this manuscript, either now or in the future?
- Do you hold any stocks or shares in an organisation that may in any way gain or lose financially from the publication of this manuscript, either now or in the future?
- Do you hold or are you currently applying for any patents relating to the content of the manuscript?
- Have you received reimbursements, fees, funding, or salary from an organization that holds or has applied for patents relating to the content of the manuscript?
- Do you have any other financial competing interests?
- Do you have any non-financial competing interests in relation to this paper?

If you can answer no to all of the above, write 'I declare that I have no competing interests' below. If your reply is yes to any, please give details below.

I declare that I have no competing interests

I agree to the open peer review policy of the journal. I understand that my name will be included on my report to the authors and, if the manuscript is accepted for publication, my named report including any attachments I upload will be posted on the website along with the authors' responses. I agree for my

report to be made available under an Open Access Creative Commons CC-BY license (http://creativecommons.org/licenses/by/4.0/). I understand that any comments which I do not wish to be included in my named report can be included as confidential comments to the editors, which will not be published.

Choose an item.

To further support our reviewers, we have joined with Publons, where you can gain additional credit to further highlight your hard work (see: https://publons.com/journal/530/gigascience). On publication of this paper, your review will be automatically added to Publons, you can then choose whether or not to claim your Publons credit. I understand this statement.

Yes Choose an item.
