# Supplementary material for: Interpretable network-guided epistasis detection
Source: Gigascience. 2022 Feb 4;11:giab093. doi: 10.1093/gigascience/giab093 (PMC8848319; doi:10.1093/gigascience/giab093)
Supplement: giab093_Reviewer_2_Report_Original_Submission — Elisabetta Manduchi -- 7/13/2021 Reviewed [file giab093_reviewer_2_report_original_submission.pdf]

Reviewer Report

Title: Interpretable network-guided epistasis detection

Version: Original Submission **Date:** 7/13/2021

Reviewer name: Elisabetta Manduchi

Reviewer Comments to Author:

This manuscript presents a workflow for gene-gene epistasis detection which leverages functional annotation resources such as Biofilter (to reduce the search space) and FUMA (to map SNPs to genes) and investigates the results obtained via different SNP-gene mapping criteria (positional, eQTL, Chromatin contacts, and some combinations of these). Moreover, these results are compared with those obtained via a 'standard' analysis where no filtering is applied to pair selection and positional SNP-gene mapping is used. Due to the challenges presented by GWAIS, leveraging functional genomics to focus the search is a valid strategy which has been employed in other recent works in the field. This is a nice work and the paper is generally well written, with sufficiently detailed methodological information. Below are some comments and questions.

1. As indicated in recent GWAS works aimed at 'solving GWAS loci' (i.e. determining the genes affected by significant SNPs), it is not always the case that the gene affected by a SNP is that positionally closest to the SNP. Indeed, a SNP may not only affect a gene when it resides in its coding or promoter regions, but it may also affect a gene when it resides in a far away enhancer. This is why epigenetic information such as chromatin loops (referred by the authors as 'Chromatin') can be useful for SNP-gene mapping. In the presence of chromatin contacts or eQTL information, typically one would use the derived mapping to augment the positional mapping, which is always available. That is, if one had chromatin contacts data, they would use positional + Chromatin to map SNPs to genes. If one had eQTL data, they would use positional + eQTL to map to genes. If one had both, they would use positional + eQTL + Chromatin. From a biological interpretability perspective, there is no reason to exclude the positional information. For example, a SNP in the promoter of gene could interact with a SNP in a distal enhancer of another gene, affecting a specific trait. In view of this, the statement (lines 326-328) "Since the main objective of this protocol is to increase the biological interpretability of epistasis findings, we have excluded other combinations that mix functional and non-functional information (Positional + eQTL and Positional + Chromatin)" is not quite valid, as positional information is also functional. On the other hand, using eQTL only, Chromatin only, or eQTL + Chromatin, albeit interesting in terms of looking at how this type of reduction in the search space affects results, do not quite reflect a biologically guided approach.

2. I wonder on whether the authors have considered filtering also by markers of relevant chromatin states. Information about open chromatin and other epigenetic marks could help further filtering SNPs, both in enhancers and promoters. This would be particularly useful for SNPs mapped via chromatin contacts, which are likely to contain many irrelevant signals.

3. The eQTL and chromatin contact data used in this work were from all available tissues. Typically, GWAS related functional filtering is done using data from tissues relevant to the trait under investigation, when available. For IBD, it may help to restrict to intestinal tissues, immune cells (like T-

cells, macrophages, dendritic cells), and possibly also nervous system cells (which, at least according to some, could also be among the potential 'culprit' IBD tissues).
4. To adjust for population structure the authors regressed out the first 7 PCs from the phenotype. Given that the PCs are confounders, it would be good to discuss the impact of doing this as opposed to also regress the confounders out of the SNPs, i.e. testing the response residuals vs the SNP residuals. In the same spirit, it would be good to discuss the impact of the PC-SNP association on the p-value and type-I error results obtained by permuting the response residuals.
5. Section 2.1 is somewhat too concise and may result unclear to the reader. Later in the Discussion (lines 229-239) the authors explain how their procedure corrects for multiple testing at the SNP model without additional corrections for multiple testing at the gene model (this is also implicitly described in Fig 7CD), but yet their procedure keeps type I error under control. However, it may be beneficial, for ease of reading, to expand section 2.1 (via text and/or figure) so to clarify better, at the onset, where and when multiple testing corrections are applied.
6. In the absence of a replication data set, the authors assess the robustness of the gene pair results via 10 repetitions of the workflow using 80% of the discovery data set. It would be useful to include some discussion of how their results could be further assessed in other GWAS data sets (e.g. from UK biobank, etc.), in view of the fact that it is typically hard to reproduce epistasis findings, at least at the SNP level. Certainly one could first check whether the discovered SNP-SNP interactions are reproduced and limiting the analyses to those pairs would require a less severe multiple testing correction. But another approach may be to start with the discovered gene pairs and then analyze all pairs of SNPs mapping to these genes (not necessarily those discovered in this study), etc. Do the authors plan future follow-up studies on this?
7. In section 2.7 the results of pathway analyses on 3 (eQTL, Positional, and Standard) of the 5 networks presented in Figure 3 are provided. What about the other 2?
8. For these two points I defer to the editor:
(i) The format of the manuscript is close to but does not exactly match the specifications at https://academic.oup.com/gigascience//pages/research. I do not know how strict these specifications are and I have no objections to the current format.
(ii) Data availability is not discussed (as per Data and materials in https://academic.oup.com/gigascience/pages/instructions_to_authors). I imagine that the IIBDGC only makes publicly available the summary statistics. This is, however, common in the GWAS field.
9. Some minor notes follow:
(i) In the Author Summary the 'ATPM' acronym is used for the first time without explanation.
(ii) In section 4.2 it would be helpful to re-iterate that the SNP-gene mapping for the Standard analysis was genomic proximity (this is only mentioned briefly at line 206).
(iii) Typo at line 168 "the same than" should be "the same as".
(iv) It should be specified which of the MigSigDB collections was used. Later in this section gene sets are referred to as 'pathways' but there is more than one pathway collection in MigSigDB.
(v) In the formula at line 397 doesn't "tested gene sets" refer to "tested gene neighborhoods"? If so, it would be better to use the latter for clarity.
(vi) There appear to be some typos in the caption for Supplementary Figure 1: "we computed three linear models using the different residuals as response variable and SNP interactions as dependent

variables". I guess should be "SNP interactions as independent variables". Also, weren't the two individual SNPs also included as independent variables in these models?

Methods

Are the methods appropriate to the aims of the study, are they well described, and are necessary controls included? Choose an item.

Conclusions

Are the conclusions adequately supported by the data shown? Choose an item.

Reporting Standards

Does the manuscript adhere to the journal's guidelines on minimum standards of reporting? Choose an item.

Choose an item.

Statistics

Are you able to assess all statistics in the manuscript, including the appropriateness of statistical tests used? Choose an item.

Quality of Written English

Please indicate the quality of language in the manuscript: Choose an item.

Declaration of Competing Interests

Please complete a declaration of competing interests, considering the following questions:

- Have you in the past five years received reimbursements, fees, funding, or salary from an organisation that may in any way gain or lose financially from the publication of this manuscript, either now or in the future?
- Do you hold any stocks or shares in an organisation that may in any way gain or lose financially from the publication of this manuscript, either now or in the future?
- Do you hold or are you currently applying for any patents relating to the content of the manuscript?
- Have you received reimbursements, fees, funding, or salary from an organization that holds or has applied for patents relating to the content of the manuscript?
- Do you have any other financial competing interests?
- Do you have any non-financial competing interests in relation to this paper?

If you can answer no to all of the above, write 'I declare that I have no competing interests' below. If your reply is yes to any, please give details below.

I declare that I have no competing interests

I agree to the open peer review policy of the journal. I understand that my name will be included on my report to the authors and, if the manuscript is accepted for publication, my named report including any attachments I upload will be posted on the website along with the authors' responses. I agree for my report to be made available under an Open Access Creative Commons CC-BY license (http://creativecommons.org/licenses/by/4.0/). I understand that any comments which I do not wish to be included in my named report can be included as confidential comments to the editors, which will not be published.

Choose an item.

To further support our reviewers, we have joined with Publons, where you can gain additional credit to further highlight your hard work (see: https://publons.com/journal/530/gigascience). On publication of this paper, your review will be automatically added to Publons, you can then choose whether or not to claim your Publons credit. I understand this statement.

Yes Choose an item.
