# Supplementary material for: Interpretable network-guided epistasis detection
Source: Gigascience. 2022 Feb 4;11:giab093. doi: 10.1093/gigascience/giab093 (PMC8848319; doi:10.1093/gigascience/giab093)
Supplement: giab093_Reviewer_2_Report_Revision_1 — Elisabetta Manduchi -- 10/24/2021 Reviewed [file giab093_reviewer_2_report_revision_1.pdf]

Reviewer Report

Title: Interpretable network-guided epistasis detection

Version: Revision 1 **Date:** 10/24/2021

Reviewer name: Elisabetta Manduchi

Reviewer Comments to Author:

The authors have made several improvements to their manuscript which adequately address most of my original review's comments. I also like the idea of using a circular layout for networks to facilitate comparisons. There are a couple of suggestions that I would like to bring to the authors' attention:
1. In their response to my original point (4) regarding confounders (in this case the PCs), both in terms of considering regressing them also out of the SNPs and in terms of the permutation procedure, the authors indicate that their options were restricted and they made pragmatic choices which may be suboptimal. I accept this, but I think it would be worth adding a paragraph to the Discussion to indicate such limitations.
2. In the second paragraph of the Discussion, the authors state:
"Moreover, the different mappings unevenly described genes... This motivated combining multiple mappings into an analysis (Positional + eQTL + Chromatin)..."
The way this is written hides what should be the actual motivation of combining Positional +eQTL + Chromatin. The main motivation, in my opitnion, should be the biology, as I indicated in my previous review. For example, a SNP in the promoter of gene could interact with a SNP in a distal enhancer of another gene, affecting a specific trait.
Some minor comments and typos:
1. In section "Analysis -> SNP to gene mapping:...", in the sentence "the number of SNPs mapped to a same gene varied considerably across analyses", it may be clearer to replace 'analyses' by 'mapping methods'.
3. Fig. 3 is missing panels corresponding to the Pos+ eQTL + Chromatin mapping.
4. In Fig 5, the captions for panels A and B are swapped. Also, there is a typo: 'using with and without filtering'.
5. The title of the section "Chromatin and Standard analyses partially replicate previous studies on IBD" should be changed in view of the content. Here the replications discussed are those for the Standard and the Pos + eQTL + Chromatin analyses, not for the Chromatin only analysis.
6. The authors do not seem to have conducted the tissue-specific analyses for the Pos + eQTL + Chromatin mapping.
7. Supplementary Table 3, cell in row 1 col 3, typo in "mappable a Biofilter gene-pair" (missing 'to').

Methods

Are the methods appropriate to the aims of the study, are they well described, and are necessary controls included? Choose an item.

Conclusions

Are the conclusions adequately supported by the data shown? Choose an item.

Reporting Standards

Does the manuscript adhere to the journal's guidelines on minimum standards of reporting? Choose an item.

Choose an item.

Statistics

Are you able to assess all statistics in the manuscript, including the appropriateness of statistical tests used? Choose an item.

Quality of Written English

Please indicate the quality of language in the manuscript: Choose an item.

Declaration of Competing Interests

Please complete a declaration of competing interests, considering the following questions:

- Have you in the past five years received reimbursements, fees, funding, or salary from an organisation that may in any way gain or lose financially from the publication of this manuscript, either now or in the future?
- Do you hold any stocks or shares in an organisation that may in any way gain or lose financially from the publication of this manuscript, either now or in the future?
- Do you hold or are you currently applying for any patents relating to the content of the manuscript?
- Have you received reimbursements, fees, funding, or salary from an organization that holds or has applied for patents relating to the content of the manuscript?
- Do you have any other financial competing interests?
- Do you have any non-financial competing interests in relation to this paper?

If you can answer no to all of the above, write 'I declare that I have no competing interests' below. If your reply is yes to any, please give details below.

I declare that I have no competing interests

I agree to the open peer review policy of the journal. I understand that my name will be included on my report to the authors and, if the manuscript is accepted for publication, my named report including any attachments I upload will be posted on the website along with the authors' responses. I agree for my

report to be made available under an Open Access Creative Commons CC-BY license (http://creativecommons.org/licenses/by/4.0/). I understand that any comments which I do not wish to be included in my named report can be included as confidential comments to the editors, which will not be published.

Choose an item.

To further support our reviewers, we have joined with Publons, where you can gain additional credit to further highlight your hard work (see: https://publons.com/journal/530/gigascience). On publication of this paper, your review will be automatically added to Publons, you can then choose whether or not to claim your Publons credit. I understand this statement.

Yes Choose an item.
